# Application of protoplast technology to CRISPR/Cas9 mutagenesis: from single‐cell mutation detection to mutant plant regeneration

**DOI:** 10.1111/pbi.12870

**Published:** 2018-01-10

**Authors:** Choun‐Sea Lin, Chen‐Tran Hsu, Ling‐Hung Yang, Lan‐Ying Lee, Jin‐Yuan Fu, Qiao‐Wei Cheng, Fu‐Hui Wu, Han C.‐W. Hsiao, Yesheng Zhang, Ru Zhang, Wan‐Jung Chang, Chen‐Ting Yu, Wen Wang, Li‐Jen Liao, Stanton B. Gelvin, Ming‐Che Shih

**Affiliations:** ^1^ Agricultural Biotechnology Research Center Academia Sinica Taipei Taiwan; ^2^ Department of Biological Sciences Purdue University West Lafayette IN USA; ^3^ Department of Bioinformatics and Medical Engineering Asia University Taichung City Taiwan; ^4^ State Key Laboratory of Genetic Resources and Evolution Kunming Institute of Zoology Chinese Academy of Sciences Kunming China; ^5^ Institute of Life Science National Kaohsiung Normal University Kaohsiung Taiwan; ^6^ Present address: Department of Biochemistry University of Pennsylvania Philadelphia PA 19104‐6030 USA

**Keywords:** protoplast isolation, CRISPR/Cas9, protoplast regeneration, single‐cell analysis

## Abstract

Plant protoplasts are useful for assessing the efficiency of clustered regularly interspaced short palindromic repeats (CRISPR)/CRISPR‐associated protein 9 (Cas9) mutagenesis. We improved the process of protoplast isolation and transfection of several plant species. We also developed a method to isolate and regenerate single mutagenized *Nicotianna tabacum* protoplasts into mature plants. Following transfection of protoplasts with constructs encoding Cas9 and sgRNAs, target gene DNA could be amplified for further analysis to determine mutagenesis efficiency. We investigated *N*. *tabacum* protoplasts and derived regenerated plants for targeted mutagenesis of the *phytoene desaturase* (*NtPDS*) gene. Genotyping of albino regenerants indicated that all four *NtPDS* alleles were mutated in amphidiploid tobacco, and no *Cas9* DNA could be detected in most regenerated plants.

## Introduction

Genome engineering is an important component of the relatively novel field of synthetic biology. Gene editing, the directed change of a specific DNA sequence, is an important element of genome engineering. The clustered regularly interspaced short palindromic repeats (CRISPR)/CRISPR‐associated protein 9 (Cas9) system is a convenient genome‐editing tool that requires only two reagents: Cas9 protein and a single guide RNA (sgRNA) (Feng *et al*., 2013; Gaj *et al*., 2013; Li *et al*., 2013; Nekrasov *et al*., 2013; Shan *et al*., 2013). Following CRISPR‐mediated mutagenesis, integrated transgenes encoding gene editing reagents can often be removed from the genome through genetic segregation, mitigating public concerns regarding genetically modified organisms (Huang *et al*., 2016). CRISPR/Cas9 mutagenesis is therefore becoming an important technology for basic plant science and agriculture.

Numerous published studies have described various vectors to improve CRISPR‐mediated target mutagenesis. These studies describe parameters such as promoters, different versions of Cas9, and the use of multiple sgRNAs (Ali *et al*., 2015, 2016; Belhaj *et al*., 2013; Bortesi and Fischer, 2015; Butt *et al*., 2017; Cermak *et al*., 2017; Eid *et al*., 2016; Kaya *et al*., 2016; Ma *et al*., 2015; Murovec *et al*., 2017; Shimatani *et al*., 2017; Wang *et al*., 2015b; Yan *et al*., 2015). Not only Cas9, but other endonucleases such as Cpf1 can induce mutations (Endo *et al*., 2016; Kim *et al*., 2017; Mahfouz, 2017; Xu *et al*., 2017). However, stable transformation to evaluate CRISPR mutagenesis efficacy can be time‐consuming. Transient protoplast transfection is an alternative strategy to test multiple mutagenesis parameters rapidly. Protoplasts from at least five crop species (rice, wheat, maize, lettuce, and tomato), in addition to *Arabidopsis* and tobacco, have been used to evaluate gene editing reagents using CRISPR/Cas9‐based systems (Cermak *et al*., 2015; Liang *et al*., 2014; Shan *et al*., 2014; Woo *et al*., 2015). Although several methods have been developed to obtain and transfect *Arabidopsis* protoplasts (Sheen, 2001; Wu *et al*., 2009), protoplast isolation remains a bottleneck to testing genome‐editing reagents in many crop species.

Previously, DNA from pooled mutagenized protoplasts was used to determine target site mutagenesis efficiency (Cermak *et al*., 2015; Liang *et al*., 2014; Shan *et al*., 2014; Woo *et al*., 2015). The target region is amplified by PCR, and the resulting amplicons are further evaluated using restriction fragment length polymorphism (RFLP) (Feng *et al*., 2013; Nekrasov *et al*., 2013; Shan *et al*., 2013) or cleaved amplified polymorphic sequence analysis (Kaya *et al*., 2016; Mikami *et al*., 2015a,b; Shimatani *et al*., 2017), T7 endonuclease I (T7E1) analysis (Kim *et al*., 2017; Woo *et al*., 2015) or next‐generation sequencing (NGS) (Kim *et al*., 2017; Woo *et al*., 2015). Because the PCR amplicons constitute a mixture of wild‐type and mutated DNA, mutagenesis efficiency is determined by calculating the gel image density (RFLP and T7E1 assays) or the per cent of mutant sequences (by NGS). It is often difficult to detect low target site mutagenesis efficiency. False‐positive results can occur because of incomplete restriction endonuclease digestion or PCR errors. Although NGS can resolve these problems, the process can be time‐consuming and costly. Because there are only two potential target site alleles in a diploid cell, single‐cell DNA analysis can rapidly determine mutagenesis efficiency. Several single‐cell isolation methods have been published (Brennecke *et al*., 2013; Efroni and Birnbaum, 2016; Efroni *et al*., 2015; Gierahn *et al*., 2017; Klein *et al*., 2015; Macosko *et al*., 2015; Yamamoto *et al*., 2016). Single‐cell analyses have been applied to transcriptome and metabolome studies (Efroni *et al*., 2015; Yamamoto *et al*., 2016). However, these studies required expensive facilities or technically demanding protocols, including flow cytometry (Gierahn *et al*., 2017; Klein *et al*., 2015; Macosko *et al*., 2015) or microinjection (Yamamoto *et al*., 2016). A convenient and reliable single‐cell isolation protocol would greatly benefit plant scientists conducting gene editing experiments.

Protoplasts can be used to determine target site mutagenesis efficiency and can be regenerated into plants (Woo *et al*., 2015). Furthermore, genome‐editing reagents such as sgRNAs and Cas9 protein can be synthesized and assembled *in vitro* to form active ribonucleoprotein (RNP) complexes. These complexes can be delivered into protoplasts and mutagenize the target gene. Thus, target mutants can be obtained without the presence of exogenous DNA (Kim *et al*., 2017; Liang *et al*., 2017; Woo *et al*., 2015). Such DNA‐free genome editing avoids stable introduction of transgenes. Although geminiviruses can be used in whole plants to deliver donor DNA for homology‐directed repair (HDR; Wang *et al*., 2017), protoplast transfection is an alternative to deliver high amounts of DNA required for HDR. However, protoplast regeneration is difficult in most plant species.

In this report, we further develop protoplast isolation protocols for several crop and ornamental species, and the model plant *Arabidopsis*. We used these protoplasts to evaluate CRISPR/Cas9 mutagenesis efficiency. We describe a simple single‐protoplast isolation protocol and use this protocol to edit the tobacco *NtPDS* gene. Multiple plants regenerated from single mutagenized tobacco protoplasts contain a variety of CRISPR‐induced mutations.

## Results

### Improvement of protoplast isolation for transfection

**Figure 1:**
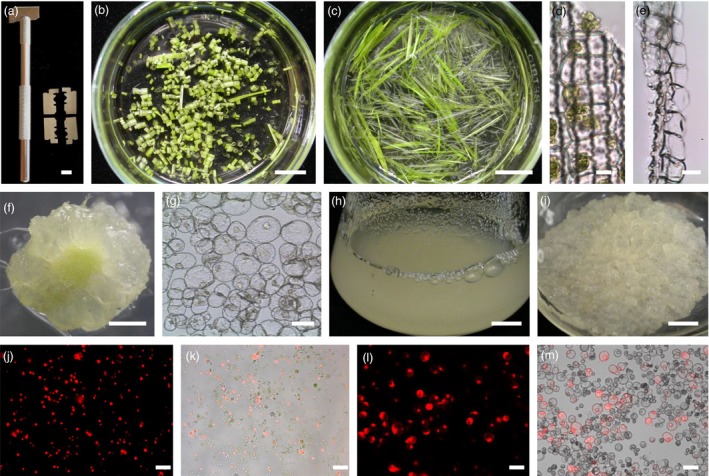
Improved protoplast isolation. (a) A razor blade was divided into four pieces and the pieces assembled in parallel in a scalpel handle. (b) Seedlings of rice were cut in cross section and placed in digestion solution. Bar = 1 cm. (c) Seedlings of rice were cut longitudinally (in the same direction as the veins) and placed in digestion solution. Bar = 1 cm. (d) Microscopic image of rice seedlings cut in cross section (perpendicular to vascular bundles) after 3‐h digestion. Bar = 10 μm. (e) Microscopic image of rice seedlings cut in longitudinally (parallel to vascular bundles) after 3‐h digestion. Bar = 10 μm. (f) Tomato hypocotyl sections were incubated in medium supplemented with 10 mg/L NAA and photographed after 1 month. Bar = 1 cm. (g) Microscopic image of tomato suspension cells. Bar = 40 μm. (h) Tomato suspension cells grown in 1 mg/L 2,4‐D after 7 days. Bar = 1 cm. (i) Tomato suspension cells formed calli on solid medium supplemented with 1 mg/L 2,4‐D. Bar = 1 cm. (j) mRFP‐NLS plasmid DNA was delivered to rice protoplasts using a PEG‐mediated method. Protoplasts were photographed after 24 h. Red colour indicates RFP epifluorescence. Bar = 50 μm. (k) Overlay of epifluorescence and bright field images of transfected rice protoplasts. Bar = 50 μm. (l) mRFP‐NLS plasmid DNA was delivered into tomato Micro‐Tom protoplasts using a PEG‐mediated method. Protoplasts were photographed after 24 h. Red colour indicates RFP epifluorescence. Bar = 50 μm. (m) Overlay of epifluorescence and bright field images of transfected tomato protoplasts. Bar = 50 μm.

We designed a tool to generate multiple longitudinal cuts in monocot seedlings. A razor blade was divided into four pieces, which were stacked in parallel on a scalpel handle (Figure 1a). In previous reports, seedlings were cut in cross section (Zhang *et al*., 2011; Figure 1b). In this report, seedlings were sorted and arranged in parallel (or affixed to clear tape) for longitudinal cutting (Figure 1c). When a seedling is cut in cross section, only the cells near the edge are digested (Figure 1d); cutting in longitudinal sections increases the release of protoplasts (Figure 1e). The protoplast yield from rice seedlings subjected to longitudinal cutting was higher than that from seedlings subjected to cross‐cutting [4.8 × 10^6^ protoplasts/g fresh weight (FW) vs 2.2 × 10^6^ protoplasts/g FW]. Longitudinal cutting permitted efficient cell wall digestion by cellulose R10 and macerozyme R10 enzymes (Table S1), which are less expensive than are cellulase RS and macerozyme RS enzymes used previously (Zhang *et al*., 2011). Longitudinal cutting was used successfully for five Poaceae species: rice, wheat, maize, millet and bamboo. Protoplasts derived from this protocol were transfected with a red fluorescence protein (RFP) gene by a PEG‐mediated method. Total intact cell number was calculated, and the transfection efficiency was calculated as the percentage of intact protoplasts with RFP fluorescence/intact protoplasts. Transfection efficiencies of these protoplasts were >40% (Figure 1j and k, bamboo: 54%; millet: 51%; rice: 44%; maize: 47%; wheat: 41%; Figure S1).

We previously established an efficient ‘Tape‐*Arabidopsis* Sandwich’ protoplast isolation protocol (Wu *et al*., 2009). This protocol can be applied to several Brassicaceae species, including *Brassica oleracea*,* B. napus*,* Cleome spinosa*,* C. monophilla*, and *C. gynadra*. Using 3M Scotch Tape, we peeled off the epidermal layer of leaves or cotyledons, facilitating protoplast release. Our results indicated that 2‐ to 3‐week‐old *in vitro* cotyledons from *B. napus* were more suitable for protoplast isolation than were 1‐week‐old cotyledons. For *Cleome* species, mature leaves from plants grown in a greenhouse were suitable. *C. gynadra* is a C4 plant, so there are two types of protoplasts, from mesophyll and bundle sheath cells. Protoplasts from all six Brassicaceae species isolated by the Tape Sandwich method were transfected using a PEG‐mediated method with efficiencies >40% (Figure S1; *Arabidopsis*: 67%; broccoli: 43%; rapeseed: 63%; *C. gynandra*: 46%; *C. spinosa*: 79%; *C. monophilla*: 83%).

It was difficult to isolate high‐quality protoplasts from tomato leaves using the Tape Sandwich method (data not shown). Instead, a suspension cell line was developed from tomato hypocotyls using the tomato cultivar ‘Micro‐Tom’ (Figure 1f–i). Tomato hypocotyls were cut, callus cultures were developed, and the callus used to establish a suspension cell culture (Figure 1g,h). The proliferation rate of this tomato suspension line is similar to that of tobacco BY‐2 cells. Protoplasts were isolated by incubating the suspension cells in a cell wall digestion buffer. Similar to BY‐2 protoplasts, the tomato protoplasts were transfected using a PEG‐mediated transfection method with 63% efficiency (Figures 1l,m, and S1).

### CRISPR/Cas9‐mediated mutagenesis of protoplasts

**Figure 2:**
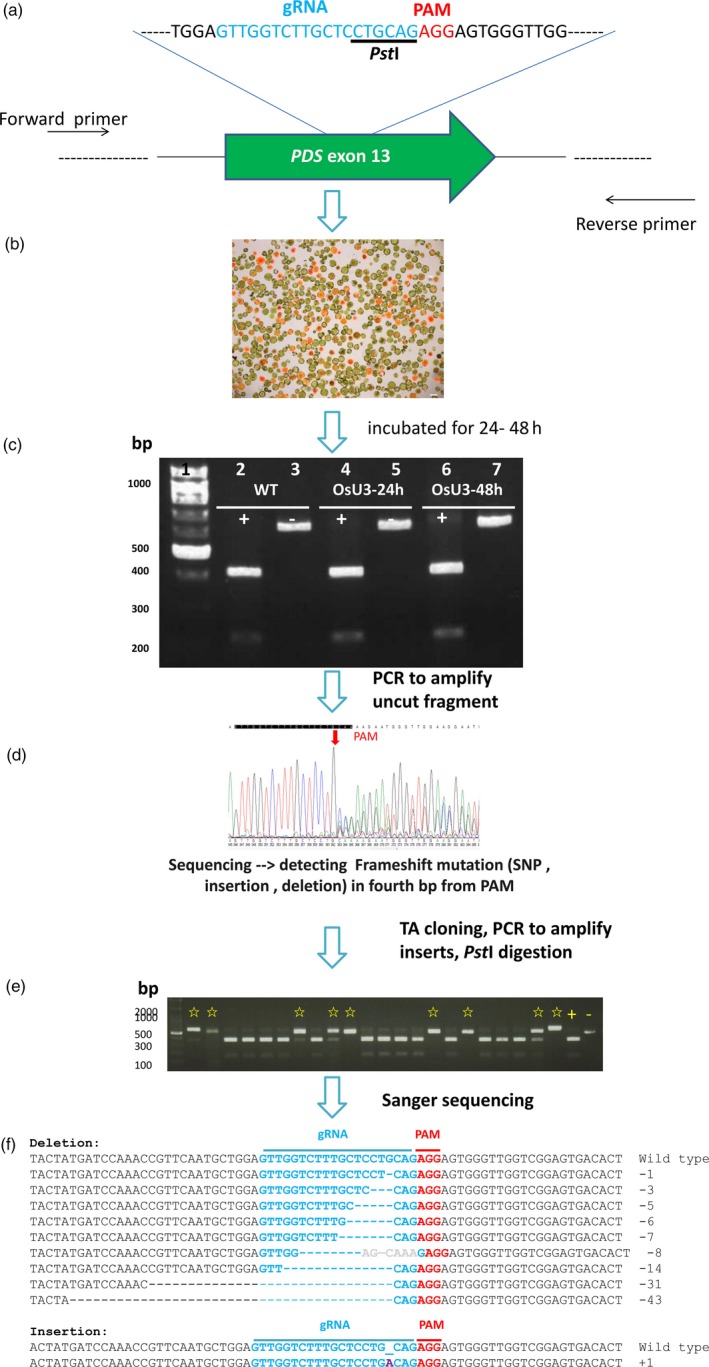
Schematic showing mutation of target site sequences after genome‐editing using a protoplast transfection system. (a) The genomic sequence of the target gene is used to design an sgRNA sequence and primers. Target sites containing a restriction enzyme site are chosen. Blue, Target site; red, protospacer adjacent motif (PAM). The single guide RNA (sgRNA) was cloned into pCAMBIA1300‐OsU3‐Cas9 (or OsU6). Plasmid DNA is delivered into protoplasts by a PEG‐mediated method. (b) Fluorescence image of transfected millet protoplasts. The plasmid mRFP‐NLS is used as a marker to determine the transfection efficiency. Cells fluorescing red are successfully transfected. (c) DNA from nontransfected (WT) or transfected (OsU3‐24 h or OsU3‐48 h) protoplasts are isolated and used as a PCR template for target gene amplification (*PDS* in this case). PCR products with (+) or without (−) *Pst*I digestion. (d) Sanger method sequencing result of one aliquot pool of PCR product. The PCR product pool contains a mixture of wild‐type and differentially mutagenized DNA [SNPs (single nucleotide polymorphisms), insertions and deletions]. (e) An aliquot of mutagenized PCR products is subcloned into a T/A cloning vector. The inserts of individual colonies are amplified by colony PCR and the mutation confirmed by digestion with *Pst*I. Clones containing a putative mutagenized target gene (indicated by star marks) are subjected to sequencing. (f) DNA sequencing results of mutated clones. First line, wild type; blue, target site; red, PAM; purple, insertion; −, deleted nucleotides.

To evaluate mutagenesis efficiency, we targeted in most species the phytoene desaturase (*PDS*) gene. For each *PDS* gene, we chose the sgRNA such that a restriction enzyme site upstream of the protospacer adjacent motif (PAM; Figure 2a) sequence may be lost if target mutations occurred. If there were no suitable sgRNA targeting site (e.g. lacking a PAM sequence) or unique sequence for *PDS*, a published sgRNA for another gene was used.

Protoplasts from each species were transfected with a VirD2‐NLS‐mRFP plasmid (Lee *et al*., 2008) and pCAMBIA1300‐OsU3(*Aar*I)‐Cas9 or pCAMBIA1300‐OsU6(*Aar*I)‐Cas9 carrying the species‐specific sgRNA (Figures 2b and S2). After 24 or 48 h, DNA from total protoplasts was extracted and the target gene amplified by PCR. The PCR product was digested with a restriction enzyme whose site is adjacent to the PAM, and the products separated by electrophoresis through an agarose gel (Figure 2c). Undigested DNA appeared in the 48 h sample. This result suggests that some genomic DNA had been mutated, disrupting the restriction endonuclease site targeted by the sgRNA. The undigested PCR fragments were cloned into a T/A vector, transformed into *Escherichia coli*, and the insert subjected to colony PCR. The PCR fragment was digested by the appropriate restriction endonuclease, and PCR products not digested by the restriction enzyme were sequenced (Figure 2d–f). Protoplast transfection and CRISPR/Cas9 editing were evaluated for each of the nine species. The results are summarized in Table 1.

**Table 1:** Targeted mutations in protoplasts of nine plant species

	Species	Transfection efficiency (%)	Target gene	Mutagenesis efficiency (%)	Sequenced mutations (bp)		
					Insertions	Deletions	Other
Poaceae	*Bambusa oldhamii*	54	*PDS*	6.6	—	1, 3, 4, 6, 8, 11, 13	Replace 1
	*Setaria italica*	51	*PDS*	10.2	1	1, 3, 5, 6, 7, 8, 14, 31, 43	—
	*Oryza sativa*	44	*PDS*	7.3	1, 175	1, 9, 11	—
	*Zea mays*	47	*IPK*	0.2^a^	1	5	—
				1.1^b^	1	1	—
Brassicaceae	*Arabidopsis thaliana*	67	*PDS*	6.5	1(A), 1(T), 2	−5	—
	*Brassica oleracea*	43	*GA4a*	75.2	1 (C), 1 (T)	2, 5, 6, 8, 10, 14	Delete 5, replace 5
	*Brassica napus*	63	*GA4a*	56.8	1 (A), 1 (T), 34	2, 5	—
Solanaceae	*Nicotiana tabacum*	41	*PDS*	15.0	14, 24	1, 3, 6, 8, 12	—
	*Solanum lycopersicum*	63	*PDS*	3.7	29	1, 2, 3, 8, 9	—

a *sgRNA1*.

b *sgRNA2*.

### CRISPR editing of *Poaceae* protoplasts

#### Bamboo

For the bamboo *PDS* gene, a target sequence (sg2) that contains a *Bsa*I restriction site was chosen. Results of the DNA amplifications and digestions after the transfection are shown in Figure S3. After 48‐h transfection, there was a minor undigested band indicating a mutation efficiency of only 6.6%. {The intensity of the undigested band is 52.800 arbitrary units (au). The sum of the intensities of the digested bands is 1434.911 au. The transfection efficiency is 54%. Therefore, the mutation frequency is [52.8/(52.8 + 1434.911)]/0.54 = 6.6%.}. The PCR products amplified by the first set of primers were cloned, and clones with putative mutated regions were sequenced. Five of 40 clones showed mutations (12.5%), which were either deletions (1–13 bp) or a 1‐bp substitution (Figure S4a). Although base substitutions are relatively rare, several studies reported such CRISPR/Cas9‐induced mutations (Cermak *et al*., 2015; Ikeda *et al*., 2016; Li *et al*., 2013; Liang *et al*., 2016; Mikami *et al*., 2015a,b; Schiml *et al*., 2014; Wang *et al*., 2016; Xu *et al*., 2014; Zhang *et al*., 2014). To reduce the cloning work and increase the sensitivity of mutant validation, we amplified DNA (using a second set of primers) from the restriction enzyme mixture and digested the amplicons with *Bsa*I to detect mutations again. Meanwhile, the second PCR products were cloned into a T/A vector (Figure S4b). After enrichment of the mutated amplified DNA, 71% (5/7) of the clones carried mutations, all of which were deletions (3–8 bp).

#### Millet

The results in millet were similar to those of bamboo, with a more significant undigested band in protoplast DNA PCR products after 48 h (Figure S5). The scanned electrophoresis image indicated a mutation frequency of 10.2% after 48‐h incubation (Figure S5a). Sequencing results of the second PCR product indicated that most of the mutant clones carried deletions before the PAM, with one showing an insertion and one showing an insertion/deletion (line 7, the sequence with the grey colour code; Figure S5c).

#### Rice

The transfection of rice protoplasts with pCAMBIA1300‐OsU3(*Aar*I)‐OsPDS resulted in a minor undigested band after DNA amplification 24 h after transfection and a more visible band after 48 h (Figure S6). The scanned gel image indicated a mutation frequency of 7.3%. The second PCR product cloning and sequencing showed mutations at the predicted site (the fourth nucleotide before the PAM). Most mutations were deletions. There were two cases of insertions; one was a 1‐bp insertion, and the other contained 175 nucleotides of vector sequence (Figure S6c).

#### Maize

The maize inositol phosphate kinase (*IPK*) gene was targeted using two sgRNA sequences (Liang *et al*., 2014). These sgRNAs were cloned individually into pCAMBIA1300‐OsU3(*Aar*I). *Sac*I and *Bam*HI were used to validate mutations effected by gRNA1 and gRNA2, respectively. After 48‐h transfection, there were minor undigested bands in both gRNA1 and 2 treatments, indicating that the mutation efficiency was low. When individual gRNAs were used for transfection, gRNA2 was more efficient than was gRNA1 48 h after transfection (Figure S7, mutation frequency, gRNA1: 0.2%; gRNA2: 1.1%). Sequencing revealed mutations consisting of either deletions or insertions (Figures S7c and d). When both guide RNAs were co‐transfected, the first PCR did not reveal any truncated product. However, there was a ~300‐bp smaller PCR product after the second PCR (Figure S8a). These results confirmed that the DNA region between sgRNA1 and 2 was deleted (Figure S8b).

### CRISPR editing of *Brassicaceae* protoplasts

#### *Arabidopsis*

*Arabidopsis* protoplasts were transfected with pCAMBIA1300‐OsU3(*Aar*I)‐AtPDS3. Although the AtPDS3 sgRNA was driven by either a rice OsU3 or OsU6 promoter, CRISPR/Cas9‐mediated mutations were detected. Using the construct carrying the OsU3 promoter, DNA extracted from protoplasts amplified with AtPDS3 primers and digested with *Nco*I showed faint undigested bands 24 h after transfection (Figure S9a). The results were similar when the OsU6 promoter was used (Figure S9b). After 48 h, more intense undigested bands were observed. The mutation frequency was 6.5% in the OsU6 treatment after 48‐h incubation. The PCR products were cloned and sequencing indicated that mutations (deletions and insertions) initiated at or before the fourth nucleotide before the PAM (Figure S9d).

#### Broccoli

For broccoli, we targeted the sgRNA gene *BolGA4.a* at a *Hph*I site (Lawrenson *et al*., 2015). Both rice promoters, U3 and U6, were tested. Despite the use of a monocot promoter, genome editing in *B.  oleracea* had a 75.2% mutation efficiency with OsU6 treatment after 48‐h incubation (Figure S10). To confirm that the undigested PCR product is mutated by CRISPR/Cas9, the first PCR product was cloned and sequenced. The results indicated that 64.1% (25/39) of the clones were mutated at or before the fourth nucleotide preceding the PAM (Figure S11). These results are similar to those of the gel image. Using these two methods, both mutagenesis efficiency and the mutated sequences could be obtained.

#### Rapeseed

Because the rapeseed *GA4.a* gene is highly homologous to that of broccoli, the same construct was used for both species. The CRISPR/Cas9 editing efficiency was also high (in OsU3 treatment after 24‐h incubation, 56.8%) in rapeseed protoplasts. The first PCR product was cloned and sequenced. Sequencing indicated both deletions and insertions (Figure S12).

### CRISPR editing of *Solanaceae* protoplasts

To edit Solanaceous crop genomes, we used tobacco (BY‐2) and tomato (Micro‐Tom) suspension cells. The *PDS* homologs, which harbour a *Mly*I site, were targeted in both species. The OsU3 promoter was used to drive sgRNA expression in both cases. The results are shown in Figure S13 (for tobacco BY‐2 cells, the mutation frequency was 15.0% after 48 h) and Figure S14 (for tomato Micro‐Tom, the mutation frequency was 3.7% after 48 h).

### Detecting CRISPR‐mediated mutations in individual protoplasts

Transfection of protoplasts with CRISPR editing reagents usually results in a mixture of genotypes, including nonedited wild‐type sequences and various differently edited genomes. Even when the protoplast transfection efficiency is high, frequently only a small percentage of cells contain edited genomes. It is often difficult to detect these rare edited genomes among a high background of nonedited genomes. We thus developed a protocol for analysing DNA from individual mutagenized protoplasts. Because the PCR template is from a single cell, it is easier to validate the electrophoresis results, including complete digestion of the amplicon DNA by restriction endonucleases. Mutations in the PCR product can be confirmed by Sanger sequencing.

We evaluated the parameters of transfection and single‐cell analysis by targeting mutations in the tobacco *PDS* gene (*NtPDS*). Tobacco (*N.  tabacum*) is an amphidiploid derived from *N. sylvestris* and *N. tomentosiformis* (Endo *et al*., 2016; Sierro *et al*., 2014). Specific primers were designed to amplify *PDS* genes from the *N*. *sylvestris* genome (S form) and the *N*. *tomentosiformis* genome (T form). We transfected *N. tabacum* protoplasts with various amounts of a mixture of a construct encoding the sgRNA which can target identical sequences in both genomes and the Cas9 protein (Kaya *et al*., 2016) and isolated more than 20 individual protoplasts for each treatment.

**Figure 3:**
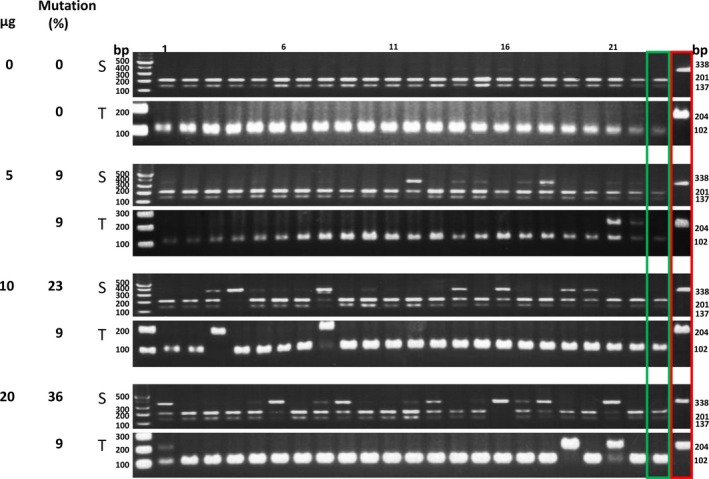
Single‐protoplast analysis of the effect of plasmid dosage on *NtPDS* target mutagenesis. Different amounts of plasmid DNA (containing the expression cassette of *NtPDS* sgRNA and *SaCas9*; Kaya *et al*., 2016) were transfected into tobacco mesophyll protoplasts. After three days, the target mutations were analysed by RFLP. S, *N*. *sylvestris* form; T, *N. tomentosiformis* form. Green box, wild‐type RFLP control; red box, albino mutant RFLP control. Numbers above the gel lanes indicate protoplast number.

**Figure 4:**
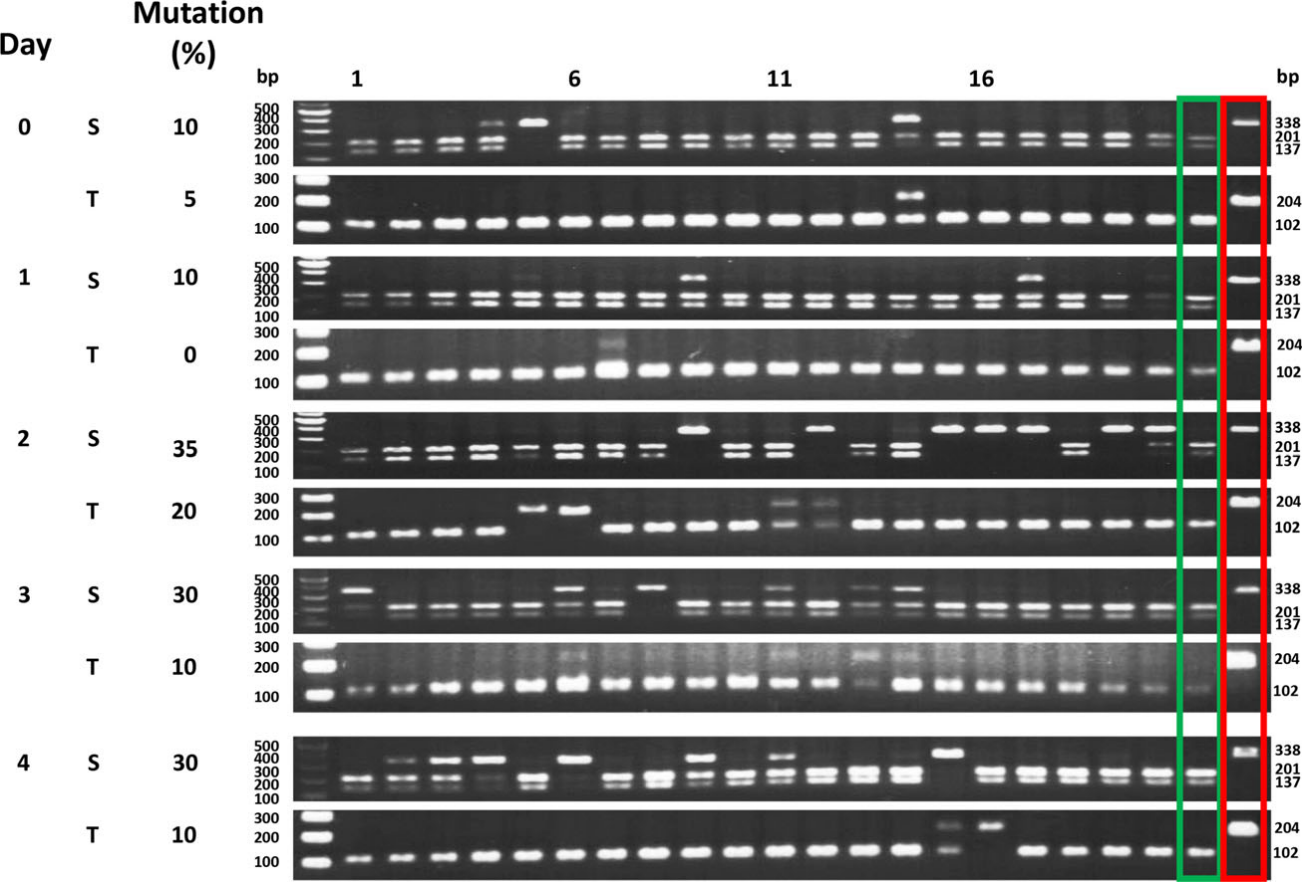
Single‐protoplast analysis of the effect of incubation times on *NtPDS* target mutagenesis. Tobacco protoplasts were transfected with 20 μg plasmid DNA (containing the expression cassette of *NtPDS* sgRNA and *SaCas9*; Kaya *et al*., 2016) and incubated for various number of days. The target mutation was analysed by RFLP. S, *sylvestris* form; T, *tomentosiformis* form. Green box, wild‐type RFLP control; red box, albino mutant RFLP control. Numbers above the gel lanes indicate protoplast number.

Table 2 shows that with increasing amounts of DNA, a higher percentage of individual protoplasts showed *PDS* mutations. Using 20 μg of DNA, on average 55.3% of the protoplasts showed S form target mutations in three experiments. Because the transfection efficiency in this experiment (Figure 3) was 43%, these results indicate that the S genome *PDS* target mutation efficiency was 83.7% (0.36/0.43 = 83.7%, Data S1). Although the target sequence was identical, only 29.7% of the T form *PDS* genes showed mutations (Table 2). Similar S/T mutation ratios were seen when protoplasts were incubated for 2–4 days following transfection (Figure 4, Data S2). Triplicate experiments yielded similar results (Figure S15, Data S2).

**Table 2:** Mutation of *NtPDS* genes from single transfected tobacco protoplasts

DNA (μg)	Gene^a^	0	5	10	20
Experiment 1	S	0	9	23	36
	T	0	9	9	9
Experiment 2	S	0	65	70	85
	T	0	15	40	50
Experiment 3	S	0	10	60	45
	T	0	5	25	30
% Target mutation (±SE)	**S**	**0**	**28.0 **±** 32.0**	**51.0 **±** 24.8**	**55.3 **±** 26.1**
	**T**	**0**	**9.7 ± 5.0**	**24.7 ± 15.5**	**29.7 ± 20.5**

a S, *N. sylvestris* gene; T, *N. tomentosiformis* gene. Bold‐face type indicates the average±s.d. among the multiple experiments.

**Figure 5:**
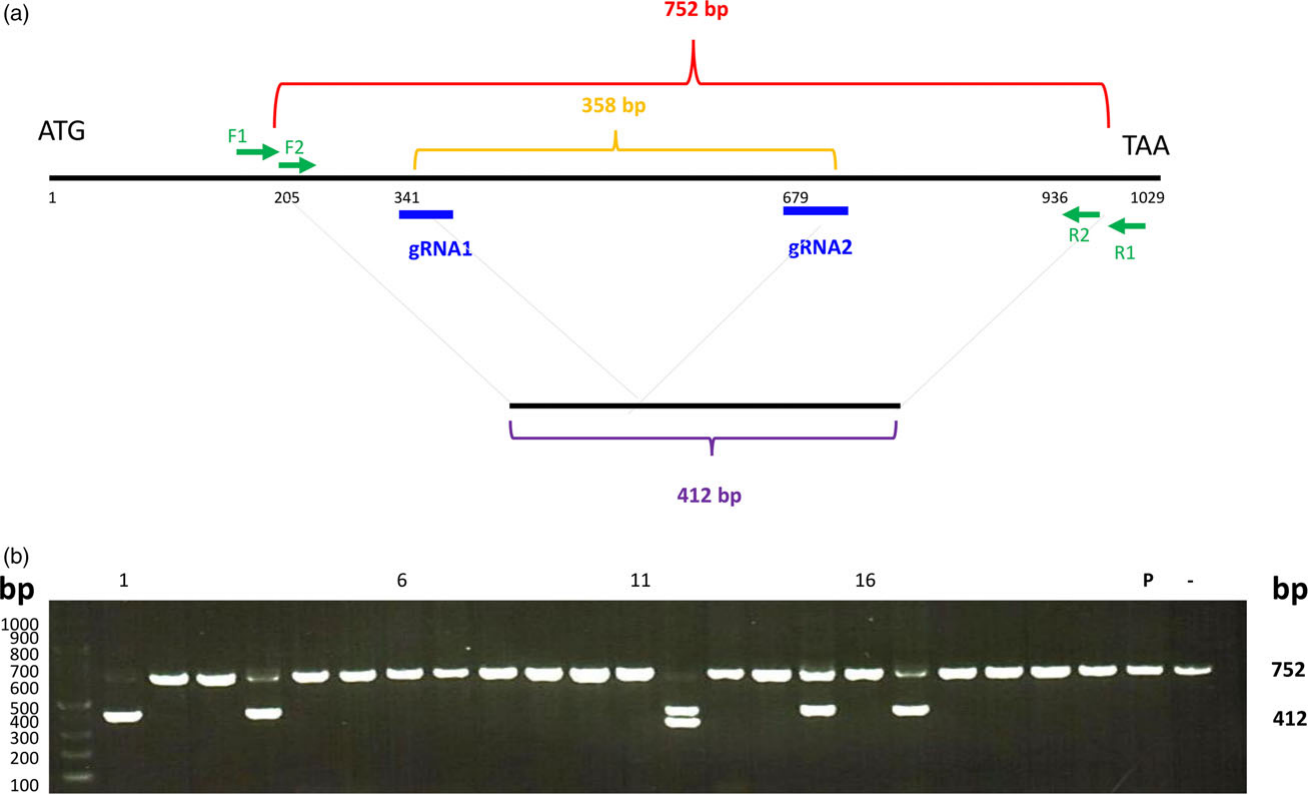
Targeted mutagenesis of the *ZmIPK* gene in protoplasts. (a) The gene structure of *ZmIPK*. Blue, gRNA positions; green, two pairs of primers for single‐cell PCR analysis; red, size of PCR product from the wild‐type gene; orange, size between two gRNAs; purple, theoretical size after precise deletion. (b) Maize protoplasts were transfected with two sgRNAs that targeted different regions of the *ZmIPK* gene. DNA from several single protoplasts was amplified by PCR 72 h after transfection. P, pooled DNA PCR product; −, no plasmid DNA transfected. Numbers above the gel lanes indicate protoplast number.

To apply this single‐protoplast protocol to other species, we investigated CRISPR‐mediated mutagenesis of the maize *ZmIPK* gene. Poaceae protoplasts are smaller than those of tobacco and *Arabidopsis*, but we could isolate individual maize protoplasts after transfection. Similar to the experiments described above, we used two sgRNA to delete a fragment of *ZmIPK*. Using a protoplast mixture, deletions could not be detected during the first round of PCR analysis, but could be observed using a second round of PCR after a restriction enzyme was used to digest the amplicons resulting from the first PCR product (Figure S8). Because of these two rounds of PCR, it is difficult to calculate target site mutagenesis efficiency using the scanned gel image. However, using a single protoplast, we could detect deletions in 23% of the protoplasts (Figure 5). Because the transfection efficiency was 55%, the calculated mutagenesis efficiency was 41%. PCR products were cloned and sequenced to confirm the deletions. The sequence data are shown in Data S3.

According to these results, single‐cell analysis is a convenient method to detect mutagenesis efficiency and to determine mutated sequences. Using pooled protoplast DNA as the template, the mutagenesis efficiency could be measured by gel image analysis. In the *ZmIPK* two sgRNA experiment (Figure 5), single‐cell analysis was more sensitive because the low amount of the PCR product containing deletions was not observed in the scanned gel image (Figure S7). To obtain the mutated sequences, the pooled DNA PCR products had to be cloned for sequencing. Using single‐cell analysis, the PCR product could be sequenced without cloning. Heterozygous PCR products were identified using bioinformatics tools. The scanned gel image analysis plus PCR product cloning/sequencing, and the single‐cell analysis which we present in this report are alternative methods to determine mutagenesis efficiency and mutated sequences. The results from these two methodologies were consistent.

### Regeneration of CRISPR‐edited plants from single protoplasts

**Figure 6:**
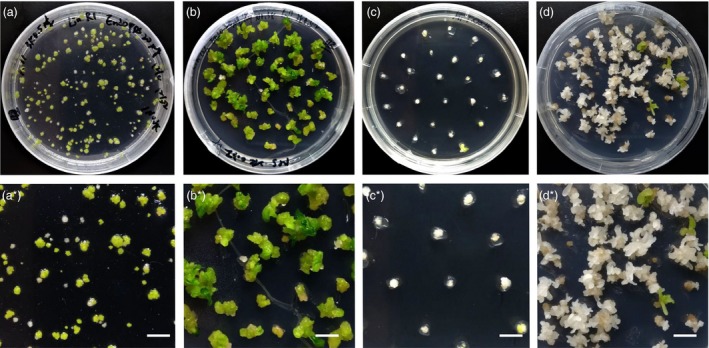
*NtPDS* mutant plants derived from CRISPR mutagenesis of transfected protoplasts. (a) Growing calli were embedded in shooting medium for 1.5 months. (a*) Magnified view of one portion of the plate shown in (a). Bar = 1 cm. (b) Green shoot clusters were subcultured in shooting medium for 3 weeks. (b*) Magnified view of one portion of the plate shown in (b). Bar = 1 cm. (c) Albino calli were subcultured in shooting medium for 3 weeks. (c*) Magnified view of one portion of the plate shown in (c). Bar = 1 cm. (d) Albino calli were subcultured in shooting medium after 7 weeks. (d*) Magnified view of one portion of the plate shown in (d). Bar = 1 cm.

**Figure 7:**
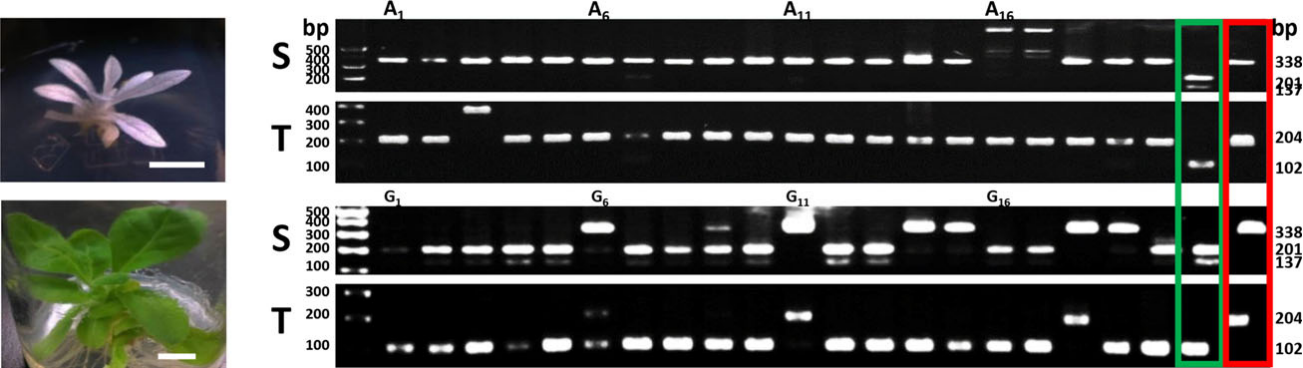
Targeted mutagenesis of *NtPDS* genes from regenerated tobacco protoplasts. Albino (A) and green (G) plantlets from regenerated protoplasts derived from 20 μg plasmid DNA treatment. Twenty plantlets of each type were analysed for *NtPDS* mutagenesis. The target sequences were amplified and validated using RFLP. S, *N. sylvestris* form; T, *N. tomentosiformis* form. Green box, wild‐type RFLP control; red box, albino mutant RFLP control.

To show that CRISPR‐mediated mutagenesis can be useful to generate genome‐edited plants, we targeted the *NtPDS* gene of tobacco. The *PDS* gene is important for chlorophyll biogenesis (Qin *et al*., 2007), and homozygous *PDS* mutants are albino. Because *N.  tabacum* contains four *PDS* genes (two each from the *N. sylvestris* and *N. tomentosiformis* progenitors), mutation of all four genes is necessary to obtain albino plants. Following transfection, protoplasts were transferred to growth medium to obtain calli. We obtained both albino and green calli (Figure 6a), which we regenerated into shoots. Green calli grew more quickly than did albino calli, but we were able to obtain shoots from both types of calli (Figure 6b–d). From three biological repeats, albino calli represented 29.1% of all regenerated calli generated using 20 μg DNA, although the percentage of albino calli was lower using less DNA (Table 3). We analysed DNA of 20 shoots from albino and green calli (Figures 7 and S16, Table 4). As expected, all albino shoots contained targeted mutations in all of the *PDS* genes. DNA sequence analysis of the few amplicons from white calli that were digested by *Bst*NI indicated that this restriction endonuclease site remained, but that other frame‐shift mutations occurred (Data S4). From the green tissue, 44.4% of the shoots contained edited S form *PDS* genes, but only 25.6% contained edited T form genes (Figures 7 and S16, Table 4). Sequencing of PCR *NtPDS* gene products from green shoots confirmed the mutations (Data S4). For all shoots (green and albino) investigated, 58.8% contained at least one mutated *NtPDS* gene.

**Table 3:** Analysis of regenerated green and albino shoots from CRISPR‐mutagenized tobacco protoplasts

Number of green and albino shoots regenerated after transfection with various amounts of Cas9‐ and sgRNA‐encoding plasmid DNA				
DNA (μg)	0	5	10	20
Green shoots (repeat 1)	113	127	62	139
Green shoots (repeat 2)	119	174	148	48
Green shoots (repeat 3)	136	129	167	123
Total green shoots	**368**	**430**	**377**	**310**
Albino shoots (repeat 1)	0	3	4	54
Albino shoots (repeat 2)	0	11	6	28
Albino shoots (repeat 3)	0	4	2	45
Total albino shoots	**0**	**18**	**12**	**127**
% albino shoots (±SE)	**0**	**4.0 ± 1.2**	**3.1 ± 1.5**	**29.1 ± 6.9**
Mutated *NtPDS* genes in green shoots (%)				
Repeat 1	0	10	20	80
Repeat 2	0	30	10	30
Repeat 3	0	30	30	40
Total mutant genes (±SE)	**0**	**23.3 ± 11.5**	**20.0 ± 10.0**	**50.0 ± 26.5**
Mutated *NtPDS* genes in all shoots (%)				
Repeat 1	0	12.1	24.8	85.6
Repeat 2	0	34.1	13.5	55.8
Repeat 3	0	32.1	30.8	56.1
Total mutant genes (±SE)	**0**	**26.1 ± 12.2**	**23.1 ± 8.8**	**65.8 ± 17.1**

Bold‐face type indicates the average±s.d. among the multiple experiments.

**Table 4:** Analysis of regenerated green and albino shoots from CRISPR‐mutagenized tobacco protoplasts which were transfected with 20 μg plasmid DNA

	Experiment 1	Experiment 2	Experiment 3	Average
Phenotypes of regenerated shoots				
Albino/Green	0.20 ± 0.07 (169/969)	0.52 ± 0.20 (205/379)	0.37 ± 0.11 (183/503)	**0.36**
Mutated *NtPDS* genes in green shoots (%)				
S	33.3 ± 2.9	46.7 ± 10.4	53.3 ± 2.9	**44.4**
T	15.0 ± 5.0	25.0 ± 10.0	36.7 ± 7.6	**25.6**
Total mutant genes (±SE)	**35.0 ± 0.0**	**46.7 ± 10.4**	**53.3 ± 2.9**	**45.0**
Mutated *NtPDS* genes in white shoots (%)				
S	100.0 ± 0.0	100.0 ± 0.0	100.0 ± 0.0	**100**
T	100.0 ± 0.0	100.0 ± 0.0	100.0 ± 0.0	**100**
Total mutant genes (±SE)	**100.0 ± 0.0**	**100.0 ± 0.0**	**100.0 ± 0.0**	**100**
Mutated *NtPDS* genes in all shoots (%)				
Total mutant genes (±SE)	**45.6 ± 23.0**	**64.9 ± 6.0**	**65.9 ± 3.3**	**58.8**
Shoots containing *Cas9* gene (%)				
Green	1.7 ± 2.9	3.3 ± 5.8	1.7 ± 2.9	**2.2**
Albino	10.0 ± 13.2	15.0 ± 8.7	26.7 ± 15.3	**17.2**

Bold‐face type indicates the average±s.d. among the multiple experiments.

The percentage of shoots with the albino phenotype was DNA dosage dependent. Ten green shoot clusters for each repetition (three repeats) for each DNA amount were subjected to RFLP and sequencing analysis (Table 3, Figure S17, Data S5). No albino calli were observed without the use of Cas9/sgRNA‐encoding DNA, and only ~4% of the shoot clusters obtained using 5 and 10 μg DNA/reaction were albino (Table 3). The reaction using 20 μg DNA/reaction resulted in the highest percentage of albino shoots.

We further investigated whether the regenerated shoots contained the *Cas9* gene. Thirty‐one of 180 albino shoots (17.2%) contained *Cas9*. Only four of the 180 green regenerants (2.2%) contained *Cas9*. Thus, we could obtain regenerated shoots from *PDS*‐edited plants that lack *Cas9* (Table 4).

## Discussion

### Improvement of protoplast isolation

CRISPR/Cas9 technology is a powerful tool for plant breeding and research. CRISPR technology is still evolving, including the use of novel Cas9 nucleases, rules for the design of gRNAs and algorithms to predict target and ‘off‐target’ sequences. The performance of these new systems and sgRNA target efficiency still relies on empirical results. However, stable transformation to evaluate a construct is time‐consuming and costly, with at least one report claiming that in maize >90 candidate target sites must be screened to find a suitable sgRNAs (Zhu *et al*., 2016). Therefore, a CRISPR platform with a rapid and efficient evaluation protocol is needed. A protoplast transient transfection system with high transfection efficiency fulfils this need. The bottleneck of isolating high‐quality protoplasts from different species has hindered this application.

For rice protoplast isolation, we made two modifications to previous methods (Chen *et al*., 2006; Zhang *et al*., 2011) to improve yield and reduce costs. First, instead of cutting the seedlings into cross section pieces, we cut the rice seedlings parallel to the veins. Early attempts to use random or cross section cutting resulted in fewer and lower quality protoplasts, likely because there are proportionally more cell walls in the same‐sized cross‐sectional area. Longitudinal cuts generated more damage to cells along the cut edge but allowed easier access of the maceration enzymes to the cells, resulting in more protoplasts released during digestion (Figure 1d,e). When seedlings were cut in cross section, more cells remained within the leaf sheath after digestion. We substituted the less expensive Cellulase R10 for the more expensive Cellulase RS (Zhang *et al*., 2011). We could apply this protocol to other Poaceae species, including wheat, bamboo, millet and corn. We believe this convenient method will benefit Poaceae crop research.

The Tape Sandwich method can be used to isolate protoplasts from Brassicaceae species [*Arabidopsis* (Wu *et al*., 2009), broccoli, *Cleome*, and rapeseed (Figure S1)] and from other family, such as Euphorbiaceae (Poinsettia, Pitzschke and Persak, 2012). However, this protocol could not be applied to tomato leaves because the epidermal cells could easily be pulled off by tape.

Although tomato mesophyll protoplasts can be isolated by leaf cutting (Niedz *et al*., 1985), this is often not convenient. Suspension cell lines are an alternative material for tomato protoplast isolation. These lines can be maintained in a controlled environment without variations caused by temperature or light (Nagata *et al*., 1992). The tobacco suspension cell line BY‐2 has been used for several purposes, such as subcellular protein localization, BiFC vector validation (Lee *et al*., 2008) and CRISPR gene editing (Mercx *et al*., 2016). In addition to a ‘Micro‐Tom’ tomato cell line, we also established a line from a different variety (CL5915; World Vegetable Center, Tainan, Taiwan) following this protocol. Other advantages of suspension cells include the ease of transgenic material containment and the ability to maintain good manufacturing practices for production of pharmacological proteins or antibodies, which can be secreted to the extracellular medium for easy purification (Mercx *et al*., 2016).

### Validation of CRISPR/Cas9‐mediated targeting mutagenesis in plant protoplasts

#### Bamboo

Bamboo is an economically and ecologically important vegetable and forest plant in Asia (Ma *et al*., 2015). Bamboo grows quickly and is a sustainable and environmentally friendly crop. Bamboo protoplasts have been isolated from suspension cells and leaves (Hisamoto and Kobayashi, 2010; Huang *et al*., 1989, 1990; Yeh *et al*., 2011). Protoplasts derived from *in vitro* propagated shoots have been transfected for subcellular protein localization studies (Yeh *et al*., 2011). In this report, the protoplast yield was increased using longitudinal cutting from *in vitro* material and immature leaves of greenhouse‐grown plants. We demonstrated that the bamboo genome can be mutated by the CRISPR/Cas9 system.

#### Millet

Millet is an important feed and food crop in arid regions (Pan *et al*., 2015). Millet is a C4‐photosynthesis plant. Because the genome size of millet is smaller than that of other grass crops (490 M), it has become a model plant for C4 photosynthesis (Doust *et al*., 2009). Because transformation of millet is inefficient (~5.5%; Wang *et al*., 2011), it is difficult to use a traditional transformation strategy to test sgRNAs for genome editing. Protoplasts can be used to screen for suitable sgRNA and Cas9 constructs for use in stable transformation. Xiang *et al*. (2004) developed a millet protoplast isolation protocol. Millet protoplasts have been used for protoplast transfection and for BiFC investigation (Liu *et al*., 2016). Using protoplast transfection, we demonstrate that the millet genome can be mutated using the CRISPR/Cas9 system.

#### Rice

Since the first report of CRISPR/Cas9‐mediated genome editing in rice, there have been more than 30 articles published about rice genome editing using CRISPR/Cas9 (e.g. Feng *et al*., 2013; Mao *et al*., 2013; Miao *et al*., 2013; Shan *et al*., 2013; Xie and Yang, 2013). Compared with other crop plants, CRISPR technology in rice is mature. Modifications reported include single sgRNA knockouts and deletion of a large DNA fragment (>200 kb; Zhou *et al*., 2014). DNA‐free genome editing has also been reported (Woo *et al*., 2015). CRISPR/Cas9 has been used in rice breeding to improve phenotypes to change leaf morphology (Ikeda *et al*., 2016), grain number, panicle architecture, grain size and plant architecture (Li *et al*., 2016a). CRISPR/Cas9 has also been used to induce male sterility (Li *et al*., 2016b), herbicide‐tolerance (Sun *et al*., 2016) and pathogen resistance (Wang *et al*., 2016). Numerous studies used protoplasts to confirm the editing efficiency of the sgRNA and Cas9 protein (Jiang *et al*., 2013; Li *et al*., 2016b; Lowder *et al*., 2015; Shan *et al*., 2013, 2014; Wang *et al*., 2016; Woo *et al*., 2015; Xie and Yang, 2013; Xie *et al*., 2015). These articles indicate that a convenient and high transient transfection protoplast protocol is useful to determine the efficiency of designed gRNAs. We improved rice protoplast isolation and showed that these protoplasts can be used for transient transfection. Our convenient rice protoplast isolation protocol will be useful to reduce the time to evaluate the efficiency of nucleases and sgRNAs.

#### Maize

Protoplasts isolated from some maize genotypes can be regenerated into plants (Rhodes *et al*., 1988; Sheen, 2001). There are several maize CRISPR/Cas9 studies that used protoplasts (e.g. Feng *et al*., 2016; Liang *et al*., 2014; Svitashev *et al*., 2015; Xing *et al*., 2014; Zhu *et al*., 2016). In this report, we targeted the *IPK* gene with RFLP validation in protoplasts. Using two sgRNAs, we could generate a large (300 bp) deletion within this gene.

### OsU3 and OsU6 application in dicots

Most current CRISPR/Cas9 studies have focused on developing only monocot or dicot vectors (Jiang *et al*., 2013). In our study, we used the rice promoters OsU3 and OsU6 to express sgRNAs in a vector containing *Cas9* and *sgRNA* genes. The results indicate that monocot promoters can be used efficiently in both monocot and dicot protoplasts.

#### Broccoli

Broccoli plants can be regenerated from mesophyll‐derived protoplasts (Robertson and Earle, 1986). These protoplasts can be transfected using a PEG‐mediated method (Nugent *et al*., 2006). We used the Tape Sandwich method to establish a broccoli protoplast transfection system using cotyledons as starting material. Lawrenson *et al*. (2015) established a broccoli CRISPR/Cas9 mutagenesis protocol. We used their sgRNA sequence in this study. In a broccoli *GA4a* CRISPR/Cas9 stable transformation study, 80% mutation frequency was achieved using the *Arabidopsis* U6 promoter to drive the same sgRNA (Lawrenson *et al*., 2015). We conclude that our high mutagenesis results from the chosen target gene and the high efficiency of protoplast isolation and transfection. The sgRNA mutation efficiency has been positively correlated with GC content, and the *GA4a* gene has a high GC ratio (65%; Ren *et al*., 2014). This speculation has to be proven by further sgRNA investigations.

#### Rapeseed

Rapeseed is cultivated mainly for its oil‐rich seed, the third largest source of vegetable oil in the world (http://apps.fas.usda.gov/psdonline/circulars/oilseeds.pdf). Kartha *et al*. (1974) described a rapeseed protoplast isolation protocol, and plantlets could be regenerated from these protoplasts. A rapeseed protoplast transient transfection system has also been established (Wang *et al*., 2015a). Our results indicate that the rapeseed protoplast genome can be mutated by CRISPR/Cas9.

#### Solanaceae

Tobacco is one of the first crops in which CRISPR/Cas9 genome editing was demonstrated (Jiang *et al*., 2013; Li *et al*., 2013; Nekrasov *et al*., 2013). There are many articles demonstrating that the tomato genome can be edited by CRISPR/Cas9 (Brooks *et al*., 2014; Cermak *et al*., 2015). In previous studies, the sgRNA was driven by an *Arabidopsis* U6 promoter. We showed that the same vectors used in monocots (pCAMBIA1300‐OsU3(AarI)‐Cas9 vectors) can be used in Solanaceae (tobacco and tomato), even though the promotors are derived from rice.

### Application of single‐cell analysis to genome editing

Numerous studies have demonstrated the utility of protoplasts to evaluate the efficiency of various CRISPR systems to mutate specific target genes (Feng *et al*., 2013; Li *et al*., 2013; Shan *et al*., 2013; Xie and Yang, 2013). Populations of protoplasts are transfected with DNA encoding Cas9 (or other editing proteins) and genes encoding sgRNAs. Alternatively, Cas9 protein/sgRNA complexes can be transfected into protoplasts (Woo *et al*., 2015). Total DNA from the protoplast population is isolated and assayed for mutagenesis frequency using RFLP or other methods of analysis, such as the T7E1 surveyor assay (Woo *et al*., 2015). When the mutation frequency is high, it is relatively easy to detect mutated molecules after electrophoresis through agarose gels. However, as we have shown above with the *IPK* gene of maize, the frequency of CRISPR‐mediated mutagenesis can be low, requiring a second round of PCR to amplify sequences from the small amount of nondigested DNA extracted from the gel.

Single‐cell (protoplast) analysis is a sensitive and convenient method to evaluate the efficiency of various sgRNAs for CRISPR‐mediated mutagenesis (Figure S18). Because only two copies of a given gene exist in a diploid cell (or four copies in an amphidiploid such as *N. tabacum*), edited copies of the gene will represent 50% of the gene molecules (for heterozygous cells) or 100% of the gene molecules (for homozygous or biallelic cells). PCR products of these genes can directly be sequenced without the need for prior cloning. Because of the small amount of DNA in a single protoplast, a gene of interest cannot be amplified by one step PCR directly, even if the protocol is increased to 50 cycles. Target sequences can be amplified in a second round of PCR (use 1 μL of the first step PCR product as a template). It is important to use nested primers for the second round of PCR. If the same primers are used for both PCR steps, only a smear of products is obtained. In a previous protocol, transfected protoplasts had to be cultured in W5 solution (Wu *et al*., 2009), then transferred to liquid callus medium for further regeneration. Our new single‐protoplast analysis protocol allows faster analysis using much less plant material.

### Homozygous transgene‐free mutant plants can be obtained from protoplasts in the first generation

One of the advantages of using protoplasts is that DNA‐free genome editing can be conducted using preassembled RNP complexes consisting of Cas9 protein and sgRNAs. These complexes were introduced directly into protoplasts using a polyethylene glycol‐based method (Woo *et al*., 2015) to generate mutagenized lettuce. RNP complexes can also be delivered to calli by particle bombardment (Liang *et al*., 2017). Generation of homozygous or biallelic mutants lacking transgenes encoding genome‐editing reagents in the first generation is important for those plant species that cannot easily be crossed to eliminate transgenes. These include vegetatively propagated species, such as potato and banana, and species with long generation times, such as woody trees.

In this report, we show that we can generate transgene‐free genome‐edited plants by transfecting protoplasts with DNA encoding sgRNAs and Cas9 protein (Figure S19). Most regenerated *N. tabacum* plants containing edited *PDS* genes lacked the *Cas9*‐encoding transgene DNA (Table 4). Homozygous transgene‐free mutant plants could be regenerated in the T1 generation. Similar mutagenesis results were reported for wheat (Zhang *et al*., 2016) and lettuce (Woo *et al*., 2015). Compared to RNP complexes, DNA encoding the genome‐editing reagents is less expensive and more convenient to use. One advantage of RNP complexes is their higher target mutation efficiency (Woo *et al*., 2015). If the efficiency of a given sgRNA were high, we recommend using DNA encoding genome‐editing reagents. As we have shown, protoplast transfection can be highly efficient and up to 60% of mutagenized regenerated tobacco plants contained at least one mutated *PDS* gene (Tables 3 and 4). In hexaploid wheat, only 0.6% (67/10448) of the plants transformed by CRISPR constructs were homozygous mutants (Zhang *et al*., 2016). We have shown that for allotetraploid tobacco, ~ 30% of the plants regenerated from mutagenized protoplasts were homozygous/tetra‐allelic mutants. In theory, we could obtain 154 transgene‐free albino mutants from one transfection {[(169 + 205 + 183)/3] × (1–0.172)}, a number much higher than can be obtained by particle bombardment (Liang *et al*., 2017; Zhang *et al*., 2016). Thus, CRISPR‐based mutagenesis of protoplasts can be a useful technology for polyploid crops, especially horticultural crops that are propagated via asexual methods. Our studies suggest that this new technology can reduce breeding time significantly.

Protoplast regeneration remains a bottleneck for this platform. Especially for monocot species, high regeneration efficiency protocols are not available (Liang *et al*., 2017; Zhang *et al*., 2016). Improvements in protoplast regeneration protocols will be important for genome editing and the delivery of high editing efficiency reagents (RNP and donor DNA) to obtain transgene‐free crops.

## Materials and methods

### Plant materials

Millet (*Setaria italica*), corn (*Zea mays*), rice (*Oryza sativa*), broccoli (*Brassica oleracea*), and rapeseed (*B*. *napus*) plants were grown on filter paper with drip irrigation in an environment‐controlled chamber with a long photoperiod (16‐h light/8‐h dark) at 25 °C. Two‐week‐old plants were used for protoplast isolation. Bamboo (*Bambusa oldhamii*) shoots were incubated in Murashige and Skoog (MS) salts medium (Sigma‐Aldrich, St. Louis, MO) supplemented with 0.1 mg/L TDZ (Lin *et al*., 2007). *Arabidopsis* seeds were grown in soil using a 16‐h light/8‐h dark cycle at 26 °C. *Cleome spinosa*,* C. monophilla*, and *C. gynadra* plants were grown in a greenhouse. Tobacco BY‐2 cells were grown in MS salts supplemented with 1 mg/L thiamine‐HCl, 370 mg/L KH_2_PO_4_, 30 g/L sucrose, and 2 mg/L 2,4‐dichlorophenoxyacetic acid (2,4‐D), pH 5.7 (Lee *et al*., 2008). A tomato Micro‐Tom cell line was subcultured in MS medium supplemented with 1 mg/L 2,4‐D, pH 5.7. Detailed information on this cell line is presented in Figure 1.

### Protoplast isolation and PEG‐mediated protoplast transfection

Protoplast isolation and transfection were performed following Wu *et al*. (2009) for Brassicaceae, Zhang *et al*. (2011) for Poaceae, and Lee *et al*. (2008) for Solanaceae, with modifications. Details are described in Table S1 for Poaceae, Table S2 for Brassicaceae, and Table S3 for Solanaceae. Following transfection, 1.5 mL W5 solution were added, the protoplasts were transferred into six‐well plates (precoated with 1% BSA solution), and cultured in the dark at room temperature for 48 h.

### Plasmid constructions

The primers OsU3‐*Hind*III‐f and OsU3‐*Hind*III‐r were used to amplify the gRNA cassette (OsU3 promoter, *Aar*I restriction site and gRNA scaffold) from the plasmid pU3‐gRNA which was ligated into *Hin*dIII digested pCAMBIA1300 to generate pCAMBIA1300‐OsU3(*Aar*I) (Figure S2). The Cas9 cassette fragment [35S promoter, human codon‐optimized *SpCas9* (*hSpCas9*) gene and terminator] was obtained from 35S‐Cas9‐SK by digestion with *Sal*I and *Eco*RI and ligated into *Sal*I/*Eco*RI digested pCAMBIA1300‐OsU3(*Aar*I) to generate pCAMBIA1300‐OsU3(*Aar*I)‐Cas9 (Accession no.: KX400856). The same strategy was used to generate pCAMBIA1300‐OsU6(*Aar*I)‐Cas9 (Accession no.: KX388151). Detailed information is found in Supplemental Material and Methods.

### Mutation validation in protoplasts

Genomic DNA was extracted from pooled protoplasts using a Mini GenoPlus Genomic DNA Extraction Kit (Viogene, New Taipei City, Taiwan). To amplify the genomic region targeted by the sgRNA, two pairs of primers were designed. The restriction enzyme and primer sequence information are shown in the Supplemental Figures. PCR conditions are 94 °C for 5 min, 35 cycles of 94 °C for 30 s, annealing (55–63 °C; detailed information is shown in the Supplemental Figures) for 30 s, polymerization at 72 °C for 30 s, followed by 72 °C for 3 min. The PCR product was digested by the appropriate restriction enzyme and the products subjected to electrophoresis. Electrophoresis gel images were analysed by ImageJ (Schneider *et al*., 2012). The intensity of the undigested band was divided by the total intensity of all bands to generate the raw mutation frequency. The raw mutation frequency was divided by the transfection efficiency to obtain the final mutation frequency. Secondary PCR products were cloned into the T&A™ vector (FYC002‐20P; Yeastern Biotech Co. LTD, New Taipei City, Taiwan). Colonies harbouring the edited DNA were screened by a PCR/restriction enzyme assay, and the insert DNA was sequenced. VirD2‐NLS‐mRFP plasmid DNA (Lee *et al*., 2008) was transfected into protoplasts by PEG‐mediated methods as a positive control to determine transfection efficiency.

### Single‐protoplast isolation and target mutagenesis validation

Transfected protoplasts were centrifuged (300× *g*, 3 min., 25 °C, Eppendorf Centrifuge 5804R; Eppendorf, Hamburg, Germany) and transferred to liquid callus medium [1/2 MS medium supplemented with 0.4 m mannitol, 30 g/L sucrose, 1 mg/L 1‐naphthaleneacetic acid (NAA) and 0.3 mg/L kinetin]. Protoplast concentration was measured using a hemocytometer and adjusted to 1 cell/μL. A single protoplast was isolated using a pipette (SelectPette, Genomics, New Taipei City, Taiwan), transferred to a slide, and the condition checked by microscopy (Olympus, Tokyo, Japan). A single protoplast was transferred to 20 μL PCR mixture containing the first pair of primers for the initial DNA amplification. One microliter of the PCR product was used as a template for a second round of PCR using a second pair of primers. Amplicons resulting from the second PCR were digested by the appropriate restriction endonuclease and analysed by electrophoresis to validate mutation of the target sequence. A schematic for single‐cell analysis is shown in Figure S18.

### Tobacco protoplast regeneration

We used a protocol modified from Liao (1990, Figure S19). Transfected protoplasts were incubated in a 5‐cm‐diameter Petri dish containing liquid callus medium. After 2‐ to 3‐week incubation in the dark, the protoplasts proliferated and formed dust‐like calli. The calli were embedded in solidified callus medium in a 9‐cm‐diameter petri dish and incubated at 25 °C for 3–4 weeks in the dark. Calli larger than 3 mm were embedded in shoot medium for shoot induction. After 1 month at 25 °C in the light (light/dark: 16/8 h; 3000 lux), shoot clusters containing leaves >5 mm were transferred to fresh shoot medium for 2–3 weeks for further shoot proliferation. Shoot clusters with leaves were then transferred to solidified root medium.

## Competing interests

The authors declare that they have no competing interests.

## Funding

This work was supported by funds from the Agricultural Biotechnology Research Center, Academia Sinica of Taiwan (to CSL and MCS).

## Author contributions

CSL, LYL, SBG, and MCS conceived and designed the experiments. YZ, RZ, WW, and WJC constructed vectors. CSL, LHY, CTH, and FHW performed targeted mutagenesis and protoplast‐related experiments. CTH, CSL, and LJL conducted protoplast regeneration. CTH, QWC, FJY, CTY, LHY, and FHW analysed the data. CSL, LYL, LHY, CTH, SBG, and HCWH interpreted the data. CSL, LYL, SBG, and MCS wrote the manuscript.

## Supporting information


**Figure S1** Protoplast transfection of various species.
**Figure S2** Construction of pCAMBIA1300‐OsU3‐Cas9.
**Figure S3** Targeted mutagenesis of *Bambusa oldhamii* protoplasts.
**Figure S4** The putative mutated *BoPDS* PCR products from *Bambusa oldhamii* protoplasts were cloned and validated by sequencing.
**Figure S5** Targeted mutagenesis of *Setaria italica* protoplasts.
**Figure S6** Targeted mutagenesis of *Oryza sativa* protoplasts.
**Figure S7** Targeted mutagenesis of *Zea mays* protoplasts.
**Figure S8** The PCR‐RFLP‐PCR results of *ZmIPK* mutagenesis.
**Figure S9** Targeted mutagenesis of *Arabidopsis thaliana* protoplasts using constructs carrying the OsU3 or OsU6 monocot promoter.
**Figure S10** Targeted mutagenesis of *Brassica oleracea* protoplasts using constructs carrying the OsU3 or OsU6 monocot promoter.
**Figure S11** The putative mutated *BolGA4a* PCR products from *Brassica oleracea* protoplasts were cloned and validated by sequencing.
**Figure S12** Targeted mutagenesis of *B. napus* protoplasts using constructs carrying the OsU3 or OsU6 monocot promoter.
**Figure S13** Targeted mutagenesis of *Nicotiana tabacum* protoplasts.
**Figure S14** Targeted mutagenesis of *Solanum lycopersicum* protoplasts.
**Figure S15** Effect of incubation times on *NtPDS* target mutagenesis analysed in single protoplasts in Experiments 2 and 3.
**Figure S16** Targeted mutagenesis of *NtPDS* in tobacco protoplast regenerants of Experiment 1.
**Figure S17** Effect of plasmid dosage on *NtPDS* mutagenesis in tobacco protoplast regenerants.
**Figure S18** Schematic representation of single‐cell isolation and validation of targeted mutagenesis.
**Figure S19** Schematic of tobacco protoplast regeneration.

**Table S1**. Protocol for protoplast isolation and PEG transformation of different Poaceae species.
**Table S2** Protocol for protoplast isolation and PEG transformation of different Brassicaceae species.
**Table S3** Protocol for protoplast isolation and PEG transformation of different Solanaceae species.

**Data S1** The *NtPDS* sequences of Figure 3 (Experiment 1) and Table 2.

**Data S2** The *NtPDS* sequences of Figure 4 (Experiment 1) and Figure S15 (Experiment 2 and 3).

**Data S3** The sequences of *Zea mays* single‐cell *ZmIPK* genes in Figure 5.

**Data S4** The *NtPDS* sequences of Figure 7 (Exp2R1) and Table 4.

**Data S5** The *NtPDS* sequences of Figure S17.
