# Supplementary material for: Application of protoplast technology to CRISPR/Cas9 mutagenesis: from single‐cell mutation detection to mutant plant regeneration
Source: Plant Biotechnol J. 2018 Jan 10;16(7):1295–310. doi: 10.1111/pbi.12870 (PMC5999315; doi:10.1111/pbi.12870)
Supplement: Supplementary file 1 — Figure S1 Protoplast transfection of various species. Figure S2 Construction of pCAMBIA1300‐OsU3‐Cas9. Figure S3 Targeted mutagenesis of Bambusa oldhamii protoplasts. Figure S4 The putative mutated BoPDS PCR products from Bambusa oldhamii protoplasts were cloned and validated by sequencing. Figure S5 Targeted mutagenesis of Setaria italica protoplasts. Figure S6 Targeted mutagenesis of Oryza sativa protoplasts. Figure S7 Targeted mutagenesis of Zea mays protoplasts. Figure S8 The PCR‐RFLP‐PCR results of ZmIPK mutagenesis. Figure S9 Targeted mutagenesis of Arabidopsis thaliana protoplasts using constructs carrying the OsU3 or OsU6 monocot promoter. Figure S10 Targeted mutagenesis of Brassica oleracea protoplasts using constructs carrying the OsU3 or OsU6 monocot promoter. Figure S11 The putative mutated BolGA4a PCR products from Brassica oleracea protoplasts were cloned and validated by sequencing. Figure S12 Targeted mutagenesis of B. napus protoplasts using constructs carrying the OsU3 or OsU6 monocot promoter. Figure S13 Targeted mutagenesis of Nicotiana tabacum protoplasts. Figure S14 Targeted mutagenesis of Solanum lycopersicum protoplasts. Figure S15 Effect of incubation times on NtPDS target mutagenesis analysed in single protoplasts in Experiments 2 and 3. Figure S16 Targeted mutagenesis of NtPDS in tobacco protoplast regenerants of Experiment 1. Figure S17 Effect of plasmid dosage on NtPDS mutagenesis in tobacco protoplast regenerants. Figure S18 Schematic representation of single‐cell isolation and validation of targeted mutagenesis. Figure S19 Schematic of tobacco protoplast regeneration. [file PBI-16-1295-s006.docx]

**
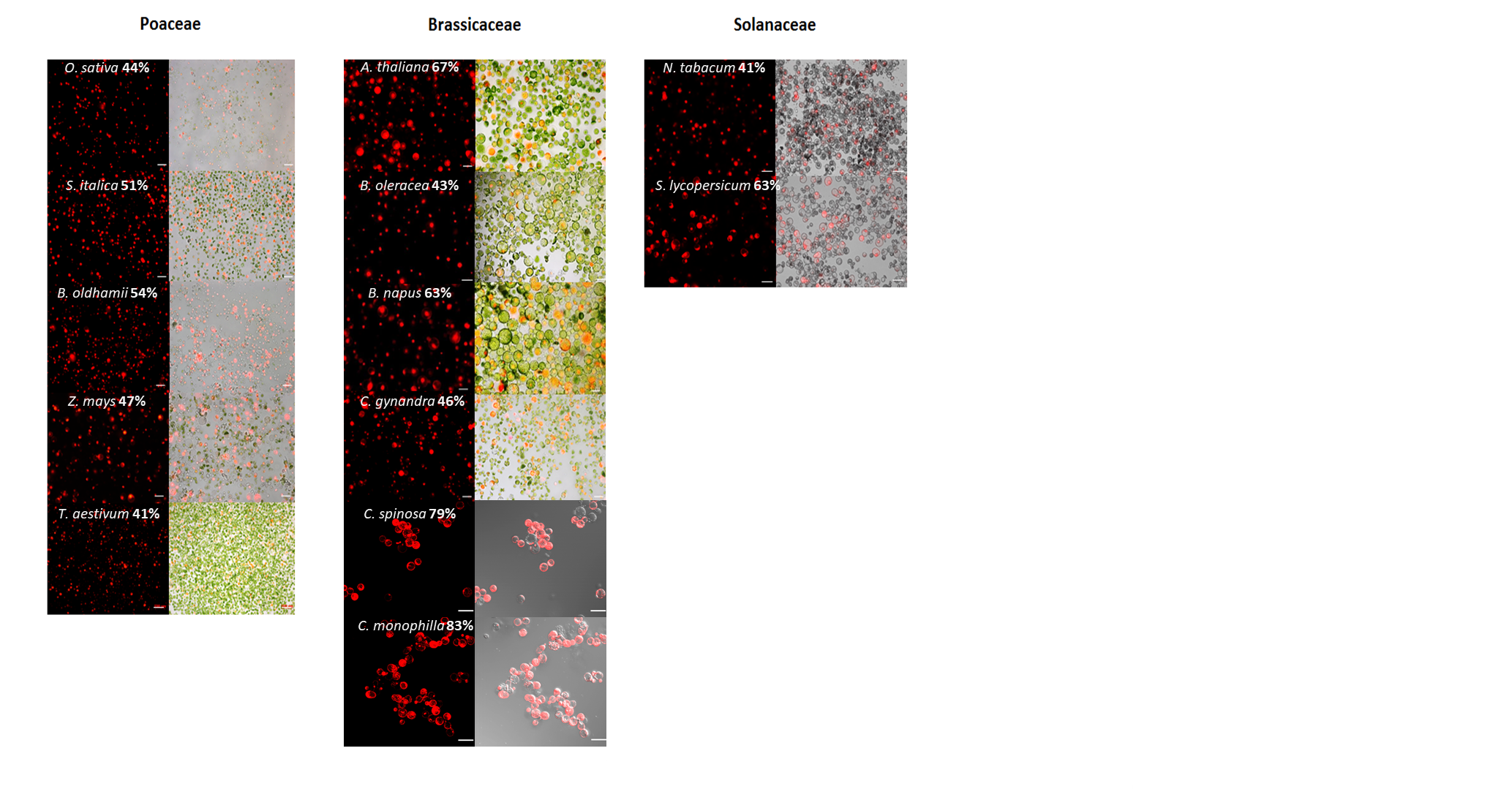
**

**Supplemental Figure 1. Protoplast transfection of various species.**

Protoplasts from 13 species were isolated following the methods described in Materials and Methods section. Twenty µg mRFP-NLS plasmid DNA was separately delivered to protoplasts from each of the 13 species using a PEG-mediated method. Number after the species name: transfection efficiency. Protoplasts were photographed after 24 hrs. For each sample: left, epifluorescence; right, overlay of epifluorescence and bright field. Bars = 50 µm.


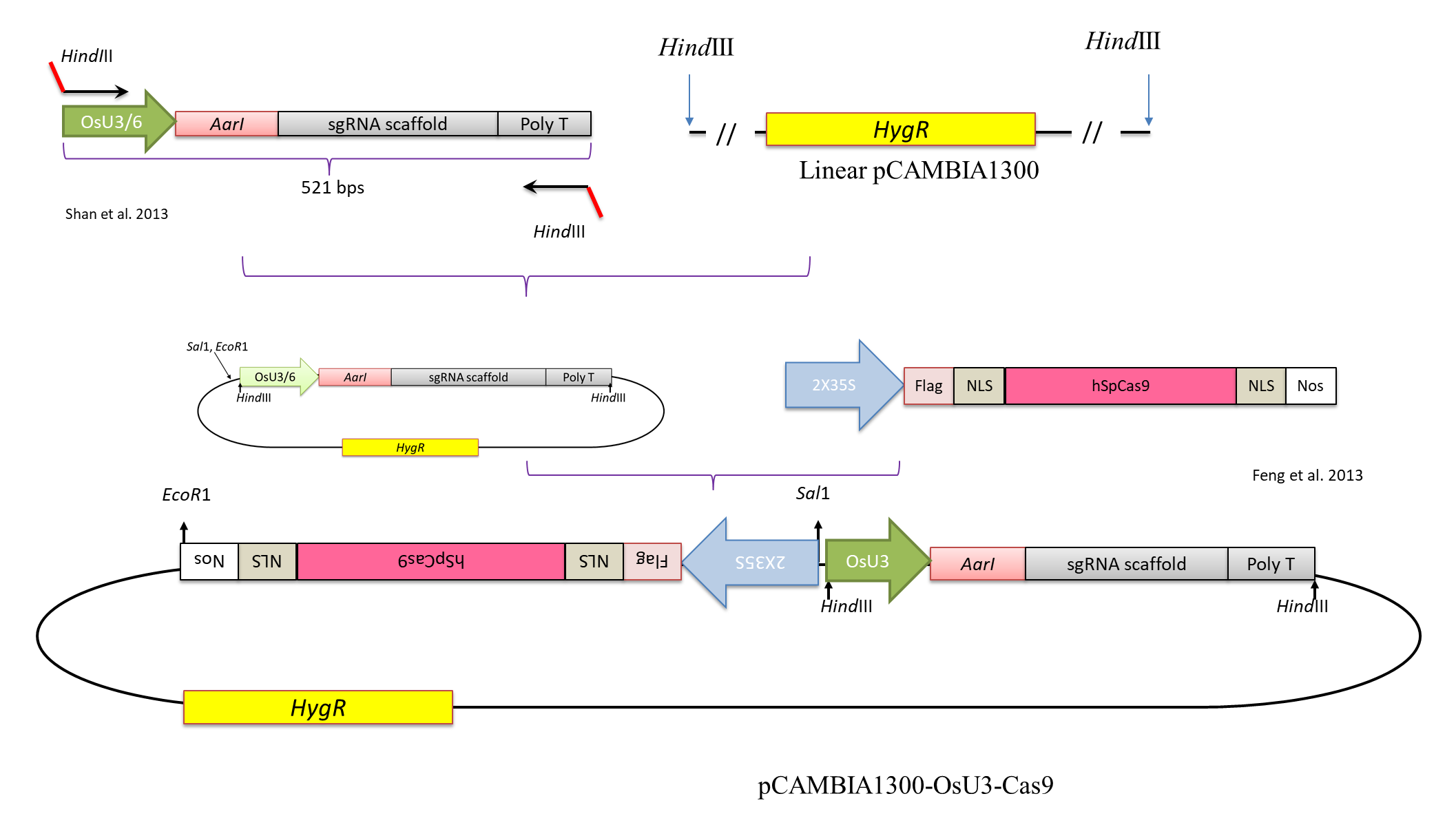


**Supplemental Figure 2. Construction of pCAMBIA1300-OsU3-Cas9.**

Any target site fragment encoding the guide RNA with suitable adaptor sequences can be added to the sgRNA scaffold by insertion into the *Aar*I site.


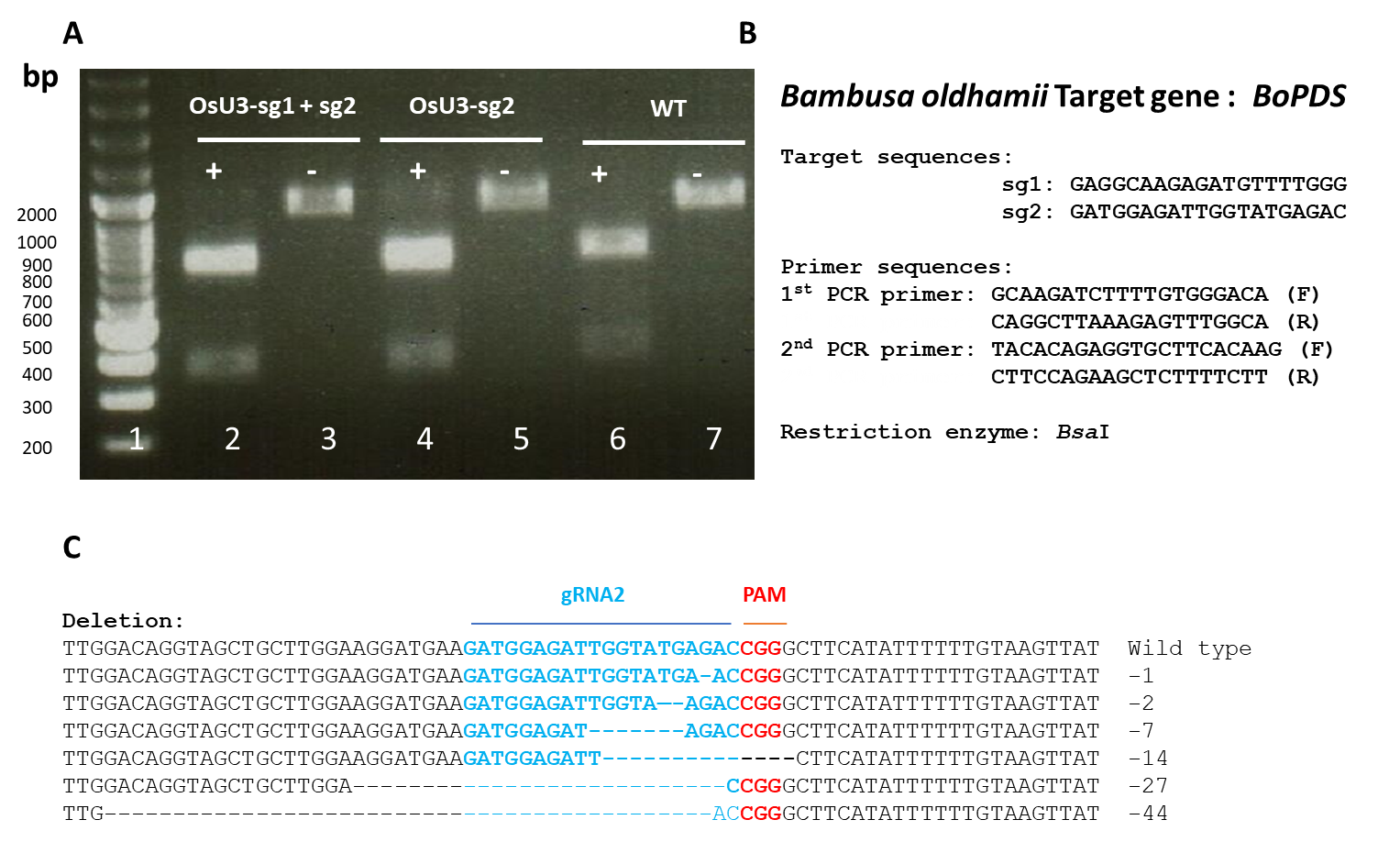


**Supplemental Figure 3. Targeted mutagenesis of *Bambusa oldhamii* protoplasts.**

1. Detection of mutations using PCR-RFLP. Lane 1, DNA marker; Lanes 2 and 3, PCR products of digested (+) and undigested (-) genomic DNA from protoplasts treated with 35S-Cas9-OsU3-*BoPDS* sgRNA1 and sgRNA2, and incubated for 48 hrs. Lanes 4 and 5, PCR products of digested (+) and undigested (-) genomic DNA from protoplasts treated with 35S-Cas9-OsU3-*BoPDS* sgRNA2, and incubated 48 hrs. Lanes 6 and 7, PCR products of digested (+) and undigested (-) genomic DNA from protoplasts of wild-type control plants.
2. Information on the target gene, including the primer sequences used to amplify the target region after transfection and the restriction enzyme used in this experiment.


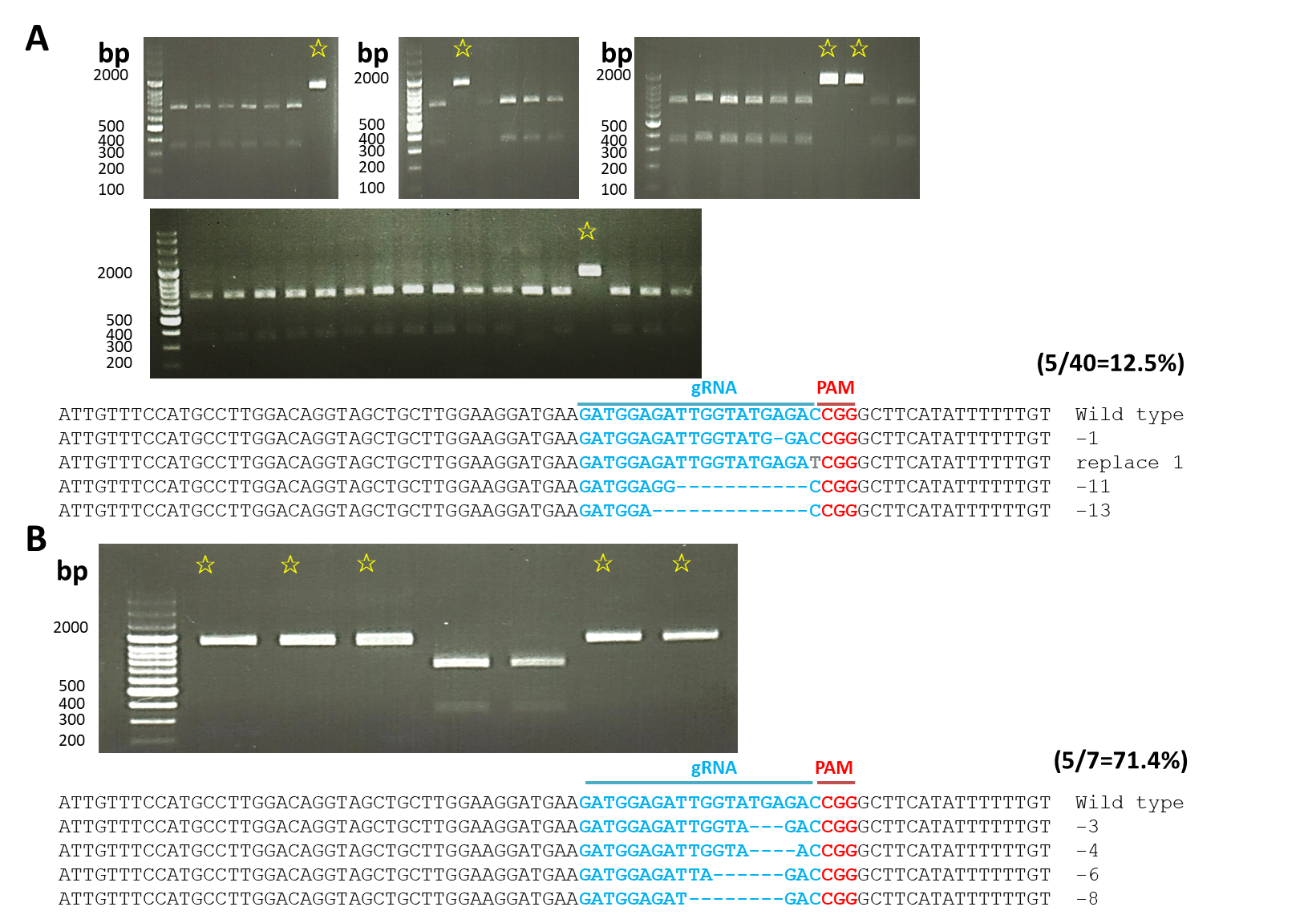


**Supplemental Figure 4.** **The putative mutated *BoPDS* PCR products from *Bambusa oldhamii* protoplasts were cloned and validated by sequencing.** DNA of *B. oldhamii* protoplasts 48 hrs after transfection with 35S-Cas9-OsU3-*BoPDS* sgRNA was isolated and subjected to PCR using primers targeting the *PDS* gene (A) or a second round of PCR (B). The products were subjected to TA cloning. Inserted DNA was amplified by PCR and sequenced by the Sanger method.

1. The PCR products (from Supplemental Figure 3A) were TA-cloned, and colony PCR products were digested by *Bsa*I. Yellow star: putative *BoPDS* mutant clones. The putative mutant clones were subjected to Sanger sequencing. The sequence of the wild-type target gene (blue) and the PAM sequence (red) are highlighted.
2. In this experiment, a second PCR step was added after PCR-RFLP (A). The mixture was subjected to restriction digestion after PCR amplification. The PCR product was TA-cloned and subjected to colony PCR. The colony PCR products were digested by *Bsa*I. Yellow star: putative mutant *PDS* clones. These clones were subjected to Sanger sequencing. The sequence of the wild-type target gene (blue) and the PAM sequence (red) are highlighted.


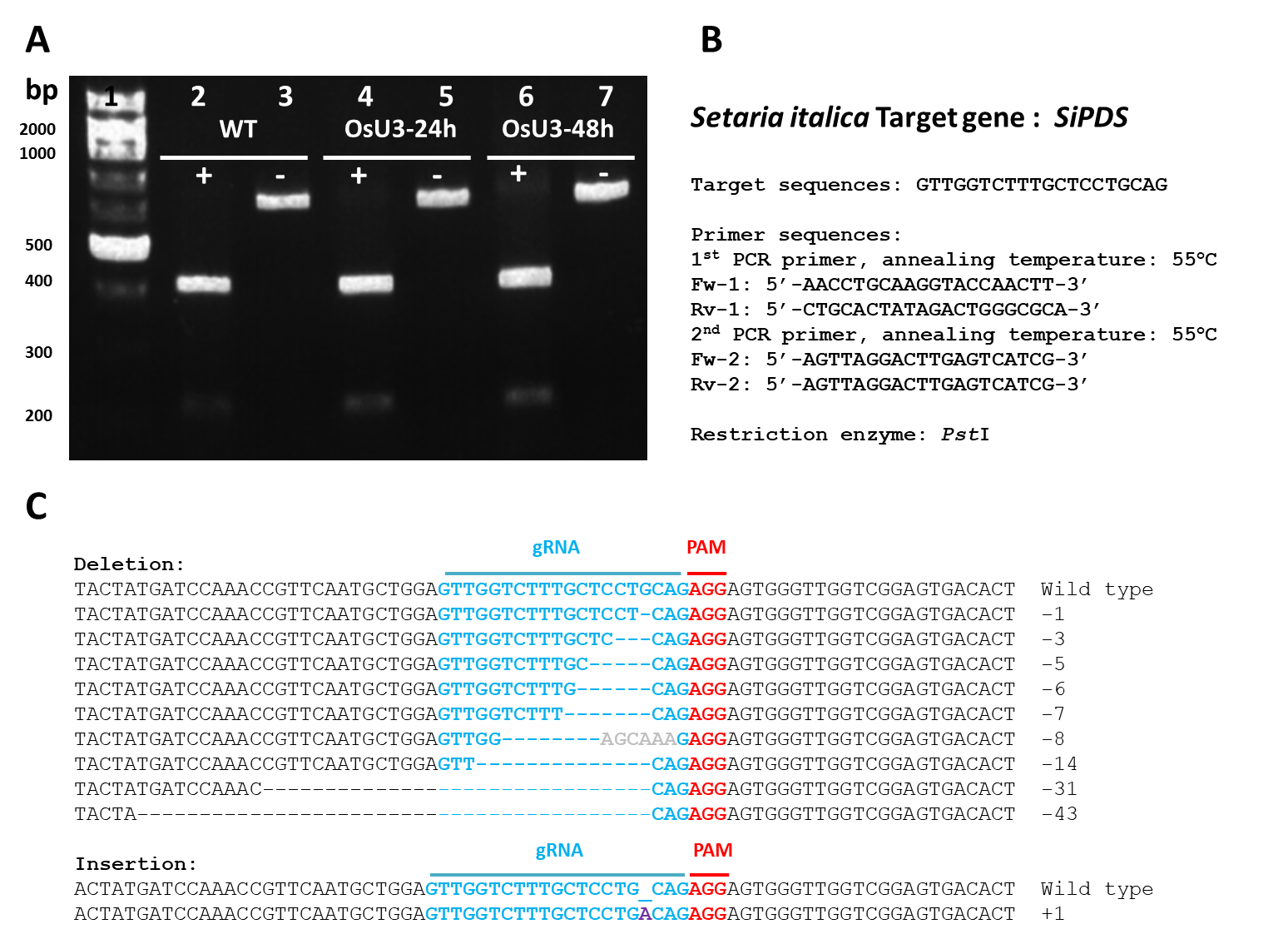


**Supplemental Figure 5.** **Targeted mutagenesis of *Setaria italica* protoplasts.**

1. Detection of mutations using PCR-RFLP. Lane 1, DNA marker; Lanes 2 and 3, PCR products of digested (+) and undigested (-) genomic DNA from protoplasts of wild-type control plant. PCR products of genomic DNA from protoplasts treated with 35S-Cas9-OsU3-*SiPDS* sgRNA incubated 24 hrs (Lanes 4 and 5) and 48 hrs (Lanes 6 and 7); -, undigested control.
2. Information on the target gene, including the primer sequences used to amplify the target region after transfection and the restriction enzyme used in this experiment.
3. Sequence-based detection of mutations induced by Cas9-OsU3-*SiPDS* sgRNA. Wild-type sequence of the target gene (blue), PAM sequence (red), and insertion sequences (purple) are highlighted. The light grey color indicates inserted DNA within the deletion region.


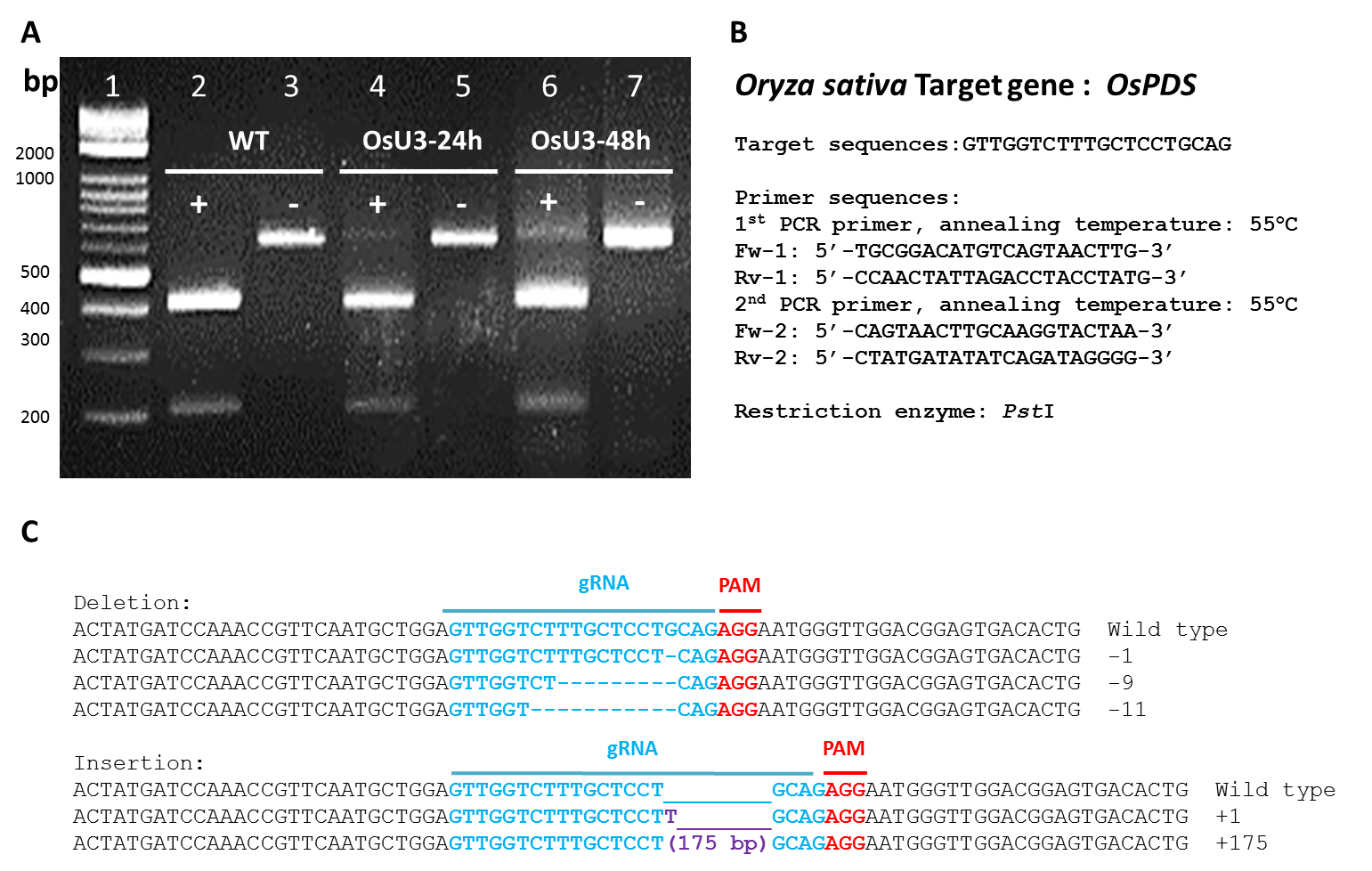


**Supplemental Figure 6.** **Targeted mutagenesis of *Oryza sativa* protoplasts.**

1. Detection of mutations using PCR-RFLP. Lane 1, DNA marker; Lanes 2 and 3, PCR products of digested (+) and undigested (-) genomic DNA from protoplasts of wild-type control. PCR products of genomic DNA from protoplasts exposed to 35S-Cas9-OsU3- *OsPDS* sgRNA incubated 24 hrs (Lanes 4 and 5) and 48 hrs (Lanes 6 and 7) -, undigested control.
2. Information on the target gene, including the primer sequences used to amplify the target region after transfection and the restriction enzyme used in this experiment.
3. Sequence-based detection of mutations induced by Cas9-OsU3-*OsPDS* sgRNA. Sequence of the wild-type target gene (blue). The protospacer-adjacent motif (PAM, red) sequence and inserted sequences (purple) are highlighted. The number in parentheses indicates the size of the insert.


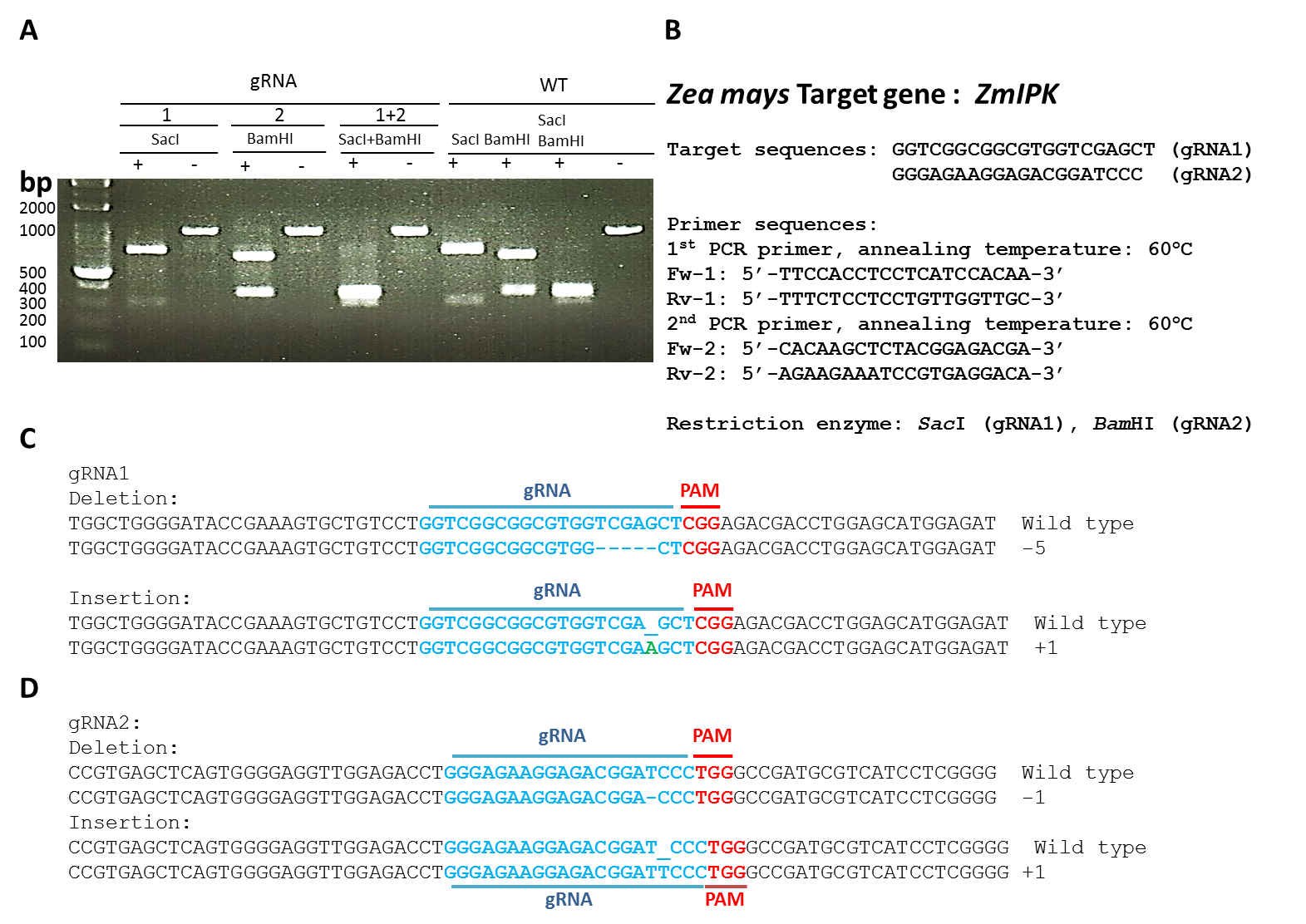


**Supplemental Figure 7. Targeted mutagenesis of *Zea mays* protoplasts.**

1. Maize protoplasts were transfected with different sgRNAs: 1, 35S-Cas9-OsU3- *ZmIPK* sgRNA1; 2, 35S-Cas9-OsU3- *ZmIPK* sgRNA2; or 1+2, co-transfected with both sgRNAs 1 and 2 that target different regions of the *IPK* gene. DNA from the protoplast pool was amplified by PCR 48 hrs after transfection. The PCR products were digested by restriction enzymes (sgRNA1, *Sac*I; sgRNA2, *Bam*HI) according to the targeted sequence.
2. Information on the target gene regions, including the primer sequences used to amplify the target regions and the restriction enzymes used in this experiment.
3. Sequence-based detection of mutations induced by Cas9-OsU3-*ZmIPK* sgRNA1. Wild-type sequence of the target gene (blue), the PAM sequence (red), and the mutated nucleotide (green) are highlighted.
4. Sequence-based detection of mutations induced by Cas9-OsU3-*ZmIPK* sgRNA2. Wild-type sequence of the target gene (blue) and the PAM sequence (red) are highlighted.


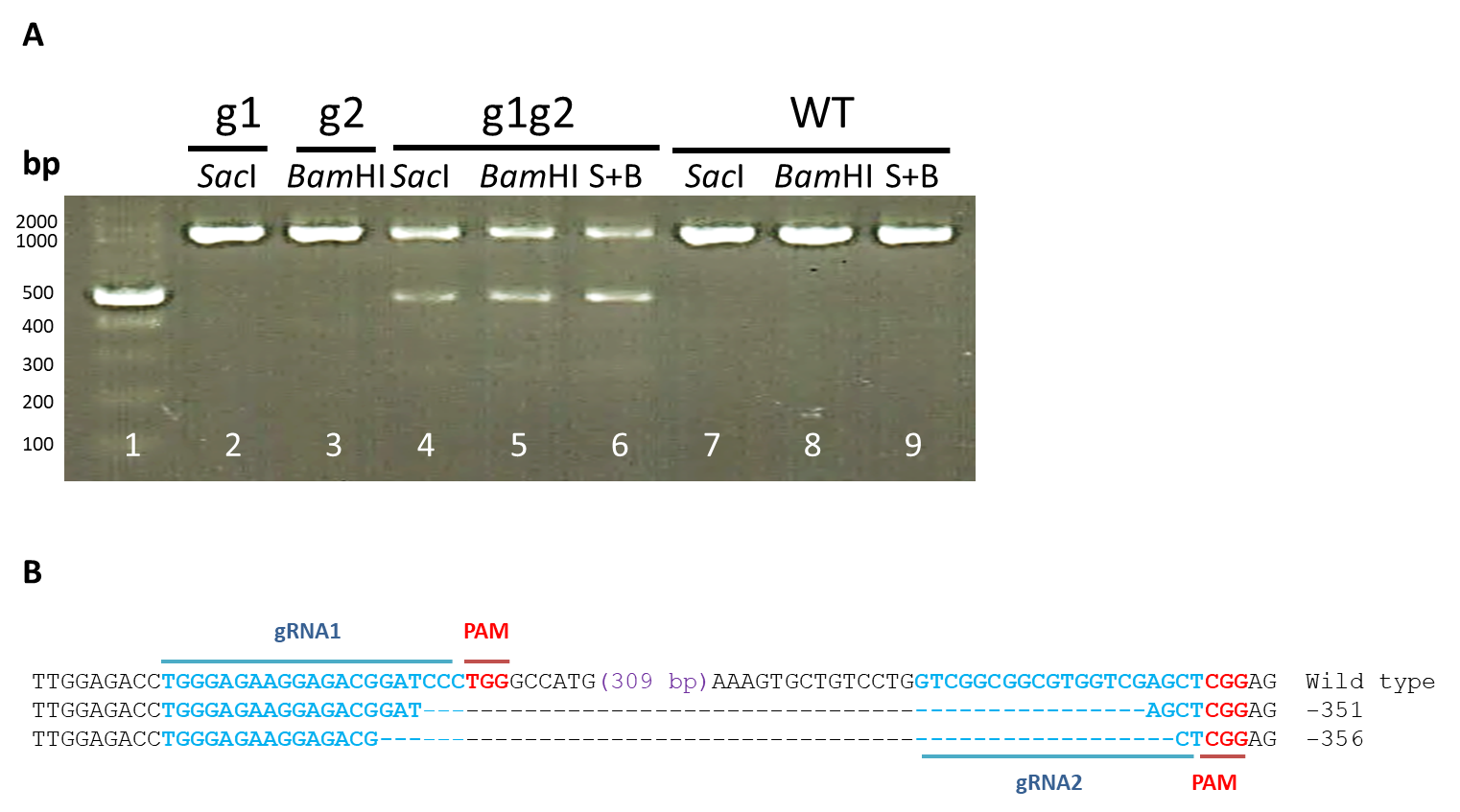


**Supplemental Figure 8.** **The PCR-RFLP-PCR results of *ZmIPK* mutagenesis.**

1. PCR-amplified and restriction enzyme-digested (PCR-RFLP) DNA from Supplemental Figure 7A was re-amplified by PCR from the restriction enzyme mixture. Lane 1, DNA markers; Lane 2, PCR-amplified DNA from protoplasts transfected with only *ZmIPK* sgRNA1 was digested with *Sac*I and subjected to a second PCR (PCR-RFLP-PCR); Lane 3, PCR-amplified DNA from protoplasts transfected with only *ZmIPK* sgRNA2 was digested with *Bam*HI and subjected to a second PCR (PCR-RFLP-PCR); Lanes 4-6, PCR-amplified DNA from protoplasts co-transfected with both *ZmIPK* sgRNA1 and sgRNA2 was digested with *Sac*I (Lane 4), *Bam*HI (Lane 5), or *Sac*I and *Bam*HI (Lane 6) and then subjected to a second round of PCR (PCR-RFLP-PCR); Lanes 7-9, PCR-amplified DNA from wild-type protoplasts was digested with *Sac*I (Lane 7), *Bam*HI (Lane 8), or *Sac*I and *Bam*HI (Lane 9) and then subjected to a second round of PCR.
2. Sequence-based detection of deletions induced by co-transfection of maize protoplasts with Cas9-OsU3-*ZmIPK* sgRNA1 and sgRNA2. Sequence of the wild-type target gene (blue) and the PAM sequence (red) are highlighted. The number in parentheses indicates the size of the deletion.


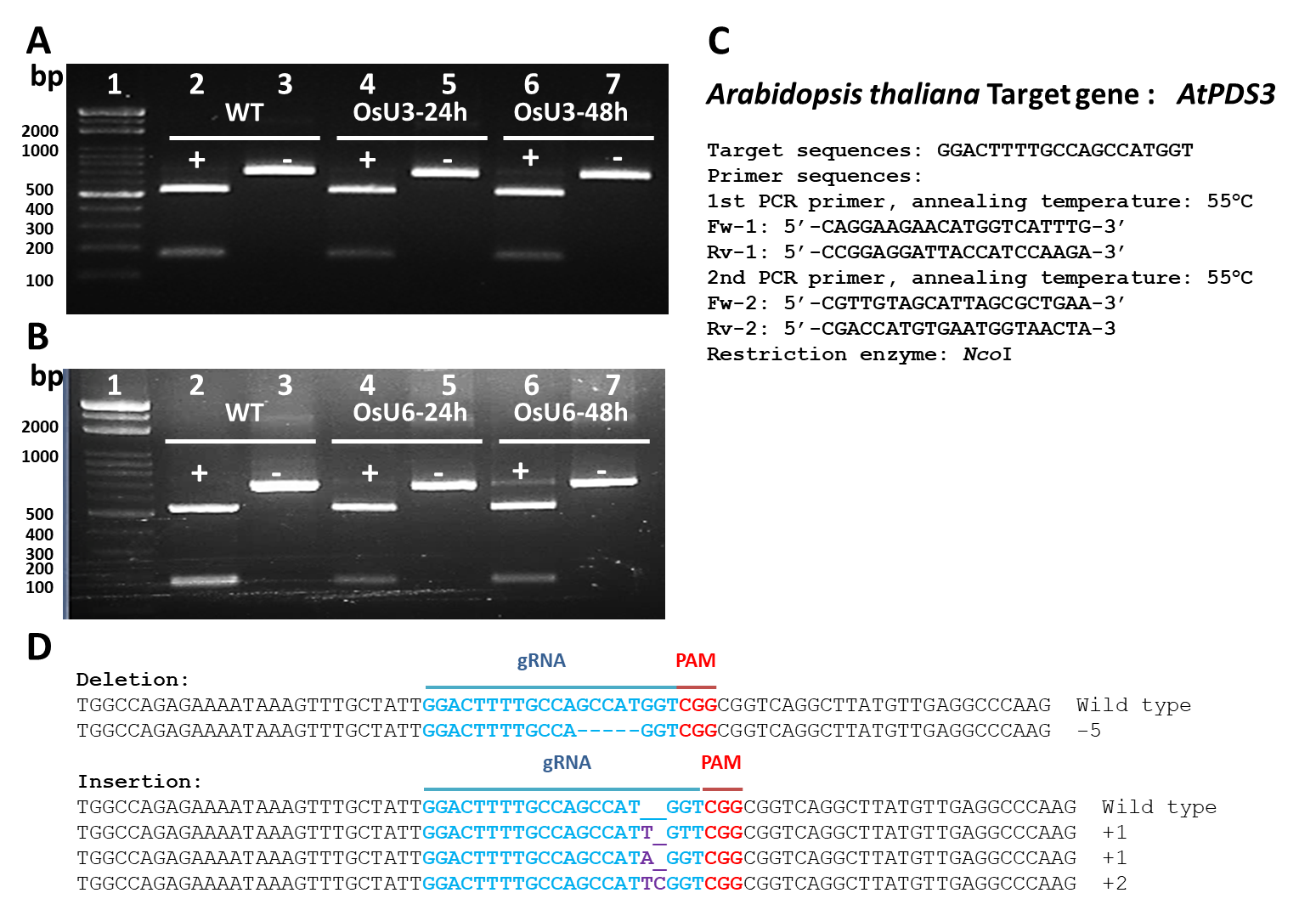


**Supplemental Figure 9. Targeted mutagenesis of *Arabidopsis thaliana* protoplasts using constructs carrying the OsU3 or OsU6 monocot promoter.**

1. Detection of mutations using PCR-RFLP. Lane 1, DNA marker; Lanes 2 and 3, PCR products of digested (+) and undigested (-) genomic DNA from protoplasts of wild-type control plants. PCR products of digested (+) and undigested (-) genomic DNA from protoplasts treated with 35S-Cas9-OsU3-*AtPDS* sgRNA after incubation for 24 hrs (Lanes 4 and 5) or 48 hrs (Lanes 6 and 7).
2. Detection of mutations using PCR-RFLP. Lane 1, DNA marker; Lanes 2 and 3, PCR products of digested (+) and undigested (-) genomic DNA from protoplasts of wild-type control plants. PCR products of digested (+) and undigested (-) genomic DNA from protoplasts treated with 35S-Cas9-OsU6-*AtPDS* sgRNA after incubation for 24 hrs (Lanes 4 and 5) or 48 hrs (Lanes 6 and 7).
3. Information on the target gene, including the primer sequences used to amplify the target region and the restriction enzyme used in this experiment.
4. Sequence-based detection of mutations induced by Cas9-OsU6-*AtPDS* sgRNA. Sequence of the wild-type target gene (blue), the PAM sequence (red), and inserted sequences (purple) are highlighted.


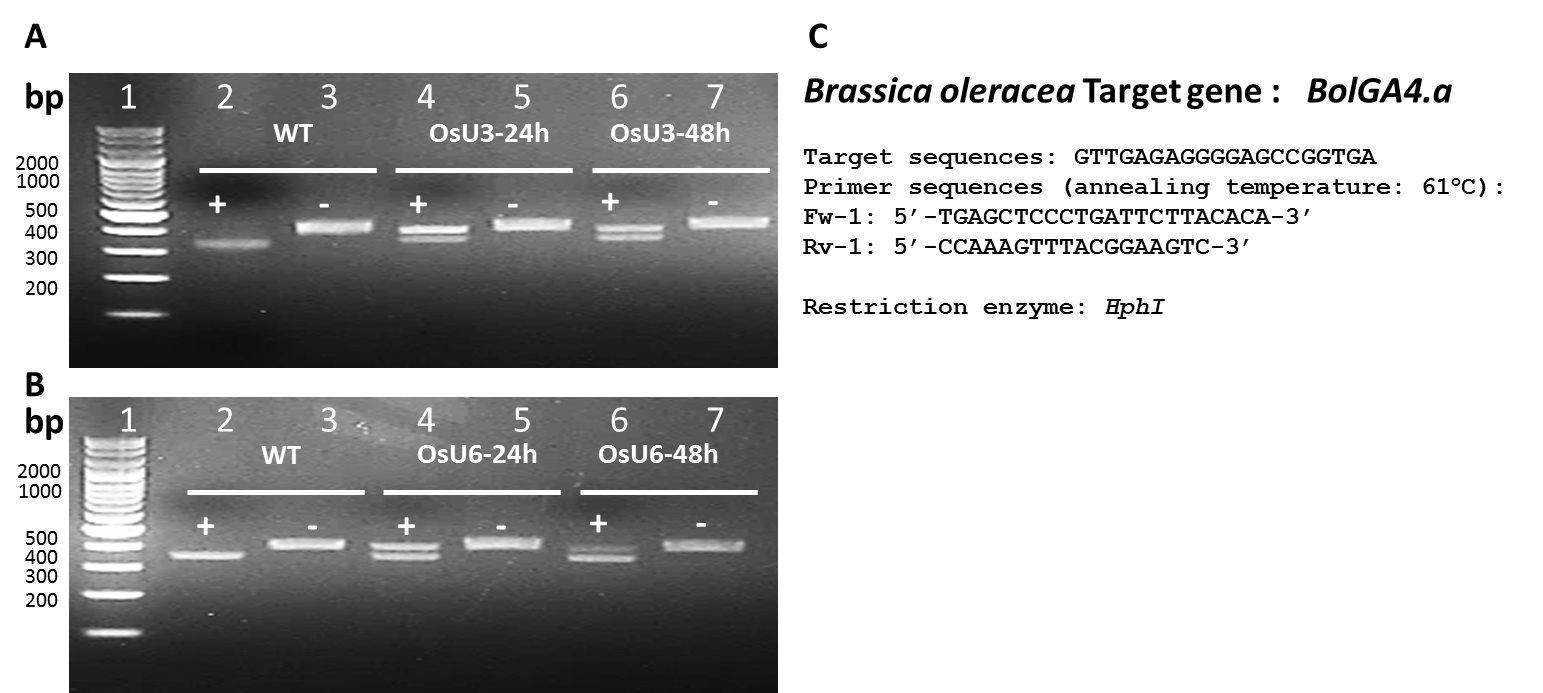


**Supplemental Figure 10. Targeted mutagenesis of *Brassica oleracea* protoplasts using constructs carrying the OsU3 or OsU6 monocot promoter.**

1. Detection of mutations using PCR-RFLP. Lane 1, DNA marker; Lanes 2 and 3, PCR products of digested (+) and undigested (-) genomic DNA from protoplasts of wild-type control plants. PCR products of digested (+) and undigested (-) genomic DNA from protoplasts treated with 35S-Cas9-OsU3- *BolGA4a* sgRNA after incubation for 24 hrs (Lanes 4 and 5) or 48 hrs (Lanes 6 and 7).
2. Detection of mutations using PCR-RFLP. Lane 1, DNA marker; Lanes 2 and 3, PCR products of digested (+) and undigested (-) genomic DNA from protoplasts of wild-type control plants. PCR products of digested (+) and undigested (-) genomic DNA from protoplasts treated with 35S-Cas9-OsU6- *BolGA4a* sgRNA after incubation for 24 hrs (Lanes 4 and 5) or 48 hrs (Lanes 6 and 7).
3. Information on the target genes, including the primer sequences which were used to amplify the target region, and the restriction enzyme which was used in this experiment.


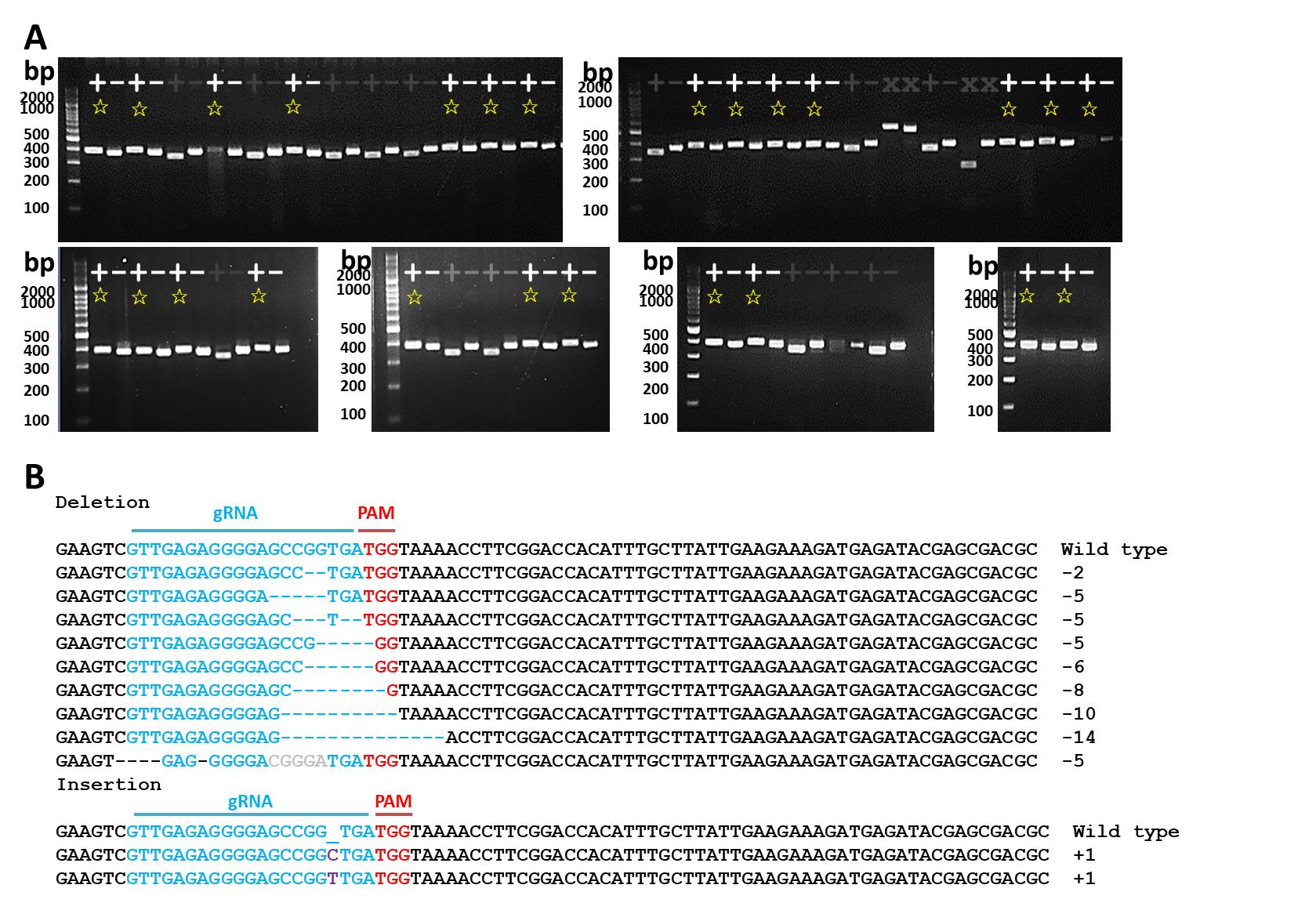


**Supplemental Figure 11.** **The putative mutated *BolGA4a* PCR products from *Brassica oleracea* protoplasts were cloned and validated by sequencing.** DNA from *B. oleracea* protoplasts transfected with 35S-Cas9-OsU3-*BolGA4a* sgRNA after 24 hrs of incubation was isolated, subjected to PCR, and T/A-cloned.

1. The colony PCR products were digested with *Hph*I. +, *Hph*I digested; -, control. Symbols colored in white, putative mutated clones; Gray, wild-type.
2. Sequence-based detection of mutations induced by Cas9-OsU3-*BolGA4a* sgRNA. Sequence of the wild-type target gene (blue), the PAM sequence (red), and inserted sequences (purple) are highlighted.


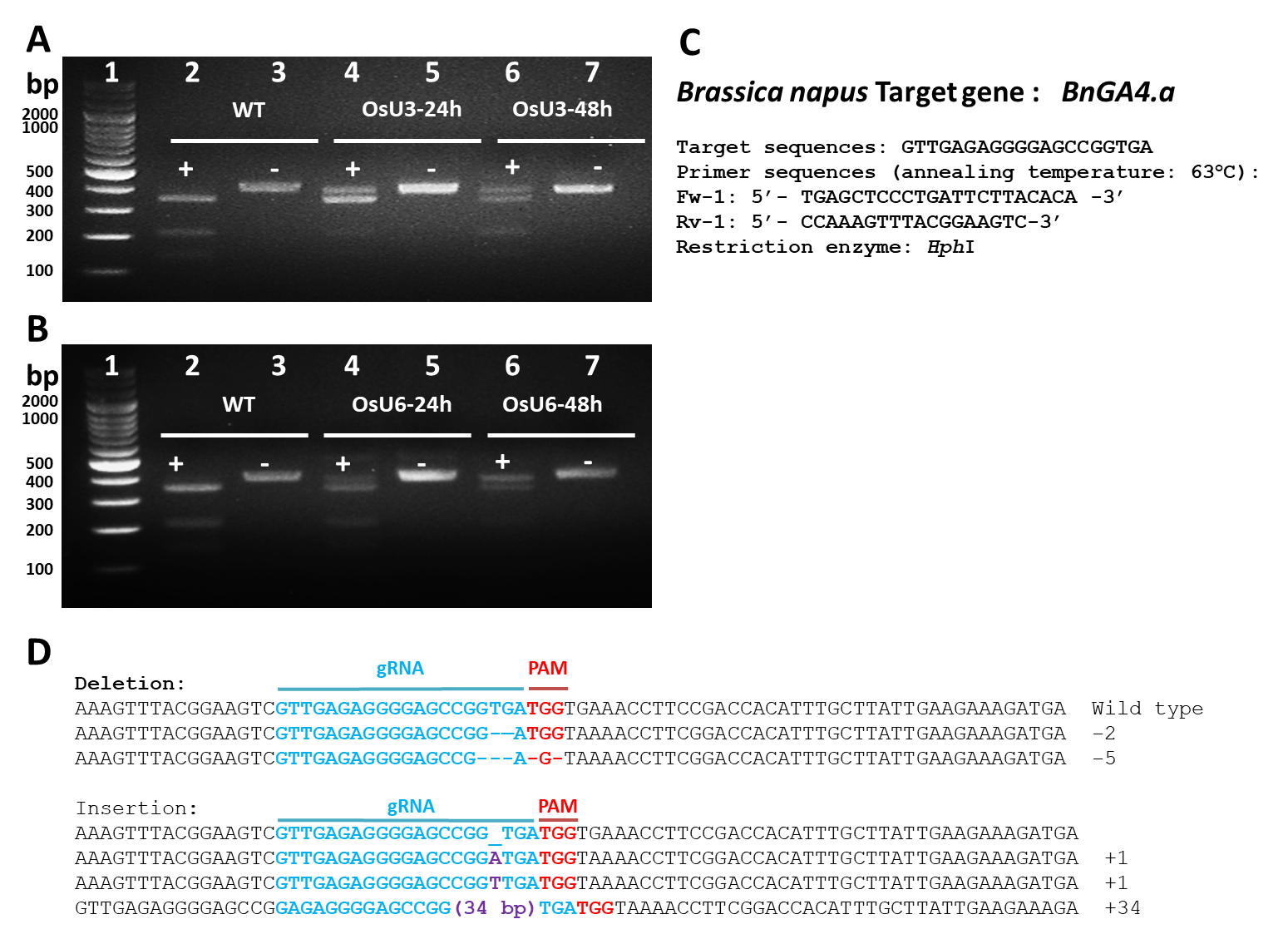


**Supplemental Figure 12. Targeted mutagenesis of *B. napus* protoplasts using constructs carrying the OsU3 or OsU6 monocot promoter.**

1. Detection of mutations using PCR-RFLP. Lane 1, DNA marker; Lanes 2 and 3, PCR products of digested (+) and undigested (-) genomic DNA from protoplasts of wild-type control plants. PCR products of digested (+) and undigested (-) genomic DNA from of protoplasts treated with 35S-Cas9-OsU3- *BnGA4a* sgRNA after incubation for 24 hrs (Lanes 4 and 5) or 48 hrs (Lanes 6 and 7).
2. Detection of mutations using PCR-RFLP. Lane 1, DNA marker; Lanes 2 and 3, PCR products of digested (+) and undigested (-) genomic DNA from protoplasts of wild-type control plants. PCR products of digested (+) and undigested (-) genomic DNA from of protoplasts treated with 35S-Cas9-OsU6- *BnGA4a* sgRNA after incubation for 24 hrs (Lanes 4 and 5) or 48 hrs (Lanes 6 and 7).
3. Information on the target gene, including the primer sequences used to amplify the target region and the restriction enzyme used in this experiment.
4. Sequence-based detection of mutations induced by Cas9-OsU3-*BnGA4a* sgRNA. Sequence of the wild-type target gene (blue), the PAM sequence (red), and inserted sequences (purple) are highlighted.

**
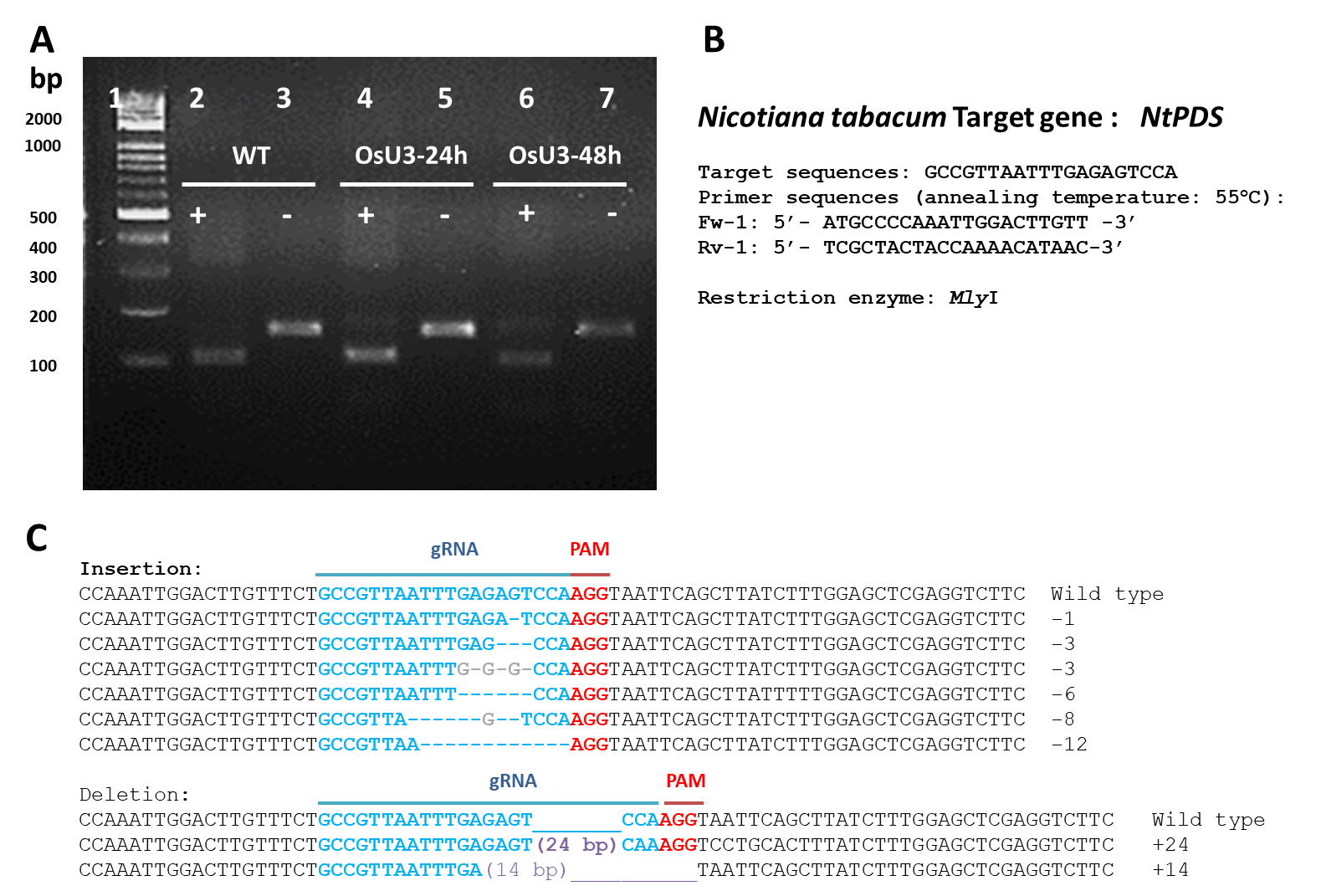
Supplemental Figure 13.** **Targeted mutagenesis of *Nicotiana tabacum* protoplasts.**

1. Detection of mutations using PCR-RFLP. Lane 1, DNA marker; Lanes 2 and 3, PCR products of digested (+) and undigested (-) genomic DNA from protoplasts of wild-type control plants. PCR products of digested (+) and undigested (-) genomic DNA from of protoplasts treated with 35S-Cas9-OsU3- *NtPDS* sgRNA after incubation for 24 hrs (Lanes 4 and 5) or 48 hrs (Lanes 6 and 7).
2. Information on the target gene, including the primer sequences used to amplify the target region and the restriction enzyme used in this experiment.
3. Sequence-based detection of mutations induced by Cas9-OsU3-*NtPDS* sgRNA.

Sequence of the wild-type target gene (blue), the PAM sequence (red), and inserted sequences (purple) are highlighted. The numbers in parentheses indicate the size of the insertion.


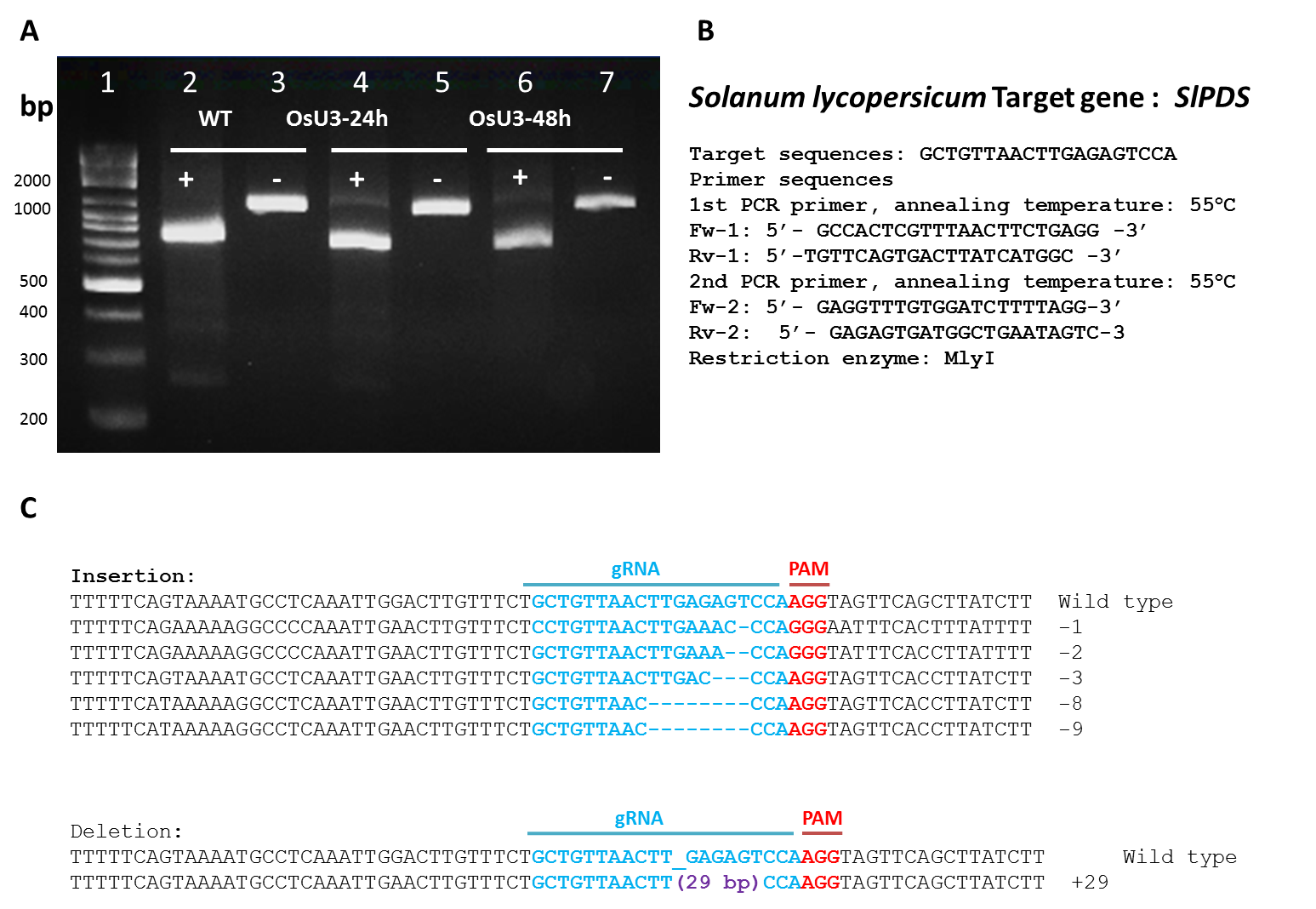


**Supplemental Figure 14.** **Targeted mutagenesis of *Solanum lycopersicum* protoplasts.**

1. Detection of mutations using PCR-RFLP. Lane 1, DNA marker; Lanes 2 and 3, PCR products of digested (+) and undigested (-) genomic DNA from protoplasts of wild-type control plants. PCR products of digested (+) and undigested (-) genomic DNA from of protoplasts treated with 35S-Cas9-OsU3- *SlPDS* sgRNA after incubation for 24 hrs (Lanes 4 and 5) or 48 hrs (Lanes 6 and 7).
2. Information on the target gene, including the primer sequences used to amplify the target region and the restriction enzyme used in this experiment.
3. Sequence-based detection of mutations induced by Cas9-OsU3-*SlPDS* sgRNA. Sequence of the wild-type target gene (blue), the PAM sequence (red) and inserted sequences (purple) are highlighted. The number in parentheses indicates the size of the insertion.


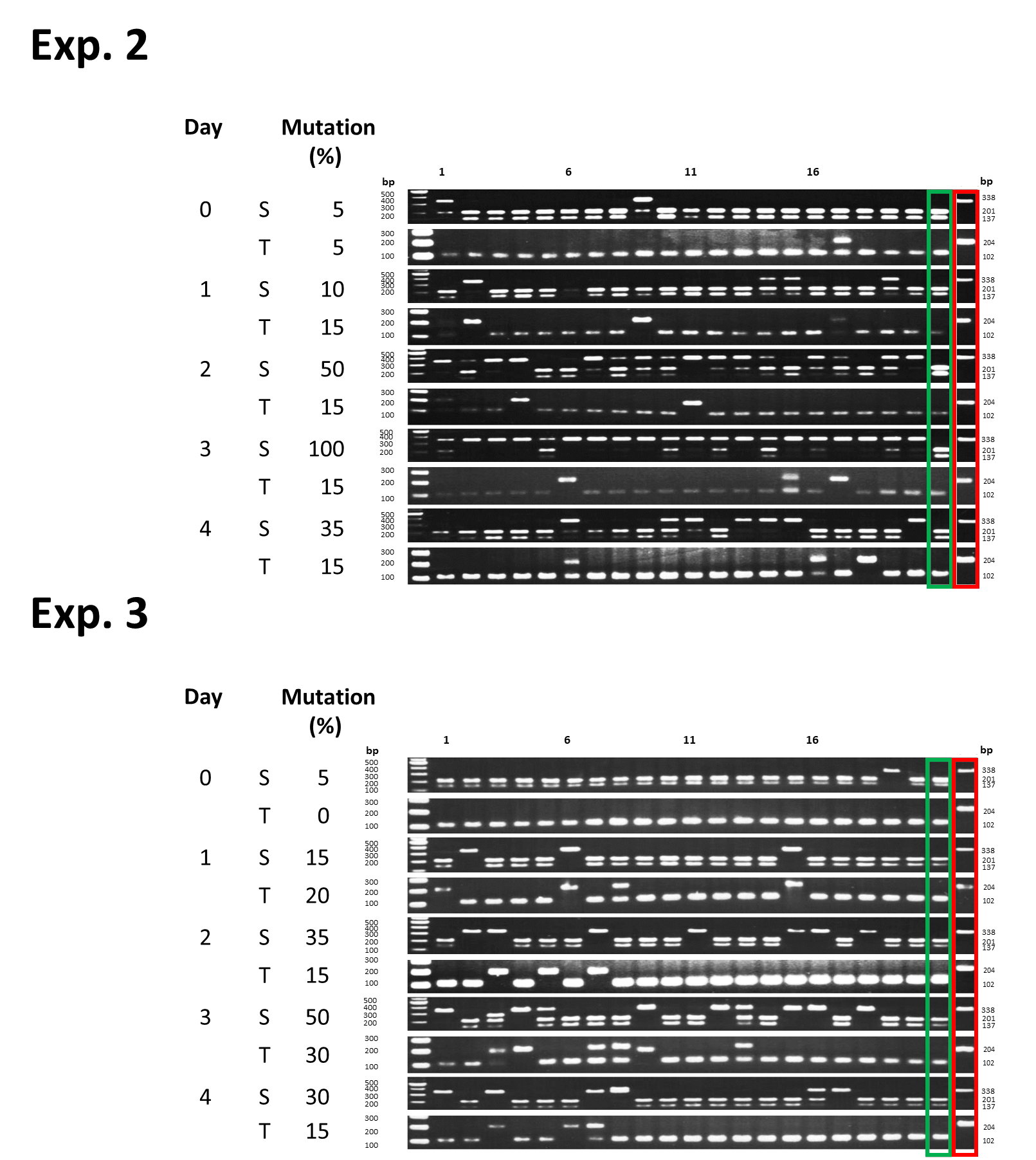


**Supplemental Figure 15. Effect of incubation times on *NtPDS* target mutagenesis analyzed in single protoplasts in Experiments 2 and 3.**

Tobacco protoplasts were transfected with 20 µg plasmid DNA containing the expression cassette of *NtPDS* sgRNA and SaCas9 (Kaya et al., 2016) and incubated for various number of days. Target mutations were analyzed by RFLP. S, *N. sylvestris* form; T, *N. tomentosiformis* form. Green frame, wild-type RFLP control; red frame, albino mutant RFLP control.


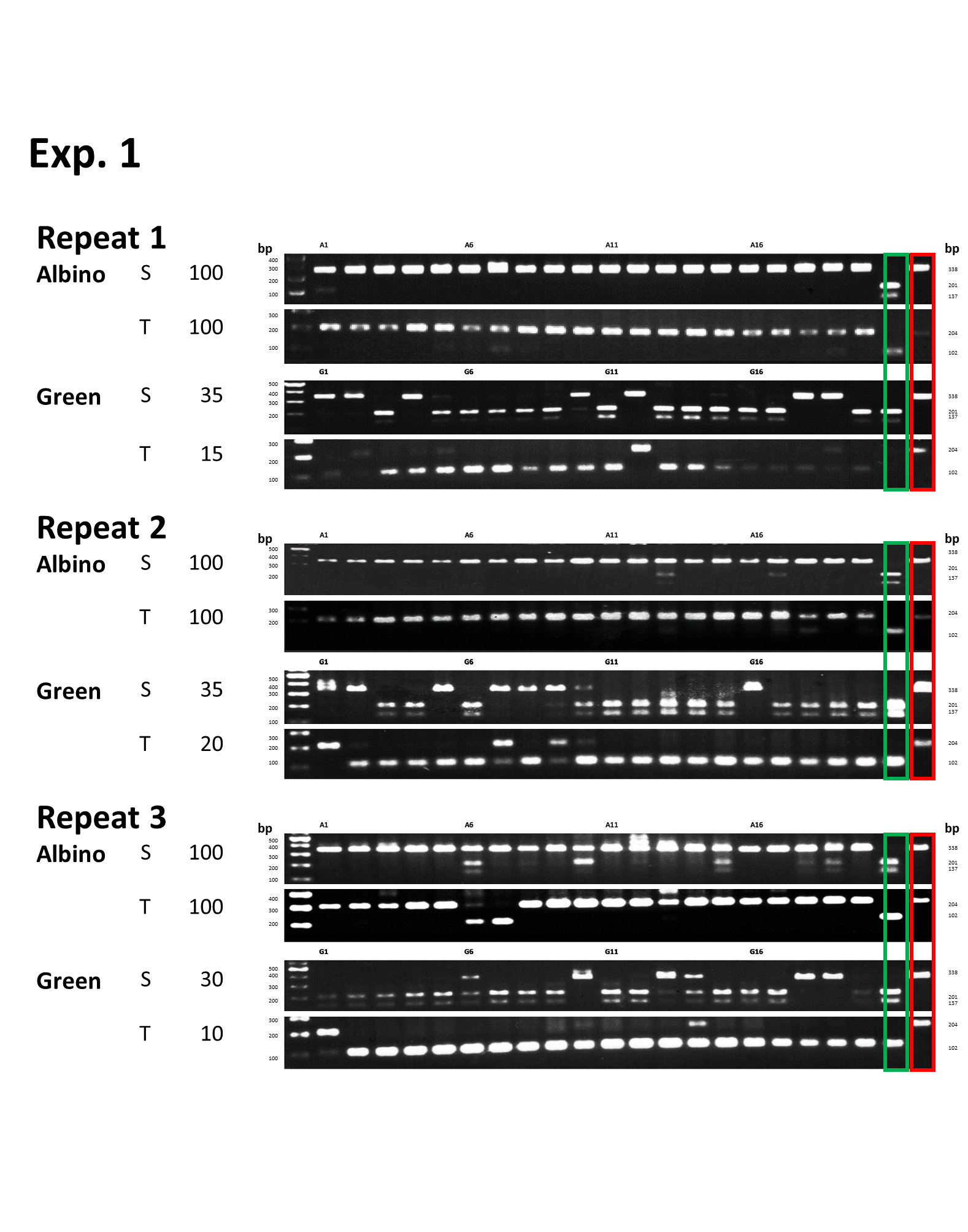


**Supplemental Figure 16. Targeted mutagenesis of *NtPDS* in tobacco protoplast regenerants of Experiment 1.**

Protoplast regenerants derived from 20 µg plasmid DNA treatments were divided into albino (A) and green (G) plantlets. Twenty plantlets in each type were analyzed for targeted mutagenesis. The target sequences were amplified and validated using RFLP. S, *N. sylvestris* form; T, *N. tomentosiformis* form. Green frame, wild-type RFLP control; red frame, albino mutant RFLP control.


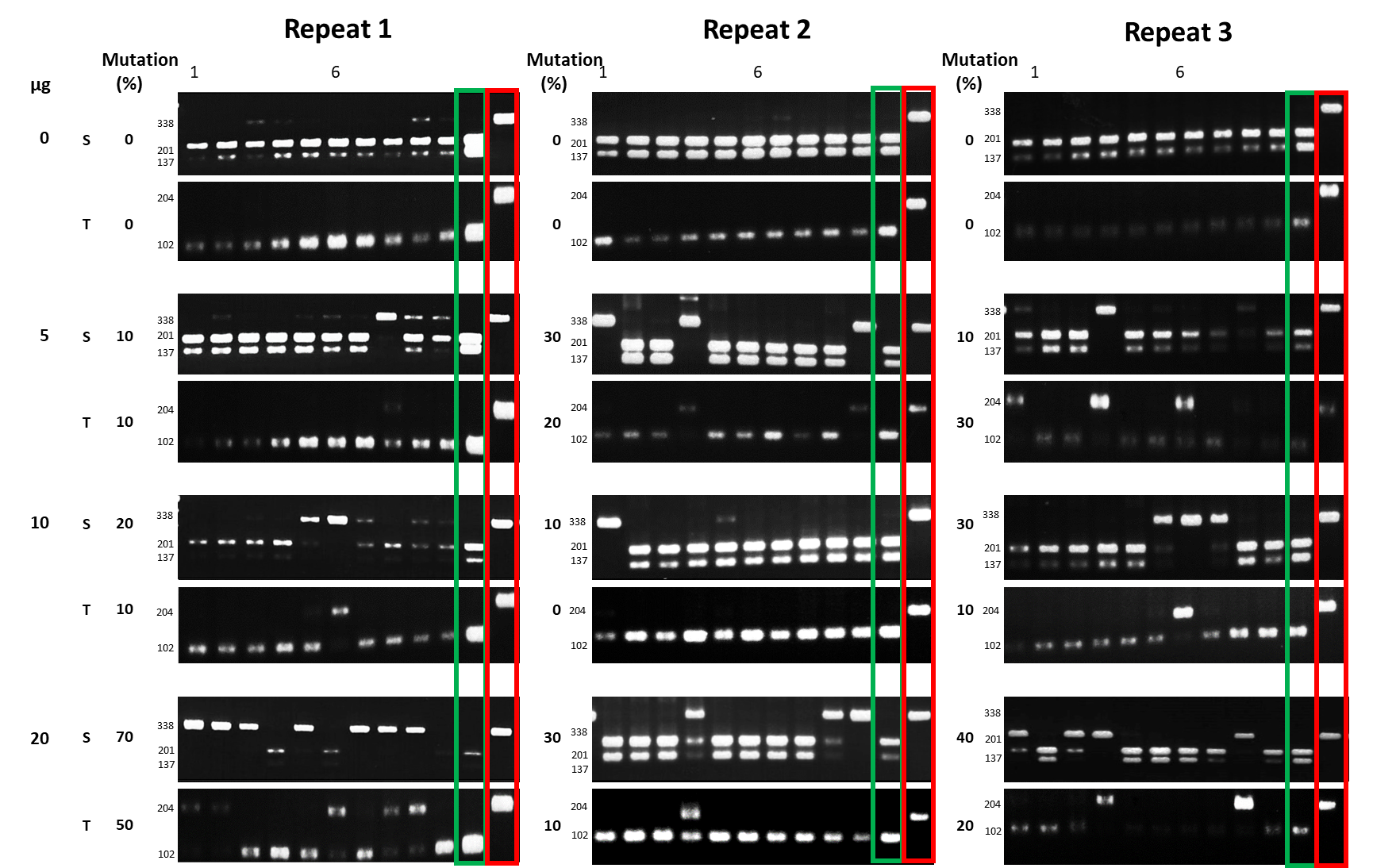


**Supplemental Figure 17. Effect of plasmid dosage on *NtPDS* mutagenesis in tobacco protoplast regenerants.**

Tobacco protoplasts were transfected with 0-20 µg plasmid DNA containing the expression cassettes *NtPDS* sgRNA and SaCas9 (Kaya et al., 2016) and regenerated into shoots. Targeted mutagenesis of 10 green regenerants from each treatment were analyzed by RFLP. S, *N. sylvestris* form; T, *N. tomentosiformis* form. Green frame, wild-type RFLP control; red frame, albino mutant RFLP control.


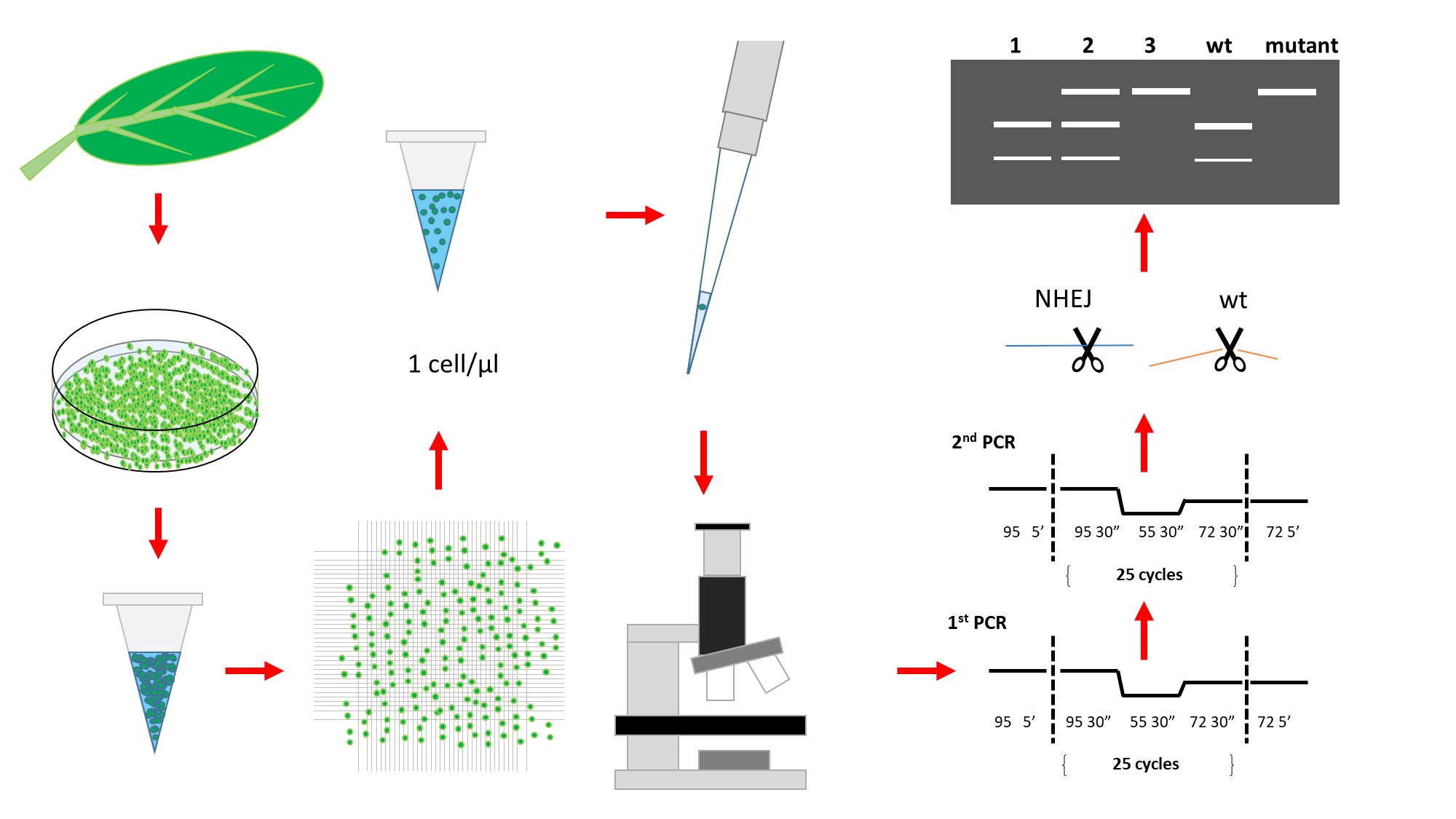


**Supplemental Figure 18. Schematic representation of single cell isolation and validation of targeted mutagenesis.**

Protoplasts are isolated and transfected (detailed information is given in Supplemental Table 1). The protoplasts are centrifuged and transferred to liquid callus medium (1/2 MS medium supplemented with 0.4 M mannitol, 30 g/L sucrose, 1 mg/L NAA, and 0.3 mg/L kinetin). The protoplast concentration is measured using a haemocytometer and adjusted to 1 cell/ul using the same medium. A single protoplast is isolated using a 2 µl laboratory pipette. The protoplast is transferred to a slide and the condition is checked by microscopy (Olympus, Tokyo, Japan). The protoplast is transferred to PCR mixture containing the first pair of primers for primary DNA amplification. One µl of product is used as the template for a second round of PCR using a second pair of primers. The second PCR product is digested by a restriction enzyme and analyzed by electrophoresis to validate mutagenesis of the target sequences.


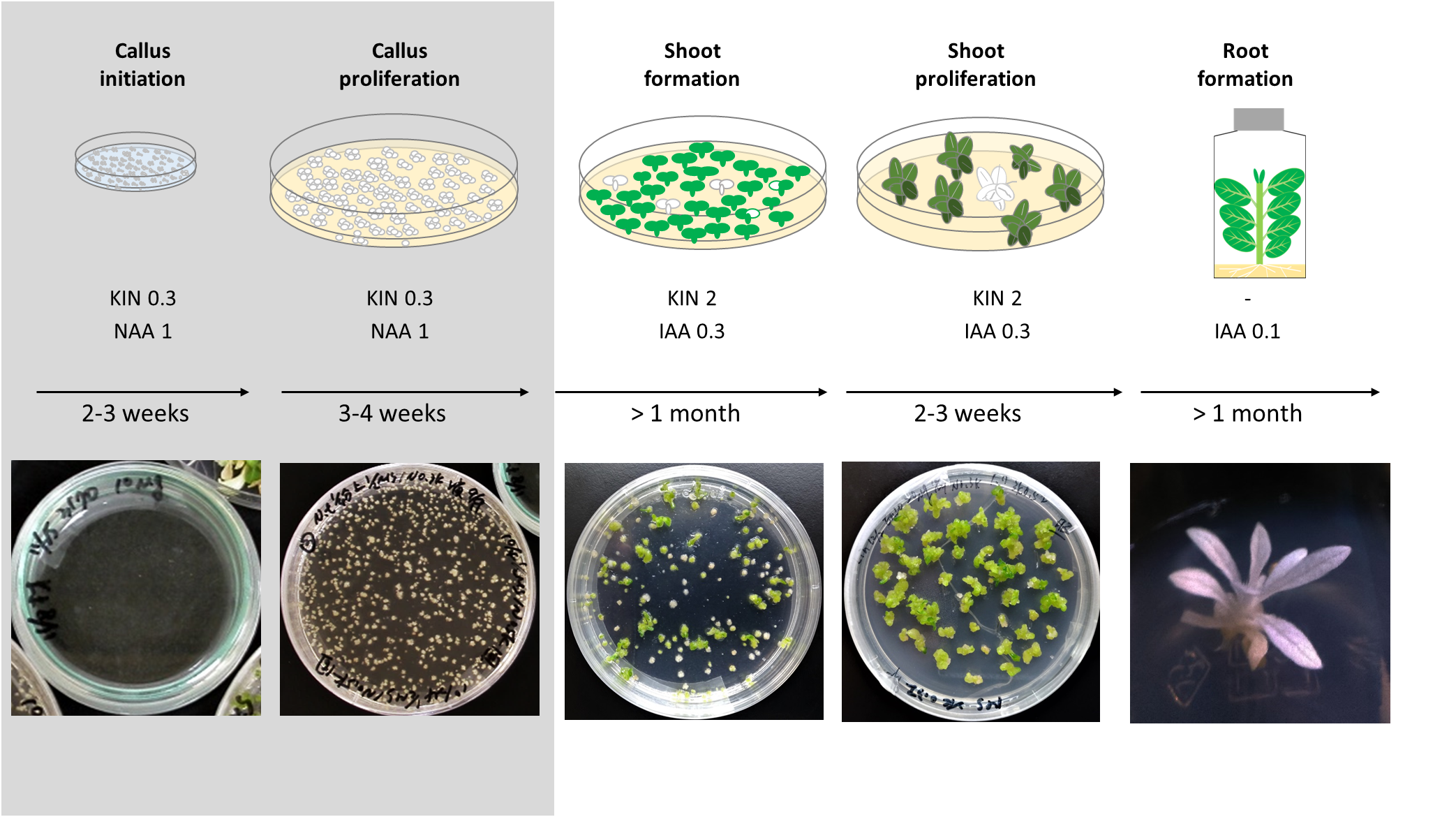


**Supplemental Figure 19. Schematic of tobacco protoplast regeneration.**

The protocol is modified from Liao (1990). The transfected protoplasts are incubated in a 5 cm diameter petri dish containing liquid callus medium (1/2MS medium supplemented with 0.4 M mannitol, 30 g/L sucrose, 1 mg/L NAA and 3 mg/L kinetin). After 2-3 weeks, the proliferating protoplasts form dust-like calli. The calli are embedded in solidified callus medium in a 9 cm diameter petri dish for 3-4 weeks. In the callus stage, the explants are incubated in the dark (gray background). Calli larger than 3 mm can be embedded in shooting medium (MS medium supplemented with 2 mg/L kinetin, 0.3 mg/L IAA, 0.4 M mannitol, and 30 g/L sucrose) for shoot induction. After one month, the multiple shoots which contain leaves or are of a size larger than 5 mm are transferred to fresh shooting medium for 2-3 weeks for shoot proliferation. Multiple shoots with leaves are transferred to solidified rooting medium (MS medium supplemented with 0.1 mg/L IAA, and 30 g/L sucrose). After shooting, all explants are incubated in light/dark cycling conditions (light/dark: 16/8 hrs). KIN: kinetin.

**Supplemental Material and Methods**

The pCAMBIA1300-OsU3-Cas9 and pCAMBIA1300-OsU6-Cas9 plasmids can be used for both protoplast transfection and *Agrobacterium*-mediated transformation. The nucleotide “A” was included immediately upstream of the 5′ end of the target sequence, and a “G” was included immediately upstream of the 5′ end of the target sequence, when cloned into the plasmids pOSU3-sgRNA or pOsU6-sgRNA, respectively. According to the target sequence, we synthesized a pair of complementary oligonucleotides with 5’ adapters (pOsU3-sgRNA: Fw 5'-GGCA-20 bp-3', Rv: 5'-AAAC-20 bp-3'; pOsU6-sgRNA: Fw 5'-CTTG-20 bp-3', Rv: 5'-AAAC-20 bp-3'). The pair of complementary oligonucleotides with their corresponding adapters was annealed, phosphorylated, and ligated to the linearized plasmids digested with *Aar*I (Fermentas/Thermo Scientific Inc., Waltham, MA) using T4 DNA ligase (New England Biolabs Inc., Ipswich, MA). The ligation mixture was transformed into *E. coli* DH5α, and at least eight clones were picked, subjected to PCR, and the PCR products of candidate clones were analyzed by Sanger DNA sequencing. A plasmid containing the correct target sequence was later used for transfection into protoplasts.
