# Supplementary material for: Application of protoplast technology to CRISPR/Cas9 mutagenesis: from single‐cell mutation detection to mutant plant regeneration
Source: Plant Biotechnol J. 2018 Jan 10;16(7):1295–310. doi: 10.1111/pbi.12870 (PMC5999315; doi:10.1111/pbi.12870)
Supplement: Supplementary file 2 — Table S1. Protocol for protoplast isolation and PEG transformation of different Poaceae species. Table S2 Protocol for protoplast isolation and PEG transformation of different Brassicaceae species. Table S3 Protocol for protoplast isolation and PEG transformation of different Solanaceae species. [file PBI-16-1295-s005.docx]

Supplemental Table 1. Protocol for protoplast isolation and PEG transformation of different Poaceae species

| **Species** | **Digestion Buffer** | **Digestion condition** | **Wash Condition** | **Centrifugation**  **Speed** | **Cell number** | **MMG Buffer** | **PEG solution** | **PEG Transfection time** |
| --- | --- | --- | --- | --- | --- | --- | --- | --- |
| *Setaria italica* | 1.5% (wt/vol) Cellulase R10,  0.75% (wt/vol) macerozyme R10,  10 mM MES (pH5.7),  0.6 M mannitol,  55 °C for 10 min, cool to RT,  10 mM CaCl_2_‧2H_2_O,  and 0.1% (wt/vol) BSA  sterilize buffer with a 0.45 μm filter | 3 hrs/50 rpm  (25℃) | W5 solution:  154 mM NaCl,  125 mM CaCl_2_‧2H_2_O,  5 mM KCl,  2 mM MES (pH5.7),  5 mM glucose,  centrifugation for 2 min  Wash twice  Keep cell on ice for 30 min | 100*g* | 1.2🞪10^7^ /g FW | 0.6M mannitol,  15 mM MgCl_2,_  4 mM MES (pH 5.7) | 40% (wt/vol) PEG 4000,  0.6 M mannitol ,  Warm the solution at 60°C until dissolved and cool down to 40°C  0.1 M CaCl_2_‧2H_2_O, | 20 min |
| *Oryza sativa* |  |  |  | 200*g* | 4.2🞪10^6^ /g FW |  |  |  |
| Triticum aestivum |  |  |  | 100*g* | 4.1🞪10^6^ /g FW |  |  |  |
| Zea mays | 1.5% (wt/vol) Cellulase R10,  0.75% (wt/vol) macerozyme R10,  10 mM MES (pH5.7),  0.6 M mannitol,  55 °C for 10 min, cool to RT  1 mM CaCl_2_‧2H_2_O  and 0.1% (wt/vol) BSA  sterilize buffer with a 0.45 μm filter |  |  | 150*g* | 2.3🞪10^6^ /g FW |  |  |  |

Supplemental Table 2. Protocol for protoplast isolation and PEG transformation of different Brassicaceae species

| **Species** | **Digestion Buffer** | **Digestion condition** | **Wash Condition** | **Centrifugation**  **Speed** | **Cell number** | **MMG Buffer** | **PEG solution** | **PEG Transfection time** |
| --- | --- | --- | --- | --- | --- | --- | --- | --- |
| *Arabidopsis thaliana* | 1% (wt/vol) Cellulase R10,  0.25% (wt/vol) macerozyme R10,  20 mM MES (pH5.7),  20 mM KCl,  0.4 M mannitol,  55 °C for 10 min, cool to RT  10 mM CaCl_2_‧2H_2_O  and 0.1% BSA  sterilize buffer with a 0.45 μm filter | 1.5hrs/50 rpm  (25℃) | W5 solution:  154 mM NaCl  125 mM CaCl_2_‧2H_2_O  5 mM KCl  2 mM MES (pH5.7)  5 mM glucose  Centrifugation for 2 min  Wash twice  Keep cell on ice for 30 min | 100*g* | 3.0 × 10^7^ /g FW | 0.4 M mannitol  15 mM MgCl_2_  4 mM MES (pH 5.7) | 40% (wt/vol) PEG 4000  0.2 M mannitol  0.1 M CaCl_2_‧2H_2_O | 6 min |
| *Brassica oleracea* | 1.5% (wt/vol) Cellulase R10,  0.75% (wt/vol) macerozyme R10,  10mM MES (pH5.7),  0.6M mannitol,  55 °C for 10 min, cool to RT  10 mM CaCl_2_‧2H_2_O  and 0.1% (wt/vol) BSA  sterilize buffer with a 0.45-μm filter | 3hrs/50 rpm  (25℃) |  |  | 4.2🞪10^6^ /g FW |  |  | 10 min |
| *Brassica napus* |  |  |  |  | 2.2🞪10^6^ /g FW | 0.6 M mannitol  15 mM MgCl_2_  4 mM MES (pH 5.7) | 40% (wt/vol) PEG 4000,  0.6 M mannitol,  Warm the solution at 60 °C until dissolved and cool down to 40°C  0.1 M CaCl_2_‧2H_2_O | 6 min |
| *Cleome* spp. |  | 1.5hrs/50 rpm  (25℃) |  |  | 3.5🞪10^6^ /g FW |  |  | 10 min |

Supplemental Table 3. Protocol for protoplast isolation and PEG transformation of different Solanaceae species

| **Species** | **Digestion Buffer** | **Digestion condition** | **Wash Condition** | **Centrifugation**  **Speed** | **Cell number** | **MMG Buffer** | **PEG solution** | **PEG Transfection time** |
| --- | --- | --- | --- | --- | --- | --- | --- | --- |
| Solanum tuberosum | 1.5% (wt/vol) Cellulase R10,  0.75% (wt/vol) macerozyme R10,  10 mM MES (pH5.7),  0.6 M mannitol,  55 °C for 10 min, cool to RT  1 mM CaCl_2_‧2H_2_O  and 0.1% (wt/vol) BSA  sterilize buffer with a 0.45 μm filter | 3 hrs/50 rpm  (25℃) | W5 solution:  154 mM NaCl,  125 mM CaCl_2_‧2H_2_O,  5 mM KCl,  2 mM MES (pH5.7),  5 mM glucose,  centrifugation for 2 min  Wash twice  Keep cell on ice for 30 min | 100*g* | 6.4🞪10^6^ /g FW | 0.6M mannitol,  15 mM MgCl_2,_  4 mM MES (pH 5.7) | 40% (wt/vol) PEG 4000,  0.6 M mannitol ,  Warm the solution at 60 °C until dissolved and cool down to 40°C  0.1 M CaCl_2_‧2H_2_O | 20 min |
| *Solanum lycopersicum* (cv microtom) suspension cells | 1.5% (wt/vol) Cellulase R10,  0.75% (wt/vol) macerozyme R10,  10 mM MES (pH5.7),  0.6 M mannitol,  10 mM sodium acetate  55 °C for 10 min, cool to RT,  10 mM CaCl_2_‧2H_2_O,  and 0.1% (wt/vol) BSA  sterilize buffer with a 0.45 μm filter | 4 hrs/50 rpm  (35℃) |  | 250*g* | 7.2🞪10^6^ /g FW |  |  |  |
| Nicotiana tabacum  BY-2 suspension cells |  |  |  | 250*g* | 1.4🞪10^7^/g FW |  |  |  |
