# Supplementary material for: Application of protoplast technology to CRISPR/Cas9 mutagenesis: from single‐cell mutation detection to mutant plant regeneration
Source: Plant Biotechnol J. 2018 Jan 10;16(7):1295–310. doi: 10.1111/pbi.12870 (PMC5999315; doi:10.1111/pbi.12870)
Supplement: Supplementary file 3 — Data S1 The NtPDS sequences of Figure 3 (Experiment 1) and Table 2. [file PBI-16-1295-s007.docx]

**Supplemental Data 1. The *NtPDS* sequences of Figure 3 (Experiment 1) and Table 2.** Red box in Sample no. column indicates all four copies of *NtPDS* have the same mutations. S, *N. sylvestris* form; T, *N. tomentosiformis* form. Orange box-Homo, homozygous in this form; Light blue box-hetero, heterozygous in this form; W, wild-type; I, insertion; D, deletion; Letter in red, the mutated nucleotide.

**Experiment 1**

| **Sample no.** | **Genome type** | **genotype** | **Mutation type** | ***NtPDS* sequences** |  |
| --- | --- | --- | --- | --- | --- |
| Wild type | S |  | W | GATGCCTAACAAGC-CAGGGGAG |  |
|  | T |  | W | GATGCCTAACAAGC-CAGGGGAA |  |
|  | | | | | |
| R1 5 µg-12 | S | Hetero | W | GATGCCTAACAAGC-CAGGGGAG |  |
|  |  |  | I | GATGCCTAACAAGCTCAGGGGAG |  |
| R1 5 µg-18 | S | Hetero | W | GATGCCTAACAAGC-CAGGGGAG |  |
|  |  |  | I | GATGCCTAACAAGCGCAGGGGAG |  |
| R1 5 µg-21 | T | Hetero | W | GATGCCTAACAAGC-CAGGGGAA |  |
|  |  |  | I | GATGCCTAACAAGCGCAGGGGAA |  |
| R1 5 µg-22 | T | Hetero | W | GATGCCTAACAAGC-CAGGGGAA |  |
|  |  |  | I | GATGCCTAACAAGCTCAGGGGAA |  |
|  | | | | | |
| R1 10 µg-3 | T | Hetero | W | GATGCCTAACAAGC-CAGGGGAA |  |
|  |  |  | I | GATGCCTAACAAGCGCAGGGGAA |  |
| R1 10 µg-4 | S | Hetero | I | GATGCCTAACAAGCACAGGGGAG |  |
|  |  |  | D | GATGCCTAACAAGC--GGGGGAG |  |
| R1 10 µg-8 | S | Hetero | W | GATGCCTAACAAGC-CAGGGGAG |  |
|  |  |  | I | GATGCCTAACAAGCGCAGGGGAG |  |
|  | T | Hetero | W | GATGCCTAACAAGC-CAGGGGAA |  |
|  |  |  | I | GATGCCTAACAAGCGCAGGGGAA |  |
| R1 10 µg-14 | S | Hetero | W | GATGCCTAACAAGC-CAGGGGAG |  |
|  |  |  | I | GATGCCTAACAAGCTCAGGGGAG |  |
| R1 10 µg-16 | S | Hetero | W | GATGCCTAACAAGC-CAGGGGAG |  |
|  |  |  | I | GATGCCTAACAAGCGCAGGGGAG |  |
| R1 10 µg-19 | S | Hetero | W | GATGCCTAACAAGC-CAGGGGAG |  |
|  |  |  | I | GATGCCTAACAAGCTCAGGGGAG |  |
|  | | | | | |
| R1 20 µg-1 | S | Hetero | W | GATGCCTAACAAGC-CAGGGGAG |  |
|  |  |  | I | GATGCCTAACAAGCTCAGGGGAG |  |
| R1 20 µg-6 | S | Hetero | W | GATGCCTAACAAGC-CAGGGGAG |  |
|  |  |  | I | GATGCCTAACAAGC-(+12 bps) |  |
| R1 20 µg-8 | S | Hetero | W | GATGCCTAACAAGC-CAGGGGAG |  |
|  |  |  | I | GATGCCTAACAAGCTCAGGGGAG |  |
| R1 20 µg-9 | S | Hetero | W | GATGCCTAACAAGC-CAGGGGAG |  |
|  |  |  | I | GATGCCTAACAAGCACAGGGGAG |  |
| R1 20 µg-13 | S | Hetero | W | GATGCCTAACAAGC-CAGGGGAG |  |
|  |  |  | I | GATGCCTAACAAGCACAGGGGAG |  |
| R1 20 µg-16 | S | Homo | I | GATGCCTAACAAGCTCAGGGGAG |  |
|  |  |  | I | GATGCCTAACAAGCTCAGGGGAG |  |
| R1 20 µg-18 | S | Hetero | W | GATGCCTAACAAGC-CAGGGGAG |  |
|  |  | Hetero | I | GATGCCTAACAAGCACAGGGGAG |  |
| R1 20 µg-19 | T | Homo | I | GATGCCTAACAAGCTCAGGGGAA |  |
|  |  |  | I | GATGCCTAACAAGCTCAGGGGAA |  |
| R1 20 µg-21 | S | Hetero | W | GATGCCTAACAAGC-CAGGGGAG |  |
|  |  |  | I | GATGCCTAACAAGCTCAGGGGAG |  |
|  | T | Hetero | W | GATGCCTAACAAGC-CAGGGGAA |  |
|  |  |  | I | GATGCCTAACAAGCTCAGGGGAA |  |

**Experiment 2**

| **Sample no.** | **Genome type** | **genotype** | **Mutation type** | ***NtPDS* sequences** |  |
| --- | --- | --- | --- | --- | --- |
| Wild type | S |  | W | GATGCCTAACAAGC-CAGGGGAG |  |
|  | T |  | W | GATGCCTAACAAGC-CAGGGGAA |  |
|  | | | | | |
| R2 5 µg-1 | S | Hetero | W | GATGCCTAACAAGC-CAGGGGAG |  |
|  |  |  | I | GATGCCTAACAAGCTCAGGGGAG |  |
| R2 5 µg-5 | S | Hetero | W | GATGCCTAACAAGC-CAGGGGAG |  |
|  |  |  | I | GATGCCTAACAAGCACAGGGGAG |  |
| R2 5 µg-6 | S | Hetero | W | GATGCCTAACAAGC-CAGGGGAG |  |
|  |  |  | I | GATGCCTAACAAGCGCAGGGGAG |  |
|  | T | Homo | I | GATGCCTAACAAGCTCAGGGGAA |  |
|  |  |  | I | GATGCCTAACAAGCTCAGGGGAA |  |
| R2 5 µg-8 | S | Hetero | W | GATGCCTAACAAGC-CAGGGGAG |  |
|  |  |  | I | GATGCCTAACAAGCTCAGGGGAG |  |
| R2 5 µg-9 | S | Hetero | W | GATGCCTAACAAGC-CAGGGGAG |  |
|  |  |  | I | GATGCCTAACAAGCACAGGGGAG |  |
| R2 5 µg-10 | S | Hetero | W | GATGCCTAACAAGC-CAGGGGAG |  |
|  |  |  | I | GATGCCTAACAAGCTCAGGGGAG |  |
| R2 5 µg-12 | S | Hetero | W | GATGCCTAACAAGC-CAGGGGAG |  |
|  |  |  | I | GATGCCTAACAAGCTCAGGGGAG |  |
| R2 5 µg-13 | S | Hetero | W | GATGCCTAACAAGC-CAGGGGAG |  |
|  |  |  | I | GATGCCTAACAAGCACAGGGGAG |  |
| R2 5 µg-15 | S | Hetero | W | GATGCCTAACAAGC-CAGGGGAG |  |
|  |  |  | D | GATGCCTAACAAGC-(-8 bps) |  |
| R2 5 µg-16 | S | Homo | I | GATGCCTAACAAGCTCAGGGGAG |  |
|  |  |  | I | GATGCCTAACAAGCTCAGGGGAG |  |
| R2 5 µg-17 | S | Hetero | W | GATGCCTAACAAGC-CAGGGGAG |  |
|  |  |  | I | GATGCCTAACAAGCGCAGGGGAG |  |
|  | T | Homo | I | GATGCCTAACAAGCTCAGGGGAA |  |
|  |  |  | I | GATGCCTAACAAGCTCAGGGGAA |  |
| R2 5 µg-18 | T | Homo | I | GATGCCTAACAAGCTCAGGGGAA |  |
|  |  |  | I | GATGCCTAACAAGCTCAGGGGAA |  |
| R2 5 µg-19 | S | Homo | I | GATGCCTAACAAGCTCAGGGGAG |  |
|  |  |  | I | GATGCCTAACAAGCTCAGGGGAG |  |
| R2 5 µg-20 | S | Homo | I | GATGCCTAACAAGCTCAGGGGAA |  |
|  |  |  | I | GATGCCTAACAAGCTCAGGGGAA |  |
|  | | | | | |
| R2 10 µg-2 | S | Hetero | W | GATGCCTAACAAGC-CAGGGGAG |  |
|  |  |  | I | GATGCCTAACAAGCTCAGGGGAG |  |
| R2 10 µg-3 | S | Homo | D | GATGCCTAACAAAC--AGGGGAA |  |
|  |  |  | D | GATGCCTAACAAGC--AGGGGAG |  |
| R2 10 µg-4 | S | Hetero | W | GATGCCTAACAAGC-CAGGGGAG |  |
|  |  | Hetero | I | GATGCCTAACAAGCTCAGGGGAG |  |
|  | T | Homo | D | GATGCCTAACAAAC--AGGGGAA |  |
|  |  |  | D | GATGCCTAACAAGC--AGGGGAA |  |
| R2 10 µg-5 | S | Hetero | W | GATGCCTAACAAGC-CAGGGGAA |  |
|  |  |  | I | GATGCCTAACAAGCTCAGGGGAA |  |
| R2 10 µg-6 | S | Homo | I | GATGCCTAACAAGCTCAGGGGAG |  |
|  |  |  | I | GATGCCTAACAAGCTCAGGGGAG |  |
| R2 10 µg-7 | S | Hetero | W | GATGCCTAACAAGC-CAGGGGAG |  |
|  |  |  | I | GATGCCTAACAAGCTCAGGGGAG |  |
|  | T | Hetero | W | GATGCCTAACAAGC-CAGGGGAA |  |
|  |  |  | I | GATGCCTAACAAGCTCAGGGGAA |  |
| R2 10 µg-8 | T | Hetero | W | GATGCCTAACAAGC-CAGGGGAA |  |
|  |  |  | I | GATGCCTAACAAGCTCAGGGGAA |  |
| R2 10 µg-9 | T | Hetero | W | GATGCCTAACAAGC-CAGGGGAA |  |
|  |  |  | I | GATGCCTAACAAGCTCAGGGGAA |  |
| R2 10 µg-10 | S | Hetero | W | GATGCCTAACAAGC-CAGGGGAG |  |
|  |  |  | I | GATGCCTAACAAGCCCTAGGGGAG |  |
| R2 10 µg-11 | S | Hetero | I | GATGCCTAACAAGCTCAGGGGAG |  |
|  |  |  | D | GATGCCTAACAAGC--AGGGGAG |  |
| R2 10 µg-12 | S | Hetero | W | GATGCCTAACAAGC-CAGGGGAG |  |
|  |  |  | I | GATGCCTAACAAGCGCAGGGGAG |  |
|  | T | Hetero | W | GATGCCTAACAAGC-CAGGGGAA |  |
|  |  |  | I | GATGCCTAACAAGCCTAGGGGAA |  |
| R2 10 µg-13 | S | Hetero | I | GATGCCTAACAAGCTCAGGGGAG |  |
|  |  |  | I | GATGCCTAACAAGCTTCAGGGGA |  |
| R2 10 µg-14 | S | Homo | I | GATGCCTAACAAGCTCAGGGGAG |  |
|  |  |  | I | GATGCCTAACAAGCTCAGGGGAG |  |
| R2 10 µg-16 | S | Hetero | W | GATGCCTAACAAGC-CAGGGGAG |  |
|  |  |  | I | GATGCCTAACAAGCTCAGGGGAG |  |
|  | T | Homo | I | GATGCCTAACAAGCTCAGGGGAA |  |
|  |  |  | I | GATGCCTAACAAGCTCAGGGGAA |  |
| R2 10 µg-18 | S | Hetero | W | GATGCCTAACAAGC-CAGGGGAG |  |
|  |  | Hetero | I | GATGCCTAACAAGCACAGGGGAG |  |
|  | T | Homo | I | GATGCCTAACAAGCTCAGGGGAA |  |
|  |  |  | I | GATGCCTAACAAGCTCAGGGGAA |  |
| R2 10 µg-20 | S | Hetero | D | GATGCCTAACAA-C-CAGGGGAG |  |
|  |  | Hetero | D | GATGCCTAACATG---AGGGGAG |  |
|  | T | Homo | I | GATGCCTAACAAGCTCAGGGGAA |  |
|  |  |  | I | GATGCCTAACAAGCTCAGGGGAA |  |
|  | | | | | |
| R2 20 µg-1 | S | Homo | I | GATGCCTAACAAGC(+106 bps) |  |
|  |  |  | I | GATGCCTAACAAGC(+106 bps) |  |
|  | T | Homo | D | GATGCCTAACAAGC--AGGGGAA |  |
|  |  |  | D | GATGCCTAACAAGC--AGGGGAA |  |
| R2 20 µg-2 | S | Hetero | D | GATGCCTAACAA---CAGGGGAG |  |
|  |  |  | I | GATGCCTAACAAGCTCAGGGGAG |  |
|  | T | Homo | D | GATGCCTAACAA---CAGGGGAG |  |
|  |  |  | D | GATGCCTAACAA---CAGGGGAG |  |
| R2 20 µg-3 | S | Hetero | W | GATGCCTAACAAGC-CAGGGGAG |  |
|  |  |  | I | GATGCCTAACAAGCTCAGGGGAG |  |
| R2 20 µg-4 | S | Hetero | I | GATGCCTAACAAGCTCAGGGGAG |  |
|  |  |  | I | GATGCCTAACAAGCGCAGGGGAG |  |
| R2 20 µg-5 | T | Homo | I | GATGCCTAACAAGCTCAGGGGAA |  |
|  |  |  | I | GATGCCTAACAAGCTCAGGGGAA |  |
| R2 20 µg-6 | S | Homo | I | GATGCCTAACAAGCTCAGGGGAG |  |
|  |  |  | I | GATGCCTAACAAGCTCAGGGGAG |  |
|  | T | Hetero | W | GATGCCTAACAAGC-CAGGGGAA |  |
|  |  |  | I | GATGCCTAACAAGCTCAGGGGAA |  |
| R2 20 µg-7 | S | Hetero | W | GATGCCTAACAAGC-CAGGGGAG |  |
|  |  |  | I | GATGCCTAACAAGCACAGGGGAG |  |
| R2 20 µg-8 | S | Hetero | W | GATGCCTAACAAGC-CAGGGGAG |  |
|  |  |  | I | GATGCCTAACAAGCTCAGGGGAG |  |
| R2 20 µg-9 | S | Hetero | W | GATGCCTAACAAGC-CAGGGGAG |  |
|  |  |  | I | GATGCCTAACAAGCACAGGGGAG |  |
|  | T | Hetero | W | GATGCCTAACAAGC-CAGGGGAA |  |
|  |  |  | I | GATGCCTAACAAGCACAGGGGAA |  |
| R2 20 µg-10 | S | Hetero | W | GATGCCTAACAAGC-CAGGGGAG |  |
|  |  |  | I | GATGCCTAACAAGCACAGGGGAG |  |
|  | T | Homo | I | GATGCCTAACAAGCTCAGGGGAA |  |
|  |  |  | I | GATGCCTAACAAGCTCAGGGGAA |  |
| R2 20 µg-11 | S | Hetero | I | GATGCCTAACAAGCACAGGGGAG |  |
|  |  |  | I | GATGCCTAACAAGCTCAGGGGAG |  |
| R2 20 µg-12 | S | Hetero | I | GATGCCTAACAAGCTCAGGGGAG |  |
|  |  |  | I | GATGCCTAACAAGCGCATGGGAG |  |
|  | T | Hetero | I | GATGCCTAACAAGCACAGGGGAA |  |
|  |  |  | I | GATGCCTAACAAGCTCAGGGGAA |  |
| R2 20 µg-13 | S | Hetero | I | GATGCCTAACAAGCTCAGGGGAG |  |
|  |  |  | D | GATGCCTAACAAGC--AGGGGAG |  |
|  | T | Hetero | D | GATGCCTAACAAGC--AGGGGAA |  |
|  |  |  | I | GATGCCTAACAAGCTCAGGGGAA |  |
| R2 20 µg-14 | S | Homo | I | GATGCCTAACAAGCTCAGGGGAG |  |
|  |  |  | I | GATGCCTAACAAGCTCAGGGGAG |  |
| R2 20 µg-16 | T | Hetero | D | GATGCCTAACAAGC--AGGGGAA |  |
|  |  |  | I | GATGCCTAACAAGCACAGGGGAA |  |
| R2 20 µg-17 | S | Homo | I | GATGCCTAACAAGCTCAGGGGAG |  |
|  |  |  | I | GATGCCTAACAAGCTCAGGGGAG |  |
| R2 20 µg-18 | S | Homo | I | GATGCCTAACAAGCTCAGGGGAG |  |
|  |  |  | I | GATGCCTAACAAGCTCAGGGGAG |  |
| R2 20 µg-19 | S | Hetero | W | GATGCCTAACAAGC-CAGGGGAG |  |
|  |  |  | I | GATGCCTAACAAGC-(+11 bps) |  |
|  | T | Hetero | W | GATGCCTAACAAGC-CAGGGGAA |  |
|  |  |  | I | GATGCCTAACAAGCTCAGGGGAA |  |
| R2 20 µg-20 | S | Hetero | I | GATGCCTAACAAGCGCAGGGGAG |  |
|  |  |  | I | GATGCCTAACAAGCACAGGGGAG |  |

**Experiment 3**

| **Sample no.** | **Genome type** | **genotype** | **Mutation type** | ***NtPDS* sequences** |  |
| --- | --- | --- | --- | --- | --- |
| Wild type | S |  | W | GATGCCTAACAAGC-CAGGGGAG |  |
|  | T |  | W | GATGCCTAACAAGC-CAGGGGAA |  |
|  | | | | | |
| R3 5 µg-2 | T | Hetero | W | GATGCCTAACAAGC-CAGGGGAA |  |
|  |  |  | I | GATGCCTAACAAGCTCAGGGGAA |  |
| R3 5 µg-10 | S | Hetero | W | GATGCCTAACAAGC-CAGGGGAG |  |
|  |  |  | I | GATGCCTAACAAGCTCAGGGGAG |  |
| R3 5 µg-14 | S | Hetero | W | GATGCCTAACAAGC-CAGGGGAG |  |
|  |  |  | I | GATGCCTAACAAGCTCAGGGGAG |  |
|  | | | | | |
| R3 10 µg-1 | S | Hetero | W | GATGCCTAACAAGC-CAGGGGAG |  |
|  |  |  | I | GATGCCTAACAAGCTCAGGGGAG |  |
| R3 10 µg-4 | S | Hetero | W | GATGCCTAACAAGC-CAGGGGAG |  |
|  |  |  | I | GATGCCTAACAAGCTCAGGGGAG |  |
| R3 10 µg-5 | S | Hetero | W | GATGCCTAACAAGC-CAGGGGAG |  |
|  |  |  | I | GATGCCTAACAAGCACAGGGGAG |  |
| R3 10 µg-8 | S | Hetero | W | GATGCCTAACAAGC-CAGGGGAG |  |
|  |  |  | I | GATGCCTAACAAGCTCAGGGGAG |  |
| R3 10 µg-9 | S | Hetero | W | GATGCCTAACAAGC-CAGGGGAG |  |
|  |  |  | I | GATGCCTAACAAGCTCAGGGGAG |  |
|  | T | Hetero | W | GATGCCTAACAAGC-CAGGGGAA |  |
|  |  |  | I | GATGCCTAACAAGCTCAGGGGAA |  |
| R3 10 µg-10 | S | Hetero | W | GATGCCTAACAAGC-CAGGGGAG |  |
|  |  |  | I | GATGCCTAACAAGCTCAGGGGAG |  |
|  | T | Hetero | W | GATGCCTAACAAGC-CAGGGGAA |  |
|  |  |  | I | GATGCCTAACAAGCTCAGGGGAA |  |
| R3 10 µg-11 | S | Hetero | I | GATGCCTAACAAGCTCAGGGGAG |  |
|  |  |  | I | GATGCCTAACAAGCTCAGGGGAG |  |
|  | T | Homo | I | GATGCCTAACAAGCACAGGGGAA |  |
|  |  |  | I | GATGCCTAACAAGCACAGGGGAA |  |
| R3 10 µg-15 | S | Homo | I | GATGCCTAACAAGCTCAGGGGAG |  |
|  |  |  | I | GATGCCTAACAAGCTCAGGGGAG |  |
|  | T | Hetero | W | GATGCCTAACAAGC-CAGGGGAA |  |
|  |  |  | I | GATGCCTAACAAGCGCAGGGGAA |  |
| R3 10 µg-16 | S | Hetero | W | GATGCCTAACAAGC-CAGGGGAG |  |
|  |  |  | I | GATGCCTAACAAGCGCAGGGGAG |  |
| R3 10 µg-17 | S | Hetero | W | GATGCCTAACAAGC-CAGGGGAG |  |
|  |  |  | D | GATGCCTAACAAGC-(-11 bps) |  |
| R3 10 µg-19 | S | Homo | I | GATGCCTAACAAGCACAGGGGAG |  |
|  |  |  | I | GATGCCTAACAAGCACAGGGGAG |  |
|  | T | Homo | I | GATGCCTAACAAGCACAGGGGAG |  |
|  |  |  | I | GATGCCTAACAAGCACAGGGGAG |  |
| R3 10 µg-20 | S | Hetero | I | GATGCCTAACAAGCACAGGGGAG |  |
|  |  |  | I | GATGCCTAACAAGCTCAGGGGAG |  |
|  | | | | | |
| R3 20 µg-1 | S | Hetero | W | GATGCCTAACAAGC-CAGGGGAG |  |
|  |  |  | I | GATGCCTAACAAGCTCAGGGGAG |  |
| R3 20 µg-7 | S | Hetero | W | GATGCCTAACAAGC-CAGGGGAG |  |
|  |  |  | I | GATGCCTAACAAGCACAGGGGAG |  |
|  | T | Hetero | W | GATGCCTAACAAGC-CAGGGGAA |  |
|  |  |  | I | GATGCCTAACAAGCACAGGGGAA |  |
| R3 20 µg-8 | S | Hetero | W | GATGCCTAACAAGC-CAGGGGAG |  |
|  |  |  | I | GATGCCTAACAAGCACAGGGGAG |  |
| R3 20 µg-9 | S | Hetero | W | GATGCCTAACAAGC-CAGGGGAG |  |
|  |  |  | I | GATGCCTAACAAGCTCAGGGGAG |  |
| R3 20 µg-11 | S | Hetero | W | GATGCCTAACAAGC-CAGGGGAG |  |
|  |  |  | I | GATGCCTAACAAGCTCAGGGGAG |  |
|  | T | Hetero | W | GATGCCTAACAAGC-CAGGGGAA |  |
|  |  |  | I | GATGCCTAACAAGCTCAGGGGAA |  |
| R3 20 µg-13 | S | Hetero | W | GATGCCTAACAAGC-CAGGGGAG |  |
|  |  |  | I | GATGCCTAACAAGCTCAGGGGAG |  |
|  | T | Hetero | W | GATGCCTAACAAGC-CAGGGGAA |  |
|  |  |  | I | GATGCCTAACAAGCACAGGGGAA |  |
| R3 20 µg-14 | S | Hetero | W | GATGCCTAACAAGC-CAGGGGAG |  |
|  |  |  | I | GATGCCTAACAAGCTCAGGGGAG |  |
| R3 20 µg-17 | S | Hetero | W | GATGCCTAACAAGC-CAGGGGAG |  |
|  |  |  | I | GATGCCTAACAAGCTCAGGGGAG |  |
|  | T | Hetero | W | GATGCCTAACAAGC-CAGGGGAA |  |
|  |  |  | I | GATGCCTAACAAGCTCAGGGGAA |  |
| R3 20 µg-18 | S | Hetero | I | GATGCCTAACAAGCACAGGGGAG |  |
|  |  |  | I | GATGCCTAACAAGCCCAGGGGAG |  |
|  | T | Hetero | I | GATGCCTAACAAGCACAGGGGAG |  |
|  |  |  | I | GATGCCTAACAAGCCCAGGGGAG |  |
| R3 20 µg-20 | T | Hetero | W | GATGCCTAACAAGC-CAGGGGAA |  |
|  |  |  | I | GATGCCTAACAAGCTCAGGGGAA |  |
