# Supplementary material for: Application of protoplast technology to CRISPR/Cas9 mutagenesis: from single‐cell mutation detection to mutant plant regeneration
Source: Plant Biotechnol J. 2018 Jan 10;16(7):1295–310. doi: 10.1111/pbi.12870 (PMC5999315; doi:10.1111/pbi.12870)
Supplement: Supplementary file 4 — Data S2 The NtPDS sequences of Figure 4 (Experiment 1) and Figure S15 (Experiment 2 and 3). [file PBI-16-1295-s001.docx]

**Supplemental Data 2. The *NtPDS* sequences of Figure 4 (Experiment 1) and Supplemental Figure 15 (Experiment 2 and 3).** Red box in Sample no. column indicates four copies of *NtPDS* have same mutations. S: *sylvestris* form. T: *tomentosiformis* form. Orange box-Homo: homozygous in this form. Light blue box-hetero: heterozygous in this form. W: wild type. I: insertion. D: deletion. Letter in red: the mutated nucleotide.

**Experiment 1**

| **Sample no.** | **Genome type** | **genotype** | **Mutation type** | ***NtPDS* sequences** |  |
| --- | --- | --- | --- | --- | --- |
| Wild type | S |  | W | GATGCCTAACAAGC-CAGGGGAG |  |
|  | T |  | W | GATGCCTAACAAGC-CAGGGGAA |  |
|  | | | | | |
| R1 0 day-5 | S | Homo | I | GATGCCTAACAAGCACAGGGGAG |  |
|  |  |  | I | GATGCCTAACAAGCACAGGGGAG |  |
| R1 0 day-14 | S | Hetero | W | GATGCCTAACAAGC-CAGGGGAG |  |
|  |  |  | I | GATGCCTAACAAGCGCAGGGGAG |  |
|  | T | Hetero | W | GATGCCTAACAAGC-CAGGGGAA |  |
|  |  |  | I | GATGCCTAACAAGCGCAGGGGAA |  |
|  | | | | | |
| R1 1 day-9 | S | Hetero | W | GATGCCTAACAAGC-CAGGGGAG |  |
|  |  |  | I | GATGCCTAACAAGCACAGGGGAG |  |
| R1 1 day-17 | S | Hetero | W | GATGCCTAACAAGC-CAGGGGAG |  |
|  |  |  | I | GATGCCTAACAAGCACAGGGGAG |  |
|  | | | | | |
| R1 2 day-5 | T | Homo | I | GATGCCTAACAAGCTCAGGGGAA |  |
|  |  |  | I | GATGCCTAACAAGCTCAGGGGAA |  |
| R1 2 day-6 | T | Homo | D | GATGCCT(-17 bps) |  |
|  |  |  | D | GATGCCT(-17 bps) |  |
| R1 2 day-9 | S | Homo | I | GATGCCTAACAAGCACAGGGGAG |  |
|  |  |  | I | GATGCCTAACAAGCACAGGGGAG |  |
| R1 2 day-11 | T | Hetero | W | GATGCCTAACAAGC-CAGGGGAA |  |
|  |  |  | I | GATGCCTAACAAGCTCAGGGGAA |  |
| R1 2 day-12 | S | Hetero | W | GATGCCTAACAAGC-CAGGGGAG |  |
|  |  |  | I | GATGCCTAACAAGCTCAGGGGAG |  |
|  | T | Hetero | W | GATGCCTAACAAGC-CAGGGGAA |  |
|  |  |  | I | GATGCCTAACAAGCACAGGGGAA |  |
| R1 2 day-15 | S | Hetero | W | GATGCCTAACAAGC-CAGGGGAG |  |
|  |  |  | I | GATGCCTAACAAGCACAGGGGAG |  |
| R1 2 day-16 | S | Homo | I | GATGCCTAACAAGCTCAGGGGAG |  |
|  |  |  | I | GATGCCTAACAAGCTCAGGGGAG |  |
| R1 2 day-17 | S | Homo | I | GATGCCTAACAAGCACAGGGGAG |  |
|  |  |  | I | GATGCCTAACAAGCACAGGGGAG |  |
| R1 2 day-19 | S | Homo | I | GATGCCTAACAAGATCAGGGGAG |  |
|  |  |  | I | GATGCCTAACAAGATCAGGGGAG |  |
| R1 2 day-20 | S | Hetero | W | GATGCCTAACAAGC-CAGGGGAG |  |
|  |  |  | I | GATGCCTAACAAGATCAGGGGAG |  |
|  | | | | | |
| R1 3 day-1 | S | Hetero | W | GATGCCTAACAAGC-CAGGGGAG |  |
|  |  |  | I | GATGCCTAACAAGCTCAGGGGAG |  |
| R1 3 day-6 | S | Hetero | W | GATGCCTAACAAGC-CAGGGGAG |  |
|  |  |  | I | GATGCCTAACAAGCTCAGGGGAG |  |
| R1 3 day-8 | S | Homo | I | GATGCCTAACAAGCGCAGGGGAG |  |
|  |  |  | I | GATGCCTAACAAGCGCAGGGGAG |  |
| R1 3 day-11 | S | Hetero | W | GATGCCTAACAAGC-CAGGGGAG |  |
|  |  |  | I | GATGCCTAACAAGCTCAGGGGAG |  |
| R1 3 day-13 | S | Hetero | W | GATGCCTAACAAGC-CAGGGGAG |  |
|  |  |  | I | GATGCCTAACAAGCTCAGGGGAG |  |
|  | T | Hetero | W | GATGCCTAACAAGC-CAGGGGAA |  |
|  |  |  | I | GATGCCTAACAAGCACAGGGGAA |  |
| R1 3 day-14 | S | Hetero | W | GATGCCTAACAAGC-CAGGGGAG |  |
|  |  |  | I | GATGCCTAACAAGCACAGGGGAG |  |
| R1 3 day-15 | T | Hetero | W | GATGCCTAACAAGC-CAGGGGAA |  |
|  |  |  | I | GATGCCTAACAAGCACAGGGGAA |  |
|  | | | | | |
| R1 4 day-3 | S | Hetero | W | GATGCCTAACAAGC-CAGGGGAG |  |
|  |  |  | I | GATGCCTAACAAGCACAGGGGAG |  |
| R1 4 day-4 | S | Hetero | W | GATGCCTAACAAGC-CAGGGGAG |  |
|  |  |  | I | GATGCCTAACAAGCACAGGGGAG |  |
| R1 4 day-6 | S | Hetero | W | GATGCCTAACAAGC-CAGGGGAG |  |
|  |  |  | I | GATGCCTAACAAGCACAGGGGAG |  |
| R1 4 day-9 | S | Hetero | W | GATGCCTAACAAGC-CAGGGGAG |  |
|  |  |  | I | GATGCCTAACAAGCTCAGGGGAG |  |
| R1 4 day-11 | S | Hetero | W | GATGCCTAACAAGC-CAGGGGAG |  |
|  |  |  | I | GATGCCTAACAAGCTCAGGGGAG |  |
| R1 4 day-15 | S | Hetero | W | GATGCCTAACAAGC-CAGGGGAG |  |
|  |  |  | I | GATGCCTAACAAGCGCAGGGGAG |  |
|  | T | Hetero | W | GATGCCTAACAAGC-CAGGGGAA |  |
|  |  |  | I | GATGCCTAACAAGCCAAGGGGAA |  |
| R1 4 day-16 | T | Hetero | W | GATGCCTAACAAGC-CAGGGGAA |  |
|  |  |  | I | GATGCCTAACAAGCCAAGGGGAA |  |

**Experiment 2**

| **Sample no.** | **Genome type** | **genotype** | **Mutation type** | **NtPDS sequences** |  |
| --- | --- | --- | --- | --- | --- |
| Wild type | S |  | W | GATGCCTAACAAGC-CAGGGGAG |  |
|  | T |  | W | GATGCCTAACAAGC-CAGGGGAA |  |
|  | | | | | |
| R2 0 day-9 | S | Hetero | W | GATGCCTAACAAGC-CAGGGGAG |  |
|  |  |  | I | GATGCCTAACAAGCTCAGGGGAG |  |
| R2 0 day-17 | T | Hetero | W | GATGCCTAACAAGC-CAGGGGAA |  |
|  |  |  | I | GATGCCTAACAAGCTCAGGGGAA |  |
|  | | | | | |
| R2 1 day-2 | S | Hetero | W | GATGCCTAACAAGC-CAGGGGAG |  |
|  |  |  | I | GATGCCTAACAAGCCTCAGGGGA |  |
|  | T | Homo | I | GATGCCTAACAAGCACAGGGGAA |  |
|  |  |  | I | GATGCCTAACAAGCACAGGGGAA |  |
| R2 1 day-9 | T | Hetero | W | GATGCCTAACAAGC-CAGGGGAA |  |
|  |  |  | I | GATGCCTAACAAGCTCAGGGGAA |  |
| R2 1 day-17 | T | Hetero | W | GATGCCTAACAAGC-CAGGGGAA |  |
|  |  |  | I | GATGCCTAACAAGCGCAGGGGAA |  |
| R2 1 day-19 | S | Hetero | W | GATGCCTAACAAGC-CAGGGGAG |  |
|  |  |  | I | GATGCCTAACAAGA-CAGGGGAG |  |
|  | | | | | |
| R2 2 day-1 | S | Hetero | W | GATGCCTAACAAGC-CAGGGGAG |  |
|  |  |  | I | GATGCCTAACAAGCACAGGGGAG |  |
|  | T | Hetero | W | GATGCCTAACAAGC-CAGGGGAA |  |
|  |  |  | I | GATGCCTAACAAGCTCAGGGGAA |  |
| R2 2 day-3 | S | Hetero | W | GATGCCTAACAAGC-CAGGGGAG |  |
|  |  |  | I | GATGCCTAACAAGCTCAGGGGAG |  |
| R2 2 day-4 | S | Homo | I | GATGCCTAACAAGCACAGGGGAG |  |
|  |  |  | I | GATGCCTAACAAGCACAGGGGAG |  |
|  | T | Homo | I | GATGCCTAACAAGCACAGGGGAA |  |
|  |  |  | I | GATGCCTAACAAGCACAGGGGAA |  |
| R2 2 day-7 | S | Hetero | W | GATGCCTAACAAGC-CAGGGGAG |  |
|  |  |  | I | GATGCCTAACAAGCACAGGGGAG |  |
| R2 2 day-9 | S | Hetero | W | GATGCCTAACAAGC-CAGGGGAG |  |
|  |  |  | I | GATGCCTAACAAGCACAGGGGAG |  |
| R2 2 day-11 | S | Homo | I | GATGCCTAACAAGCACAGGGGAG |  |
|  |  |  | I | GATGCCTAACAAGCACAGGGGAG |  |
|  | T | Hetero | W | GATGCCTAACAAGC-CAGGGGAA |  |
|  |  | Hetero | I | GATGCCTAACAAGCACAGGGGAA |  |
| R2 2 day-12 | S | Hetero | W | GATGCCTAACAAGC-CAGGGGAG |  |
|  |  | Hetero | I | GATGCCTAACAAGCTCAGGGGAG |  |
| R2 2 day-13 | S | Hetero | W | GATGCCTAACAAGC-CAGGGGAG |  |
|  |  | Hetero | I | GATGCCTAACAAGCACAGGGGAG |  |
| R2 2 day-16 | S | Hetero | W | GATGCCTAACAAGC-CAGGGGAG |  |
|  |  |  | I | GATGCCTAACAAGCACAGGGGAG |  |
| R2 2 day-20 | S | Hetero | W | GATGCCTAACAAGA-CAGGGGAG |  |
|  |  |  | I | GATGCCTAACAAGATCAGGGGAG |  |
|  | | | | | |
| R2 3 day-1 | S | Hetero | W | GATGCCTAACAAGC-CAGGGGAG |  |
|  |  |  | I | GATGCCTAACAAGCTCAGGGGAG |  |
| R2 3 day-2 | S | Hetero | I | GATGCCTAACAAGCACAGGGGAG |  |
|  |  |  | I | GATGCCTAACAAGCCCAGGGGAG |  |
| R2 3 day-3 | S | Hetero | I | GATGCCTAACAAGCACAGGGGAG |  |
|  |  |  | I | GATGCCTAACAAGCCCAGGGGAG |  |
| R2 3 day-4 | S | Hetero | W | GATGCCTAACAAGC-CAGGGGAG |  |
|  |  |  | I | GATGCCTAACAAGCTCAGGGGAG |  |
| R2 3 day-5 | S | Hetero | W | GATGCCTAACAAGC-CAGGGGAG |  |
|  |  |  | I | GATGCCTAACAAGCACAGGGGAG |  |
| R2 3 day-6 | S | Hetero | D | GATGCCTAACAAGC--TGGGGAG |  |
|  |  |  | I | GATGCCTAACAAGCACAGGGGAG |  |
|  | T | Hetero | I | GATGCCTAACAAGCACAGGGGAA |  |
|  |  |  | D | GATGCCTAACAAGC--AGGGGAA |  |
| R2 3 day-7 | S | Hetero | I | GATGCCTAACAAGCACAGGGGAG |  |
|  |  |  | I | GATGCCTAACAAGCTCAGGGGAG |  |
| R2 3 day-8 | S | Hetero | W | GATGCCTAACAAGC-CAGGGGAG |  |
|  |  |  | I | GATGCCTAACAAGCGCAGGGGAG |  |
| R2 3 day-9 | S | Hetero | I | GATGCCTAACAAGCACAGGGGAG |  |
|  |  |  | I | GATGCCTAACAAGCCCAGGGGAG |  |
| R2 3 day-10 | S | Hetero | W | GATGCCTAACAAGC-CAGGGGAG |  |
|  |  |  | I | GATGCCTAACAAGCACAGGGGAG |  |
| R2 3 day-11 | S | Hetero | W | GATGCCTAACAAGC-CAGGGGAG |  |
|  |  |  | I | GATGCCTAACAAGCACAGGGGAG |  |
| R2 3 day-12 | S | Hetero | W | GATGCCTAACAAGC-CAGGGGAG |  |
|  |  |  | I | GATGCCTAACAAGCACAGGGGAG |  |
| R2 3 day-13 | S | Hetero | I | GATGCCTAACAAGCACAGGGGAG |  |
|  |  |  | I | GATGCCTAACAAGCCCAGGGGAG |  |
| R2 3 day-14 | S | Hetero | W | GATGCCTAACAAGC-CAGGGGAG |  |
|  |  |  | I | GATGCCTAACAAGCACAGGGGAG |  |
| R2 3 day-15 | S | Hetero | W | GATGCCTAACAAGC-CAGGGGAG |  |
|  |  |  | I | GATGCCTAACAAGCTCAGGGGAG |  |
|  | T | Hetero | W | GATGCCTAACAAGC-CAGGGGAA |  |
|  |  |  | I | GATGCCTAACAAGCGCAGGGGAA |  |
| R2 3 day-16 | S | Hetero | W | GATGCCTAACAAGC-CAGGGGAG |  |
|  |  |  | I | GATGCCTAACAAGCACAGGGGAG |  |
| R2 3 day-17 | S | Hetero | I | GATGCCTAACAAGCACAGGGGAG |  |
|  |  |  | I | GATGCCTAACAAGCCCAGGGGAG |  |
|  | T | Homo | I | GATGCCTAACAAGCACAGGGGAA |  |
|  |  |  | I | GATGCCTAACAAGCACAGGGGAA |  |
| R2 3 day-18 | S | Hetero | I | GATGCCTAACAAGCACAGGGGAG |  |
|  |  |  | I | GATGCCTAACAAGCCCAGGGGAG |  |
| R2 3 day-19 | S | Hetero | W | GATGCCTAACAAGC-CAGGGGAG |  |
|  |  |  | I | GATGCCTAACAAGCACAGGGGAG |  |
| R2 3 day-20 | S | Hetero | W | GATGCCTAACAAGC-CAGGGGAG |  |
|  |  |  | I | GATGCCTAACAAGCACAGGGGAG |  |
|  | | | | | |
| R2 4 day-6 | S | Hetero | W | GATGCCTAACAAGC-CAGGGGAG |  |
|  |  |  | I | GATGCCTAACAAGCACAGGGGAG |  |
|  | T | Hetero | W | GATGCCTAACAAGC-CAGGGGAA |  |
|  |  |  | I | GATGCCTAACAAGCTCAGGGGAA |  |
| R2 4 day-10 | S | Hetero | W | GATGCCTAACAAGC-CAGGGGAG |  |
|  |  |  | I | GATGCCTAACAAGCTCAGGGGAG |  |
| R2 4 day-11 | S | Hetero | W | GATGCCTAACAAGC-CAGGGGAG |  |
|  |  |  | I | GATGCCTAACAAGCTCAGGGGAG |  |
| R2 4 day-13 | S | Hetero | W | GATGCCTAACAAGC-CAGGGGAG |  |
|  |  |  | I | GATGCCTAACAAGCTCAGGGGAG |  |
| R2 4 day-14 | S | Hetero | W | GATGCCTAACAAGC-CAGGGGAG |  |
|  |  |  | I | GATGCCTAACAAGCTCAGGGGAG |  |
| R2 4 day-15 | S | Hetero | W | GATGCCTAACAAGC-CAGGGGAG |  |
|  |  |  | I | GATGCCTAACAAGCTCAGGGGAG |  |
| R2 4 day-16 | T | Hetero | W | GATGCCTAACAAGC-CAGGGGAA |  |
|  |  |  | I | GATGCCTAACAAGCGCAGGGGAA |  |
| R2 4 day-18 | T | Hetero | I | GATGCCTAACAAGCTCAGGGGAA |  |
|  |  |  | D | GATGCCTAACAAGC-(-17 bps) |  |
| R2 4 day-20 | S | Homo | I | GATGCCTAACAAGCACAGGGGAG |  |
|  |  |  | I | GATGCCTAACAAGCACAGGGGAG |  |

**Experiment 3**

| **Sample no.** | **Genome type** | **genotype** | **Mutation type** | ***NtPDS* sequences** |  |
| --- | --- | --- | --- | --- | --- |
| Wild type | S |  | W | GATGCCTAACAAGC-CAGGGGAG |  |
|  | T |  | W | GATGCCTAACAAGC-CAGGGGAA |  |
|  | | | | | |
| R3 0 day-19 | S | Hetero | W | GATGCCTAACAAGC-CAGGGGAG |  |
|  |  |  | I | GATGCCTAACAAGA-CAGGGGAG |  |
|  | | | | | |
| R3 1 day-1 | T | Homo | I | GATGCCTAACAAGCGCAGGGGAG |  |
|  |  |  | I | GATGCCTAACAAGCGCAGGGGAG |  |
| R3 1 day-2 | S | Homo | I | GATGCCTAACAAGCGCAGGGGAG |  |
|  |  |  | I | GATGCCTAACAAGCGCAGGGGAG |  |
| R3 1 day-6 | S | Homo | I | GATGCCTAACAAGCTCAGGGGAG |  |
|  |  |  | I | GATGCCTAACAAGCTCAGGGGAG |  |
|  | T | Homo | I | GATGCCTAACAAGCTCAGGGGAG |  |
|  |  |  | I | GATGCCTAACAAGCTCAGGGGAG |  |
| R3 1 day-8 | T | Hetero | D | GATGCCTAACAAGC--AGGGGAA |  |
|  |  |  | D | GATGCCTAACAAGA--AGGGGAA |  |
| R3 1 day-15 | S | Homo | I | GATGCCTAACAAGCACAGGGGAG |  |
|  |  |  | I | GATGCCTAACAAGCACAGGGGAG |  |
|  | T | Homo | I | GATGCCTAACAAGCACAGGGGAG |  |
|  |  |  | I | GATGCCTAACAAGCACAGGGGAG |  |
|  | | | | | |
| R3 2 day-2 | S | Hetero | D | GATGCCTAACA---TCAGGGGAG |  |
|  |  | Hetero | D | GATGCCTAACA---TCAGGGGAG |  |
| R3 2 day-3 | S | Hetero | W | GATGCCTAACAAGC-CAGGGGAG |  |
|  |  |  | I | GATGCCTAACAAGCGCAGGGGAG |  |
|  | T | Homo | I | GATGCCTAACAAGCGCAGGGGAA |  |
|  |  |  | I | GATGCCTAACAAGCGCAGGGGAA |  |
| R3 2 day-5 | T | Homo | I | GATGCCTAACAAGCGCAGGGGAA |  |
|  |  |  | I | GATGCCTAACAAGCGCAGGGGAA |  |
| R3 2 day-7 | S | Hetero | D | GATGCCTAACAAGC--CAGGGAG |  |
|  |  |  | I | GATGCCTAACAAGCTCAGGGGAG |  |
|  | T | Homo | D | GATGCCTAACAAGC--AGGGGAA |  |
|  |  |  | D | GATGCCTAACAAGC--AGGGGAA |  |
| R3 2 day-11 | S | Hetero | W | GATGCCTAACAAGC-CAGGGGAG |  |
|  |  |  | I | GATGCCTAACAAGCTCAGGGGAG |  |
| R3 2 day-15 | S | Homo | I | GATGCCTAACAAGCACAGGGGAG |  |
|  |  |  | I | GATGCCTAACAAGCACAGGGGAG |  |
| R3 2 day-16 | S | Homo | I | GATGCCTAACAAGCACAGGGGAG |  |
|  |  |  | I | GATGCCTAACAAGCACAGGGGAG |  |
| R3 2 day-18 | S | Homo | I | GATGCCTAACAAGCTCAGGGGAG |  |
|  |  |  | I | GATGCCTAACAAGCTCAGGGGAG |  |
|  | | | | | |
| R3 3 day-1 | S | Hetero | W | GATGCCTAACAAGC-CAGGGGAG |  |
|  |  |  | I | GATGCCTAACAAGCACAGGGGAG |  |
| R3 3 day-3 | S | Hetero | W | GATGCCTAACAAGC-CAGGGGAG |  |
|  |  |  | D | GATGCCTAACAAGC-(-71 bps) |  |
|  | T | Hetero | W | GATGCCTAACAAGC-CAGGGGAA |  |
|  |  |  | D | GATGCCTAACA-(11 bps) |  |
| R3 3 day-4 | S | Homo | I | GATGCCTAACAAGCTCAGGGGAG |  |
|  |  |  | I | GATGCCTAACAAGCTCAGGGGAG |  |
|  | T | Homo | I | GATGCCTAACAAGCTCAGGGGAA |  |
|  |  |  | I | GATGCCTAACAAGCTCAGGGGAA |  |
| R3 3 day-5 | S | Hetero | W | GATGCCTAACAAGC-CAGGGGAG |  |
|  |  |  | I | GATGCCTAACAAGCACAGGGGAG |  |
| R3 3 day-7 | T | Hetero | W | GATGCCTAACAAGC-CAGGGGAA |  |
|  |  |  | I | GATGCCTAACAAGCACAGGGGAA |  |
| R3 3 day-8 | T | Hetero | W | GATGCCTAACAAGC-CAGGGGAA |  |
|  |  |  | I | GATGCCTAACAAGCACAGGGGAA |  |
| R3 3 day-9 | S | Homo | I | GATGCCTAACAAGCTCAGGGGAG |  |
|  |  |  | I | GATGCCTAACAAGCTCAGGGGAG |  |
|  | T | Homo | D | GATGCC--------(-26 bps) |  |
|  |  |  | D | GATGCC--------(-26 bps) |  |
| R3 3 day-12 | S | Homo | I | GATGCCTAACAAGCACAGGGGAG |  |
|  |  |  | I | GATGCCTAACAAGCACAGGGGAG |  |
| R3 3 day-13 | S | Hetero | W | GATGCCTAACAAGC-CAGGGGAG |  |
|  |  |  | I | GATGCCTAACAAGCTCAGGGGAG |  |
|  | T | Hetero | W | GATGCCTAACAAGC-CAGGGGAA |  |
|  |  |  | I | GATGCCTAACAAGCCAAGGGGAA |  |
| R3 3 day-15 | S | Homo | I | GATGCCTAACAAGCTCAGGGGAG |  |
|  |  |  | I | GATGCCTAACAAGCTCAGGGGAG |  |
| R3 3 day-16 | S | Homo | I | GATGCCTAACAAGCTCAGGGGAG |  |
|  |  |  | I | GATGCCTAACAAGCTCAGGGGAG |  |
| R3 3 day-18 | S | Homo | D | GATGCCTAA------CAGGGGAG |  |
|  |  |  | D | GATGCCTAA------CAGGGGAG |  |
|  | | | | | |
| R3 4 day-1 | S | Homo | I | GATGCCTAACAAGCGCAGGGGAG |  |
|  |  |  | I | GATGCCTAACAAGCGCAGGGGAG |  |
| R3 4 day-3 | S | Homo | I | GATGCCTAACAAGCTCAGGGGAG |  |
|  |  |  | I | GATGCCTAACAAGCTCAGGGGAG |  |
|  | T | Homo | I | GATGCCTAACAAGCACAGGGGAA |  |
|  |  |  | I | GATGCCTAACAAGCACAGGGGAA |  |
| R3 4 day-6 | T | Hetero | W | GATGCCTAACAAGC-CAGGGGAA |  |
|  |  |  | I | GATGCCTAACAAGCTCAGGGGAA |  |
| R3 4 day-7 | S | Homo | I | GATGCCTAACAAGCTCAGGGGAG |  |
|  |  |  | I | GATGCCTAACAAGCTCAGGGGAG |  |
|  | T | Hetero | W | GATGCCTAACAAGC-CAGGGGAA |  |
|  |  |  | I | GATGCCTAACAAGC-CAG(+5 bps) |  |
| R3 4 day-8 | S | Hetero | D | GATGCCTAACA------GGGGAG |  |
|  |  |  | I | GATGCCTAACAAGCTCGGGGAAG |  |
| R3 4 day-16 | S | Hetero | W | GATGCCTAACAAGC-CAGGGGAG |  |
|  |  |  | I | GATGCCTAACAAGCTCAGGGGAG |  |
| R3 4 day-17 | S | Homo | I | GATGCCTAACAAGCTCAGGGGAG |  |
|  |  |  | I | GATGCCTAACAAGCTCAGGGGAG |  |
