# Supplementary material for: Application of protoplast technology to CRISPR/Cas9 mutagenesis: from single‐cell mutation detection to mutant plant regeneration
Source: Plant Biotechnol J. 2018 Jan 10;16(7):1295–310. doi: 10.1111/pbi.12870 (PMC5999315; doi:10.1111/pbi.12870)
Supplement: Supplementary file 5 — Data S3 The sequences of Zea mays single‐cell ZmIPK genes in Figure 5. [file PBI-16-1295-s002.docx]

**Supplemental Data 3. The sequences of *Zea mays* single cell *ZmIPK* genes in Figure 5.**

>no._1

CCGGGGCCTCGTGGCGTAGCCCGGGAGTAGTTGATGTCAATGACGAGATAGCGGTCGCCGGCGCGGACGTCGCGGATCATGTCGAAGTTGAAGAGTTGCAGGCCCAGCGCGCGGCGGAGGCCGCCCGCGATCTGGTTGATGAATGCGGCGGGCGGCACGACGGCGTCCTCGAGACTCTTTTCGCCGTAGTACTCCTCCGCCGTGCGCTCAGTGGGGAGGTTGGAGACCTGGGAGAAGGAGACGGATGCTCGGAGACGACCTGGAGCATGGAGATGCGGTTGTGGAGGCGGTCGATGGCGTGGGGCGGGTCGACGATGGGGACGGCCGGGTGGCGCGCGGCGAAGGCCACGAGCTGGGCGCGCCAGTCGTCTCCGTAGAGCTTGTGA

>no._4

CGGTCTCTTGGCGTAGCCCGGGAGTAGTTGATGTCAATGACGAGATAGCGGTCGCCGGCGCGGACGTCGCGGATCATGTCGAAGTTGAAGAGTTGCAGGCCCAGCGCGCGGCGGAGGCCGCCCGCGATCTGGTTGATGAATGCGGCGGGCGGCACGACGGCGTCCTCGAGACTCTTTTCGCCGTAGTACTCCTCCGCCGTGCGCTCAGTGGGGAGGTTGGAGACCTGGGAGAAGGAGACGGATGCTCGGAGACGACCTGGAGCATGGAGATGCGGTTGTGGAGGCGGTCGATGGCGTGGGGCGGGTCGACGATGGGGACGGCCGGGTGGCGCGCGGCGAAGGCCACGAGCTGGGCGCGCCAGTCGTCTCCGAGG

>no._13

ACGTAATCTTGTGGGACTTGGCGGTGCCGTCGGCGACGAGGGGCTTGGCGATGAGCGGGAAGCGGAGCGCGGCAAGGAGTCCGAAGTCGGCGAGCGCGGCAGCGTCGTAGACGACGACCTGGCTGGGGATACCGAAAGTGCTGTCCTGGTCGGCGGCGTGGTCGAGCTCGGAGACGACCTGGAGCATGGAGATGCGGTTGTGGAGGCGGTCGATGGCGTGGGGCGGGTCGACGATGGGGACGGCCGGGTGGCGCGCGGCGAAGGCCACGAGCTGGGCGCGCCAGTCGTCTCCGTAGAGCTTGTGA

>no._16

TCGGGGCTCTTGGCGTAGCCCGGGAGTAGTTGATGTCAATGACGAGATAGCGGTCGCCGGCGCGGACGTCGCGGATCATGTCGAAGTTGAAGAGTTGCAGGCCCAGCGCGCGGCGGAGGCCGCCCGCGATCTGGTTGATGAATGCGGCGGACGGCACGACGGCGTCCTCGAGACTCTTTTCGCCGTAGTACTCCTCCGCCGTGCGCTCAGTGGGGAGGTTGGAGACCTGGGAGAAGGAGACGGATAGCTCGGAGACGACCTGGAGCATGGAGATGCGGTTGTGGAGGCGGTCGATGGCGTGGGGCGGGTCGACGATGGGGACGGCCGGGTGGCGCGCGGCGAAGGCCACGAGCTGGGCGCGCCAGTCGTCTCCGTGAAGGCTTGTGA

>no._18

CCGGTCTTCTTGGCGTAGCCCGGGAGTAGTTGATGTCAATGACGAGATAGCGGTCGCCGGCGCGGACGTCGCGGATCATGTCGAAGTTGAAGAGTTGCAGGCCCAGCGCGCGGCGGAGGCCGCCCGCGATCTGGTTGATGAATGCGGCGGGCGGCACGACGGCGTCCTCGAGACTCTTTTCGCCGTAGTACTCCTCCGCCGTGCGCTCAGTGGGGAGGTTGGAGACCTGGGAGAAGGAGACGGATGCTCGGAGACGACCTGGAGCATGGAGATGCGGTTGTGGAGGCGGTCGATGGCGTGGGGCGGGTCGACGATGGGGACGGCCGGGTGGCGCGCGGCGAAGGCCACGAGCTGGGCGCGCCAGTCGTCTCCGTGAAGCTTGTGA
