# Supplementary material for: Application of protoplast technology to CRISPR/Cas9 mutagenesis: from single‐cell mutation detection to mutant plant regeneration
Source: Plant Biotechnol J. 2018 Jan 10;16(7):1295–310. doi: 10.1111/pbi.12870 (PMC5999315; doi:10.1111/pbi.12870)
Supplement: Supplementary file 6 — Data S4 The NtPDS sequences of Figure 7 (Exp2R1) and Table 4. [file PBI-16-1295-s003.docx]

**Supplemental Data 4. The *NtPDS* sequences of Figure 7 (Exp2R1) and Table 4.** Red box in Sample no. column indicates all four copies of the *NtPDS* gene have the same mutations. S, *N. sylvestris* form; T, *N. tomentosiformis* form. Orange box-Homo, homozygous in this form; Light blue box-hetero, heterozygous in this form. W, wild-type; I, insertion; D, deletion; Letter in red, the mutated nucleotide.

| **Sample no.** | **Genome type** | **genotype** | **Mutation type** | ***NtPDS* sequences** |  |
| --- | --- | --- | --- | --- | --- |
| Wild type | S |  |  | GATGCCTAACAAGC-CAGGGGAG |  |
|  | T |  |  | GATGCCTAACAAGC-CAGGGGAA |  |
|  | | | | | |
| Exp1R1A1 | S | Hetero | D | GATGCCTAACAAGC-(-14 bps) |  |
|  |  |  | I | GATGCCTAACAAGCTCAGGGGAG |  |
|  | T | Hetero | I | GATGCCTAACAAGCTCAGGGGAA |  |
|  |  |  | I | GATGCCTAACAAGCACAGGGGAA |  |
| Exp1R1A2 | S | Hetero | I | GATGCCTAACAAGCACAGGGGAG |  |
|  |  |  | I | GATGCCTAACAAGCTCAGGGGAG |  |
|  | T | Homo | I | GATGCCTAACAAGCTCAGGGGAA |  |
|  |  |  | I | GATGCCTAACAAGCTCAGGGGAA |  |
| Exp1R1A3 | S | Homo | I | GATGCCTAACAAGCTCAGGGGAG |  |
|  |  |  | I | GATGCCTAACAAGCTCAGGGGAG |  |
|  | T | Homo | I | GATGCCTAACAAGCGCAGGGGAA |  |
|  |  |  | I | GATGCCTAACAAGCGCAGGGGAA |  |
| Exp1R1A4 | S | Homo | I | GATGCCTAACAAGCGCAGGGGAG |  |
|  |  |  | I | GATGCCTAACAAGCGCAGGGGAG |  |
|  | T | Homo | I | GATGCCTAACAAGCGCAGGGGAA |  |
|  |  |  | I | GATGCCTAACAAGCGCAGGGGAA |  |
| Exp1R1A5 | S | Homo | I | GATGCCTAACAAGCACAGGGGAG |  |
|  |  |  | I | GATGCCTAACAAGCACAGGGGAG |  |
|  | T | Homo | I | GATGCCTAACAAGCTCAGGGGAA |  |
|  |  |  | I | GATGCCTAACAAGCTCAGGGGAA |  |
| Exp1R1A6 | S | Homo | I | GATGCCTAACAAGCTCAGGGGAG |  |
|  |  |  | I | GATGCCTAACAAGCTCAGGGGAG |  |
|  | T | Homo | I | GATGCCTAACAAGCTCAGGGGAG |  |
|  |  |  | I | GATGCCTAACAAGCTCAGGGGAG |  |
| Exp1R1A7 | S | Hetero | I | GATGCCTAACAAGCACAGGGAAA |  |
|  |  |  | I | GATGCCTAACAAGCTCAGGGAAA |  |
|  | T | Homo | I | GATGCCTAACAAGCTCAGGGGAA |  |
|  |  |  | I | GATGCCTAACAAGCTCAGGGGAA |  |
| Exp1R1A8 | S | Hetero | I | GATGCCTAACAAGCTCAGGGGAG |  |
|  |  |  | I | GATGCCTAACAAGCACAGGGGAG |  |
|  | T | Hetero | I | GATGCCTAACAAGCTCAGGGGAA |  |
|  |  |  | I | GATGCCTAACAAGCACAGGGGAA |  |
| Exp1R1A9 | S | Hetero | I | GATGCCTAACAAGCTCAGGGGAG |  |
|  |  |  | I | GATGCCTAACAAGCACAGGGGAG |  |
|  | T | Hetero | I | GATGCCTAACAAGCTCAGGGGAA |  |
|  |  |  | I | GATGCCTAACAAGCACAGGGGAA |  |
| Exp1R1A10 | S | Homo | I | GATGCCTAACAAGCGCAGGGGAG |  |
|  |  |  | I | GATGCCTAACAAGCGCAGGGGAG |  |
|  | T | Homo | I | GATGCCTAACAAGCTCAGGGGAA |  |
|  |  |  | I | GATGCCTAACAAGCTCAGGGGAA |  |
| Exp1R1A11 | S | Homo | I | GATGCCTAACAAGCTCAGGGGAG |  |
|  |  |  | I | GATGCCTAACAAGCTCAGGGGAG |  |
|  | T | Homo | I | GATGCCTAACAAGCACAGGGGAA |  |
|  |  |  | I | GATGCCTAACAAGCACAGGGGAA |  |
| Exp1R1A12 | S | Hetero | I | GATGCCTAACAAGCGCAGGGGAG |  |
|  |  |  | I | GATGCCTAACAAGCTCAGGGGAG |  |
|  | T | Homo | I | GATGCCTAACAAGCTCAGGGGAA |  |
|  |  |  | I | GATGCCTAACAAGCTCAGGGGAA |  |
| Exp1R1A13 | S | Homo | I | GATGCCTAACAAGCTCAGGGGAG |  |
|  |  |  | I | GATGCCTAACAAGCTCAGGGGAG |  |
|  | T | Hetero | I | GATGCCTAACAAGCTCAGGGGAA |  |
|  |  |  | I | GATGCCTAACAAGCACAGGGGAA |  |
| Exp1R1A14 | S | Homo | I | GATGCCTAACAAGCACAGGGGAG |  |
|  |  |  | I | GATGCCTAACAAGCACAGGGGAG |  |
|  | T | Homo | I | GATGCCTAACAAGCGCAGGGGAA |  |
|  |  |  | I | GATGCCTAACAAGCGCAGGGGAA |  |
| Exp1R1A15 | S | Hetero | I | GATGCCTAACAAGCTCAGGGGAG |  |
|  |  |  | D | GATGCCTAACAAGC--AGGGGAG |  |
|  | T | Homo | D | GATGCCTAACAAGC--AGGGGAA |  |
|  |  |  | D | GATGCCTAACAAGC--AGGGGAA |  |
| Exp1R1A16 | S | Homo | I | GATGCCTAACAAGCACAGGGGAG |  |
|  |  |  | I | GATGCCTAACAAGCACAGGGGAG |  |
|  | T | Hetero | I | GATGCCTAACAAGCGCAGGGGAA |  |
|  |  |  | I | GATGCCTAACAAGCACAGGGGAA |  |
| Exp1R1A17 | S | Hetero | I | GATGCCTAACAAGCTCAGGGGAG |  |
|  |  |  | I | GATGCCTAACAAGCACAGGGGAG |  |
|  | T | Homo | I | GATGCCTAACAAGCGCTGGGGAA |  |
|  |  |  | I | GATGCCTAACAAGCGCTGGGGAA |  |
| Exp1R1A18 | S | Homo | I | GATGCCTAACAAGCTCAGGGGAG |  |
|  |  |  | I | GATGCCTAACAAGCTCAGGGGAG |  |
|  | T | Homo | I | GATGCCTAACAAGCGCTGGGGAA |  |
|  |  |  | I | GATGCCTAACAAGCGCTGGGGAA |  |
| Exp1R1A19 | S | Hetero | I | GATGCCTAACAAGCTCAGGGGAG |  |
|  |  |  | D | GATGCCTAACCAG-----GGGAG |  |
|  | T | Hetero | I | GATGCCTAACAAGCCCAGGGGAA |  |
|  |  |  | I | GATGCCTAACAAGCTCAGGGGAA |  |
| Exp1R1A20 | S | Hetero | I | GATGCCTAACAAGCTCAGGGGAG |  |
|  |  |  | I | GATGCCTAACAAGCACAGGGGAG |  |
|  | T | Hetero | D | GATGCCTAACAAGC--CAGGGAA |  |
|  |  |  | I | GATGCCTAACAAGCACAGGGGAA |  |
|  | | | | | |
| Exp1R1G1 | S | Hetero | W | GATGCCTAACAAGC-CAGGGGAG |  |
|  |  |  | I | GATGCCTAACAAGCCCAGGGGAA |  |
|  | T |  | W | GATGCCTAACAAGC-CAGGGGAA |  |
|  |  |  | W | GATGCCTAACAAGC-CAGGGGAA |  |
| Exp1R1G2 | S | Homo | I | GATGCCTAACCAGGTCAGGGGAG |  |
|  |  |  | I | GATGCCTAACCAGGTCAGGGGAG |  |
|  | T | Hetero | W | GATGCCTAACAAGC-CAGGGGAA |  |
|  |  |  | D | GATGCCTAACAAGC--AGGGGAA |  |
| Exp1R1G3 | S |  | W | GATGCCTAACAAGC-CAGGGGAG |  |
|  |  |  | W | GATGCCTAACAAGC-CAGGGGAG |  |
|  | T |  | W | GATGCCTAACAAGC-CAGGGGAA |  |
|  |  |  | W | GATGCCTAACAAGC-CAGGGGAA |  |
| Exp1R1G4 | S | Hetero | W | GATGCCTAACAAGC-CAGGGGAG |  |
|  |  |  | I | GATGCCTAACAAGCACAGGGGAG |  |
|  | T |  | W | GATGCCTAACAAGC-CAGGGGAA |  |
|  |  |  | W | GATGCCTAACAAGC-CAGGGGAA |  |
| Exp1R1G5 | S |  | W | GATGCCTAACAAGC-CAGGGGAG |  |
|  |  |  | W | GATGCCTAACAAGC-CAGGGGAG |  |
|  | T |  | W | GATGCCTAACAAGC-CAGGGGAA |  |
|  |  |  | W | GATGCCTAACAAGC-CAGGGGAA |  |
| Exp1R1G6 | S |  | W | GATGCCTAACAAGC-CAGGGGAG |  |
|  |  |  | W | GATGCCTAACAAGC-CAGGGGAG |  |
|  | T |  | W | GATGCCTAACAAGC-CAGGGGAA |  |
|  |  |  | W | GATGCCTAACAAGC-CAGGGGAA |  |
| Exp1R1G7 | S |  | W | GATGCCTAACAAGC-CAGGGGAG |  |
|  |  |  | W | GATGCCTAACAAGC-CAGGGGAG |  |
|  | T |  | W | GATGCCTAACAAGC-CAGGGGAA |  |
|  |  |  | W | GATGCCTAACAAGC-CAGGGGAA |  |
| Exp1R1G8 | S |  | W | GATGCCTAACAAGC-CAGGGGAG |  |
|  |  |  | W | GATGCCTAACAAGC-CAGGGGAG |  |
|  | T |  | W | GATGCCTAACAAGC-CAGGGGAA |  |
|  |  |  | W | GATGCCTAACAAGC-CAGGGGAA |  |
| Exp1R1G9 | S |  | W | GATGCCTAACAAGC-CAGGGGAG |  |
|  |  |  | W | GATGCCTAACAAGC-CAGGGGAG |  |
|  | T |  | W | GATGCCTAACAAGC-CAGGGGAA |  |
|  |  |  | W | GATGCCTAACAAGC-CAGGGGAA |  |
| Exp1R1G10 | S | Hetero | W | GATGCCTAACAAGC-CAGGGGAG |  |
|  |  |  | I | GATGCCTAACAAGCTCAGGGGAG |  |
|  | T |  | W | GATGCCTAACAAGC-CAGGGGAA |  |
|  |  |  | W | GATGCCTAACAAGC-CAGGGGAA |  |
| Exp1R1G11 | S |  | W | GATGCCTAACAAGC-CAGGGGAG |  |
|  |  |  | W | GATGCCTAACAAGC-CAGGGGAA |  |
|  | T |  | W | GATGCCTAACAAGC-CAGGGGAA |  |
|  |  |  | W | GATGCCTAACAAGC-CAGGGGAA |  |
| Exp1R1G12 | S | Hetero | I | GATGCCTAACAAGCACAGGGGAA |  |
|  |  |  | I | GATGCCTAACAAGCGCAGGGGAA |  |
|  | T | Homo | D | GATGCCTAACA----CAGGGGAA |  |
|  |  |  | D | GATGCCTAACA----CAGGGGAA |  |
| Exp1R1G13 | S |  | W | GATGCCTAACAAGC-CAGGGGAG |  |
|  |  |  | W | GATGCCTAACAAGC-CAGGGGAG |  |
|  | T |  | W | GATGCCTAACAAGC-CAGGGGAA |  |
|  |  |  | W | GATGCCTAACAAGC-CAGGGGAA |  |
| Exp1R1G14 | S |  | W | GATGCCTAACAAGC-CAGGGGAG |  |
|  |  |  | W | GATGCCTAACAAGC-CAGGGGAG |  |
|  | T |  | W | GATGCCTAACAAGC-CAGGGGAA |  |
|  |  |  | W | GATGCCTAACAAGC-CAGGGGAA |  |
| Exp1R1G15 | S |  | W | GATGCCTAACAAGC-CAGGGGAG |  |
|  |  |  | W | GATGCCTAACAAGC-CAGGGGAG |  |
|  | T |  | W | GATGCCTAACAAGC-CAGGGGAA |  |
|  |  |  | W | GATGCCTAACAAGC-CAGGGGAA |  |
| Exp1R1G16 | S |  | W | GATGCCTAACAAGC-CAGGGGAG |  |
|  |  |  | W | GATGCCTAACAAGC-CAGGGGAG |  |
|  | T |  | W | GATGCCTAACAAGC-CAGGGGAA |  |
|  |  |  | W | GATGCCTAACAAGC-CAGGGGAA |  |
| Exp1R1G17 | S |  | W | GATGCCTAACAAGC-CAGGGGAG |  |
|  |  |  | W | GATGCCTAACAAGC-CAGGGGAG |  |
|  | T |  | W | GATGCCTAACAAGC-CAGGGGAA |  |
|  |  |  | W | GATGCCTAACAAGC-CAGGGGAA |  |
| Exp1R1G18 | S | Hetero | W | GATGCCTAACAAGC-CAGGGGAG |  |
|  |  |  | I | GATGCCTAACAAGCTCAGGGGAG |  |
|  | T |  | W | GATGCCTAACAAGC-CAGGGGAA |  |
|  |  |  | W | GATGCCTAACAAGC-CAGGGGAA |  |
| Exp1R1G19 | S | Homo | W | GATGCCTAACAAGCGCAGGGGAG |  |
|  |  |  | W | GATGCCTAACAAGCGCAGGGGAG |  |
|  | T | Hetero | W | GATGCCTAACAAGC-CAGGGGAA |  |
|  |  | Hetero | I | GATGCCTAACAAGCGCAGGGGAA |  |
| Exp1R1G20 | S |  | W | GATGCCTAACAAGC-CAGGGGAA |  |
|  |  |  | W | GATGCCTAACAAGC-CAGGGGAG |  |
|  | T |  | W | GATGCCTAACAAGC-CAGGGGAA |  |
|  |  |  | W | GATGCCTAACAAGC-CAGGGGAA |  |

| **Sample no.** | **Genome type** | **genotype** | **Mutation type** | ***NtPDS* sequences** |  |
| --- | --- | --- | --- | --- | --- |
| Wild type | S |  | W | GATGCCTAACAAGC-CAGGGGAG |  |
|  | T |  | W | GATGCCTAACAAGC-CAGGGGAA |  |
|  | | | | | |
| Exp1R2A1 | S | Hetero | I | GATGCCTAACAAGCTCAGGGGAG |  |
|  |  |  | I | GATGCCTAACAAGCGGCAGGGGA |  |
|  | T | Hetero | I | GATGCCTAACAAGCACAGGGGAA |  |
|  |  |  | I | GATGCCTAACAAGCTCAGGGGAA |  |
| Exp1R2A2 | S | Hetero | I | GATGCCTAACAAGCACAGGGGAG |  |
|  |  |  | D | GATGCCTAACAAGC—-AGGGGAG |  |
|  | T | Homo | I | GATGCCTAACAAGCTCAGGGGAA |  |
|  |  |  | I | GATGCCTAACAAGCTCAGGGGAA |  |
| Exp1R2A3 | S | Hetero | I | GATGCCTAACAAGCTCAGGGGAG |  |
|  |  |  | I | GATGCCTAACAAGCGCAGGGGAG |  |
|  | T | Homo | D | GATGCCTAACAA---CAGGGGAA |  |
|  |  |  | D | GATGCCTAACAA---CAGGGGAA |  |
| Exp1R2A4 | S | Hetero | I | GATGCCTAACAAGCTCAGGGGAG |  |
|  |  |  | I | GATGCCTAACAAGCACAGGGGAG |  |
|  | T | Homo | I | GATGCCTAACAAGCTCAGGGGAA |  |
|  |  |  | I | GATGCCTAACAAGCTCAGGGGAA |  |
| Exp1R2A5 | S | Homo | I | GATGCCTAACAAGCTCAGGGGAG |  |
|  |  |  | I | GATGCCTAACAAGCTCAGGGGAG |  |
|  | T | Hetero | I | GATGCCTAACAAGCGCAGGGGAA |  |
|  |  |  | I | GATGCCTAACAAGCTCAGGGGAA |  |
| Exp1R2A6 | S | Hetero | I | GATGCCTAACAAGCTCAGGGGAG |  |
|  |  |  | I | GATGCCTAACAAGCGCAGGGGAG |  |
|  | T | Homo | I | GATGCCTAACAAGCTCAGGGGAA |  |
|  |  |  | I | GATGCCTAACAAGCTCAGGGGAA |  |
| Exp1R2A7 | S | Hetero | I | GATGCCTAACAAGCACAGGGGAG |  |
|  |  |  | I | GATGCCTAACAAGCTCAGGGGAG |  |
|  | T | Hetero | I | GATGCCTAACAAGCACAGGGGAA |  |
|  |  |  | D | GATGCCTAACAAGC—-AGGGGAA |  |
| Exp1R2A8 | S | Hetero | I | GATGCCTAACAAGCACAGGGGAG |  |
|  |  |  | I | GATGCCTAACAAGCTCAGGGGAG |  |
|  | T | Homo | I | GATGCCTAACAAGCTCAGGGGAA |  |
|  |  |  | I | GATGCCTAACAAGCTCAGGGGAA |  |
| Exp1R2A9 | S | Homo | I | GATGCCTAACAAGCTCAGGGGAG |  |
|  |  |  | I | GATGCCTAACAAGCTCAGGGGAG |  |
|  | T | Homo | I | GATGCCTAACAAGCGCAGGGGAA |  |
|  |  |  | I | GATGCCTAACAAGCGCAGGGGAA |  |
| Exp1R2A10 | S | Hetero | I | GATGCCTAACAAGCTCAGGGGAG |  |
|  |  |  | I | GATGCCTAACAAGCGCAGGGGAG |  |
|  | T | Homo | I | GATGCCTAACAAGCTCAGGGGAA |  |
|  |  |  | I | GATGCCTAACAAGCTCAGGGGAA |  |
| Exp1R2A11 | S | Homo | I | GATGCCTAACAAGCTCAGGGGAG |  |
|  |  |  | I | GATGCCTAACAAGCTCAGGGGAG |  |
|  | T | Homo | I | GATGCCTAACAAGCTCAGGGGAA |  |
|  |  |  | I | GATGCCTAACAAGCTCAGGGGAA |  |
| Exp1R2A12 | S | Homo | I | GATGCCTAACAAGCTCAGGGGAG |  |
|  |  |  | I | GATGCCTAACAAGCTCAGGGGAG |  |
|  | T | Homo | I | GATGCCTAACAAGCGCAGGGGAA |  |
|  |  |  | I | GATGCCTAACAAGCGCAGGGGAA |  |
| Exp1R2A13 | S | Homo | I | GATGCCTAACAAGCTCAGGGGAG |  |
|  |  |  | I | GATGCCTAACAAGCTCAGGGGAG |  |
|  | T | Homo | I | GATGCCTAACAAGCTCAGGGGAA |  |
|  |  |  | I | GATGCCTAACAAGCTCAGGGGAA |  |
| Exp1R2A14 | S | Homo | I | GATGCCTAACAAGCACAGGGGAG |  |
|  |  |  | I | GATGCCTAACAAGCACAGGGGAG |  |
|  | T | Homo | I | GATGCCTAACAAGCACAGGGGAA |  |
|  |  |  | I | GATGCCTAACAAGCACAGGGGAA |  |
| Exp1R2A15 | S | Hetero | I | GATGCCTAACAAGCACAGGGGAG |  |
|  |  |  | I | GATGCCTAACAAGCTCAGGGGAG |  |
|  | T | Hetero | D | GATGCCTAACAAG---AGGGGAA |  |
|  |  |  | I | GATGCCTAACAAGCACAGGGGAA |  |
| Exp1R2A16 | S | Hetero | I | GATGCCTAACAAGCGCAGGGGAG |  |
|  |  |  | I | GATGCCTAACAAGCGGCAGGGGA |  |
|  | T | Hetero | I | GATGCCTAACAAGCACAGGGGAA |  |
|  |  |  | I | GATGCCTAACAAGCTCAGGGGAG |  |
| Exp1R2A17 | S | Homo | I | GATGCCTAACAAGCTCAGGGGAG |  |
|  |  |  | I | GATGCCTAACAAGCTCAGGGGAG |  |
|  | T | Homo | I | GATGCCTAACAAGCGCAGGGGAA |  |
|  |  |  | I | GATGCCTAACAAGCGCAGGGGAA |  |
| Exp1R2A18 | S | Homo | I | GATGCCTAACAAGCACAGGGGAG |  |
|  |  |  | I | GATGCCTAACAAGCACAGGGGAG |  |
|  | T | Hetero | I | GATGCCTAACAAGCCCAGGGGAA |  |
|  |  |  | I | GATGCCTAACAAGCGCAGGGGAA |  |
| Exp1R2A19 | S | Homo | I | GATGCCTAACAAGCTCAGGGGAG |  |
|  |  |  | I | GATGCCTAACAAGCTCAGGGGAG |  |
|  | T | Homo | I | GATGCCTAACAAGCTCAGGGGAA |  |
|  |  |  | I | GATGCCTAACAAGCTCAGGGGAA |  |
| Exp1R2A20 | S | Hetero | I | GATGCCTAACAAGCACAGGGGAG |  |
|  |  |  | I | GATGCCTAACAAGCGCAGGGGAG |  |
|  | T | Hetero | I | GATGCCTAACAAGCACAGGGGAA |  |
|  |  |  | I | GATGCCTAACAAGCTCAGGGGAA |  |
|  | | | | | |
| Exp1R2G1 | S | Hetero | I | GATGCCTAACAAGCTCAGGGGAG |  |
|  |  |  | I | GATGCCTAACAAGC(+33 bps) |  |
|  | T | Homo | I | GATGCCTAACAAGCTCAGGGGAA |  |
|  |  |  | I | GATGCCTAACAAGCTCAGGGGAA |  |
| Exp1R2G2 | S | Homo | I | GATGCCTAACAAGCTCAGGGGAG |  |
|  |  |  | I | GATGCCTAACAAGCTCAGGGGAG |  |
|  | T |  | W | GATGCCTAACAAGC-CAGGGGAA |  |
|  |  |  | W | GATGCCTAACAAGC-CAGGGGAA |  |
| Exp1R2G3 | S |  | W | GATGCCTAACAAGC-CAGGGGAG |  |
|  |  |  | W | GATGCCTAACAAGC-CAGGGGAG |  |
|  | T |  | W | GATGCCTAACAAGC-CAGGGGAA |  |
|  |  |  | W | GATGCCTAACAAGC-CAGGGGAA |  |
| Exp1R2G4 | S |  | W | GATGCCTAACAAGC-CAGGGGAG |  |
|  |  |  | W | GATGCCTAACAAGC-CAGGGGAG |  |
|  | T |  | W | GATGCCTAACAAGC-CAGGGGAA |  |
|  |  |  | W | GATGCCTAACAAGC-CAGGGGAA |  |
| Exp1R2G5 | S | Homo | I | GATGCCTAACAAGCACAGGGGAG |  |
|  |  |  | I | GATGCCTAACAAGCACAGGGGAG |  |
|  | T |  | W | GATGCCTAACAAGC-CAGGGGAA |  |
|  |  |  | W | GATGCCTAACAAGC-CAGGGGAA |  |
| Exp1R2G6 | S |  | W | GATGCCTAACAAGC-CAGGGGAG |  |
|  |  |  | W | GATGCCTAACAAGC-CAGGGGAG |  |
|  | T |  | W | GATGCCTAACAAGC-CAGGGGAA |  |
|  |  |  | W | GATGCCTAACAAGC-CAGGGGAA |  |
| Exp1R2G7 | S | Hetero | W | GATGCCTAACAAGC-CAGGGGAG |  |
|  |  |  | I | GATGCCTAACAAGCTCAGGGGAG |  |
|  | T | Hetero | W | GATGCCTAACAAGC-CAGGGGAA |  |
|  |  |  | I | GATGCCTAACAAGCACAGGGGAA |  |
| Exp1R2G8 | S | Homo | I | GATGCCTAACAAGCTCAGGGGAG |  |
|  |  |  | I | GATGCCTAACAAGCTCAGGGGAG |  |
|  | T |  | W | GATGCCTAACAAGC-CAGGGGAA |  |
|  |  |  | W | GATGCCTAACAAGC-CAGGGGAA |  |
| Exp1R2G9 | S | Hetero | W | GATGCCTAACAAGC-CAGGGGAG |  |
|  |  |  | I | GATGCCTAACAAGCTCAGGGGAG |  |
|  | T | Hetero | W | GATGCCTAACAAGC-CAGGGGAA |  |
|  |  |  | I | GATGCCTAACAAGCTCAGGGGAA |  |
| Exp1R2G10 | S |  | W | GATGCCTAACAAGC-CAGGGGAG |  |
|  |  |  | W | GATGCCTAACAAGC-CAGGGGAG |  |
|  | T | Hetero | W | GATGCCTAACAAGC-CAGGGGAA |  |
|  |  | Hetero | I | GATGCCTAACAAGCTCAGGGGAA |  |
| Exp1R2G11 | S |  | W | GATGCCTAACAAGC-CAGGGGAG |  |
|  |  |  | W | GATGCCTAACAAGC-CAGGGGAG |  |
|  | T |  | W | GATGCCTAACAAGC-CAGGGGAA |  |
|  |  |  | W | GATGCCTAACAAGC-CAGGGGAA |  |
| Exp1R2G12 | S |  | W | GATGCCTAACAAGC-CAGGGGAG |  |
|  |  |  | W | GATGCCTAACAAGC-CAGGGGAG |  |
|  | T |  | W | GATGCCTAACAAGC-CAGGGGAA |  |
|  |  |  | W | GATGCCTAACAAGC-CAGGGGAA |  |
| Exp1R2G13 | S |  | W | GATGCCTAACAAGC-CAGGGGAG |  |
|  |  |  | W | GATGCCTAACAAGC-CAGGGGAG |  |
|  | T |  | W | GATGCCTAACAAGC-CAGGGGAA |  |
|  |  |  | W | GATGCCTAACAAGC-CAGGGGAA |  |
| Exp1R2G14 | S |  | W | GATGCCTAACAAGC-CAGGGGAG |  |
|  |  |  | W | GATGCCTAACAAGC-CAGGGGAG |  |
|  | T |  | W | GATGCCTAACAAGC-CAGGGGAA |  |
|  |  |  | W | GATGCCTAACAAGC-CAGGGGAA |  |
| Exp1R2G15 | S |  | W | GATGCCTAACAAGC-CAGGGGAG |  |
|  |  |  | W | GATGCCTAACAAGC-CAGGGGAG |  |
|  | T |  | W | GATGCCTAACAAGC-CAGGGGAA |  |
|  |  |  | W | GATGCCTAACAAGC-CAGGGGAA |  |
| Exp1R2G16 | S | Homo | I | GATGCCTAACAAGCAACAGGGGA |  |
|  |  |  | I | GATGCCTAACAAGCAACAGGGGA |  |
|  | T |  | W | GATGCCTAACAAGC-CAGGGGAA |  |
|  |  |  | W | GATGCCTAACAAGC-CAGGGGAA |  |
| Exp1R2G17 | S |  | W | GATGCCTAACAAGC-CAGGGGAG |  |
|  |  |  | W | GATGCCTAACAAGC-CAGGGGAG |  |
|  | T |  | W | GATGCCTAACAAGC-CAGGGGAA |  |
|  |  |  | W | GATGCCTAACAAGC-CAGGGGAA |  |
| Exp1R2G18 | S |  | W | GATGCCTAACAAGC-CAGGGGAG |  |
|  |  |  | W | GATGCCTAACAAGC-CAGGGGAG |  |
|  | T |  | W | GATGCCTAACAAGC-CAGGGGAA |  |
|  |  |  | W | GATGCCTAACAAGC-CAGGGGAA |  |
| Exp1R2G19 | S |  | W | GATGCCTAACAAGC-CAGGGGAG |  |
|  |  |  | W | GATGCCTAACAAGC-CAGGGGAG |  |
|  | T |  | W | GATGCCTAACAAGC-CAGGGGAA |  |
|  |  |  | W | GATGCCTAACAAGC-CAGGGGAA |  |
| Exp1R2G20 | S |  | W | GATGCCTAACAAGC-CAGGGGAG |  |
|  |  |  | W | GATGCCTAACAAGC-CAGGGGAG |  |
|  | T |  | W | GATGCCTAACAAGC-CAGGGGAA |  |
|  |  |  | W | GATGCCTAACAAGC-CAGGGGAA |  |

| **Sample no.** | **Genome type** | **genotype** | **Mutation type** | ***NtPDS* sequences** |  |
| --- | --- | --- | --- | --- | --- |
| Wild type | S |  | W | GATGCCTAACAAGC-CAGGGGAG |  |
|  | T |  | W | GATGCCTAACAAGC-CAGGGGAA |  |
|  | | | | |  |
| Exp1R3A1 | S | Homo | I | GATGCCTAACAAGCTCAGGGGAG |  |
|  |  |  | I | GATGCCTAACAAGCTCAGGGGAG |  |
|  | T | Hetero | I | GATGCCTAACAAGCCCAGGGGAA |  |
|  |  |  | I | GATGCCTAACAAGCTCAGGGGAA |  |
| Exp1R3A2 | S | Homo | I | GATGCCTAACAAGCTCAGGGGAG |  |
|  |  |  | I | GATGCCTAACAAGCTCAGGGGAG |  |
|  | T | Hetero | I | GATGCCTAACAAGCCCAGGGGAA |  |
|  |  |  | I | GATGCCTAACAAGCTCAGGGGAA |  |
| Exp1R3A3 | S | Hetero | I | GATGCCTAACAAGCACAGGGGAG |  |
|  |  |  | I | GATGCCTAACAAGAGCAGGGGAG |  |
|  | T | Hetero | I | GATGCCTAACAAGCCCAGGGGAA |  |
|  |  |  | I | GATGCCTAACAAGCTCAGGGGAA |  |
| Exp1R3A4 | S | Hetero | I | GATGCCTAACAAGCTCAGGGGAG |  |
|  |  |  | I | GATGCCTAACAAGCGCAGGGGAG |  |
|  | T | Homo | I | GATGCCTAACAAGCACAGGGGAA |  |
|  |  |  | I | GATGCCTAACAAGCACAGGGGAA |  |
| Exp1R3A5 | S | Hetero | I | GATGCCTAACAAGCTCAGGGGAG |  |
|  |  |  | I | GATGCCTAACAAGCACAGGGGAG |  |
|  | T | Homo | I | GATGCCTAACAAGCACAGGGGAA |  |
|  |  |  | I | GATGCCTAACAAGCACAGGGGAA |  |
| Exp1R3A6 | S | Hetero | I | GATGCCTAACAAGCTCAGGGGAG |  |
|  |  |  | I | GATGCCTAACAAGCCCAGGGGAG |  |
|  | T | Hetero | I | GATGCCTAACAAGCCCAGGGGAA |  |
|  |  |  | D | GATGCCTAACAAGC(-25 bps) |  |
| Exp1R3A7 | S | Hetero | I | GATGCCTAACAAGCACAGGGGAG |  |
|  |  |  | D | GATGCCTAACAAGC---GGGGAG |  |
|  | T | Homo | I | GATGCCTAACAAGCCCAGGGGAA |  |
|  |  |  | I | GATGCCTAACAAGCCCAGGGGAA |  |
| Exp1R3A8 | S | Hetero | I | GATGCCTAACAAGCTCAGGGGAG |  |
|  |  |  | I | GATGCCTAACAAGC(+10 bps) |  |
|  | T | Hetero | I | GATGCCTAACAAGCACAGGGGAA |  |
|  |  |  | I | GATGCCTAACAAGCTCAGGGGAA |  |
| Exp1R3A9 | S | Homo | I | GATGCCTAACAAGCTCAGGGGAG |  |
|  |  |  | I | GATGCCTAACAAGCTCAGGGGAG |  |
|  | T | Homo | I | GATGCCTAACAAGCGCAGGGGAA |  |
|  |  |  | I | GATGCCTAACAAGCGCAGGGGAA |  |
| Exp1R3A10 | S | Hetero | I | GATGCCTAACAAGCTCAGGGGAG |  |
|  |  |  | I | GATGCCTAACAAGC(+592 bps) |  |
|  | T | Homo | I | GATGCCTAACAAGCTCAGGGGAA |  |
|  |  |  | I | GATGCCTAACAAGCTCAGGGGAA |  |
| Exp1R3A11 | S | Homo | I | GATGCCTAACAAGCACAGGGGAG |  |
|  |  |  | I | GATGCCTAACAAGCACAGGGGAG |  |
|  | T | Homo | I | GATGCCTAACAAGCTCAGGGGAA |  |
|  |  |  | I | GATGCCTAACAAGCTCAGGGGAA |  |
| Exp1R3A12 | S | Hetero | I | GATGCCTAACAAGCGCAGGGGAG |  |
|  |  |  | I | GATGCCTAACAAGC(+73 bps) |  |
|  | T | Hetero | I | GATGCCTAACAAGCTCAGGGGAA |  |
|  |  |  | I | GATGCCTAACAAGCACAGGGGAA |  |
| Exp1R3A13 | S | Hetero | I | GATGCCTAACAAGCTCAGGGGAG |  |
|  |  |  | I | GATGCCTAACAAGCGCAGGGGAG |  |
|  | T | Hetero | I | GATGCCTAACAAGCTCAGGGGAA |  |
|  |  |  | I | GATGCCTAACAAGCGCAGGGGAA |  |
| Exp1R3A14 | S | Homo | I | GATGCCTAACAAGCACAGGGGAG |  |
|  |  |  | I | GATGCCTAACAAGCACAGGGGAG |  |
|  | T | Homo | I | GATGCCTAACAAGCACAGGGGAA |  |
|  |  |  | I | GATGCCTAACAAGCACAGGGGAA |  |
| Exp1R3A15 | S | Hetero | I | GATGCCTAACAAGCACAGGGGAG |  |
|  |  |  | I | GATGCCTAACAAGCCCAGGGGAG |  |
|  | T | Homo | I | GATGCCTAACAAGCTCAGGGGAA |  |
|  |  |  | I | GATGCCTAACAAGCTCAGGGGAA |  |
| Exp1R3A16 | S | Hetero | I | GATGCCTAACAAGCACAGGGGAG |  |
|  |  |  | I | GATGCCTAACAAGCTCAGGGGAG |  |
|  | T | Hetero | I | GATGCCTAACAAGCTCAGGGGAA |  |
|  |  |  | I | GATGCCTAACAAGCGCAGGGGAA |  |
| Exp1R3A17 | S | Homo | I | GATGCCTAACAAGCTCAGGGGAG |  |
|  |  |  | I | GATGCCTAACAAGCTCAGGGGAG |  |
|  | T | Homo | I | GATGCCTAACAAGCTCAGGGGAA |  |
|  |  |  | I | GATGCCTAACAAGCTCAGGGGAA |  |
| Exp1R3A18 | S | Homo | I | GATGCCTAACAAGCTCAGGGGAG |  |
|  |  |  | I | GATGCCTAACAAGCTCAGGGGAG |  |
|  | T | Homo | I | GATGCCTAACAAGCTCAGGGGAA |  |
|  |  |  | I | GATGCCTAACAAGCTCAGGGGAA |  |
| Exp1R3A19 | S | Hetero | I | GATGCCTAACAAGCCCAGGGGAG |  |
|  |  |  | D | GATGCCTAACAAG--------AG |  |
|  | T | Hetero | I | GATGCCTAACAAGCACAGGGGAA |  |
|  |  |  | D | GATGCCTAACAAGC--AGGGGAA |  |
| Exp1R3A20 | S | Homo | D | GATGCCTAACAAG--CAGGGGAG |  |
|  |  |  | D | GATGCCTAACAAG--CAGGGGAG |  |
|  | T | Homo | I | GATGCCTAACAAGCACAGGGGAA |  |
|  |  |  | I | GATGCCTAACAAGCACAGGGGAA |  |
|  | | | | | |
| Exp1R3G1 | S |  | W | GATGCCTAACAAGC-CAGGGGAG |  |
|  |  |  | W | GATGCCTAACAAGC-CAGGGGAG |  |
|  | T | Hetero | W | GATGCCTAACAAGC-CAGGGGAA |  |
|  |  |  | I | GATGCCTAACAAGCACAGGGGAA |  |
| Exp1R3G2 | S |  | W | GATGCCTAACAAGC-CAGGGGAG |  |
|  |  |  | W | GATGCCTAACAAGC-CAGGGGAG |  |
|  | T |  | W | GATGCCTAACAAGC-CAGGGGAA |  |
|  |  |  | W | GATGCCTAACAAGC-CAGGGGAA |  |
| Exp1R3G3 | S |  | W | GATGCCTAACAAGC-CAGGGGAG |  |
|  |  |  | W | GATGCCTAACAAGC-CAGGGGAG |  |
|  | T |  | W | GATGCCTAACAAGC-CAGGGGAA |  |
|  |  |  | W | GATGCCTAACAAGC-CAGGGGAA |  |
| Exp1R3G4 | S |  | W | GATGCCTAACAAGC-CAGGGGAG |  |
|  |  |  | W | GATGCCTAACAAGC-CAGGGGAG |  |
|  | T |  | W | GATGCCTAACAAGC-CAGGGGAA |  |
|  |  |  | W | GATGCCTAACAAGC-CAGGGGAA |  |
| Exp1R3G5 | S |  | W | GATGCCTAACAAGC-CAGGGGAG |  |
|  |  |  | W | GATGCCTAACAAGC-CAGGGGAG |  |
|  | T |  | W | GATGCCTAACAAGC-CAGGGGAA |  |
|  |  |  | W | GATGCCTAACAAGC-CAGGGGAA |  |
| Exp1R3G6 | S | Hetero | W | GATGCCTAACAAGC-CAGGGGAG |  |
|  |  |  | I | GATGCCTAACAAGCGCAGGGGAG |  |
|  | T |  | W | GATGCCTAACAAGC-CAGGGGAA |  |
|  |  |  | W | GATGCCTAACAAGC-CAGGGGAA |  |
| Exp1R3G7 | S |  | W | GATGCCTAACAAGC-CAGGGGAG |  |
|  |  |  | W | GATGCCTAACAAGC-CAGGGGAG |  |
|  | T |  | W | GATGCCTAACAAGC-CAGGGGAA |  |
|  |  |  | W | GATGCCTAACAAGC-CAGGGGAA |  |
| Exp1R3G8 | S |  | W | GATGCCTAACAAGC-CAGGGGAG |  |
|  |  |  | W | GATGCCTAACAAGC-CAGGGGAG |  |
|  | T |  | W | GATGCCTAACAAGC-CAGGGGAA |  |
|  |  |  | W | GATGCCTAACAAGC-CAGGGGAA |  |
| Exp1R3G9 | S |  | W | GATGCCTAACAAGC-CAGGGGAG |  |
|  |  |  | W | GATGCCTAACAAGC-CAGGGGAG |  |
|  | T |  | W | GATGCCTAACAAGC-CAGGGGAA |  |
|  |  |  | W | GATGCCTAACAAGC-CAGGGGAA |  |
| Exp1R3G10 | S | Hetero | I | GATGCCTAACAAGCTCAGGGGAG |  |
|  |  |  | I | GATGCCTAACAAGCGCAGGGGAG |  |
|  | T | Hetero | W | GATGCCTAACAAGC-CAGGGGAA |  |
|  |  |  | I | GATGCCTAACAAGCTCAGGGGAA |  |
| Exp1R3G11 | S |  | W | GATGCCTAACAAGC-CAGGGGAG |  |
|  |  |  | W | GATGCCTAACAAGC-CAGGGGAG |  |
|  | T |  | W | GATGCCTAACAAGC-CAGGGGAA |  |
|  |  |  | W | GATGCCTAACAAGC-CAGGGGAA |  |
| Exp1R3G12 | S |  | W | GATGCCTAACAAGC-CAGGGGAG |  |
|  |  |  | W | GATGCCTAACAAGC-CAGGGGAG |  |
|  | T |  | W | GATGCCTAACAAGC-CAGGGGAA |  |
|  |  |  | W | GATGCCTAACAAGC-CAGGGGAA |  |
| Exp1R3G13 | S | Hetero | W | GATGCCTAACAAGC-CAGGGGAG |  |
|  |  |  | D | GATGCCTAACAAGC---GGGGAG |  |
|  | T |  | W | GATGCCTAACAAGC-CAGGGGAA |  |
|  |  |  | W | GATGCCTAACAAGC-CAGGGGAA |  |
| Exp1R3G14 | S | Hetero | W | GATGCCTAACAAGC-CAGGGGAG |  |
|  |  |  | I | GATGCCTAACAAGCTCAGGGGAG |  |
|  | T |  | W | GATGCCTAACAAGC-CAGGGGAA |  |
|  |  |  | W | GATGCCTAACAAGC-CAGGGGAA |  |
| Exp1R3G15 | S |  | W | GATGCCTAACAAGC-CAGGGGAG |  |
|  |  |  | W | GATGCCTAACAAGC-CAGGGGAG |  |
|  | T |  | W | GATGCCTAACAAGC-CAGGGGAA |  |
|  |  |  | W | GATGCCTAACAAGC-CAGGGGAA |  |
| Exp1R3G16 | S |  | W | GATGCCTAACAAGC-CAGGGGAG |  |
|  |  |  | W | GATGCCTAACAAGC-CAGGGGAG |  |
|  | T |  | W | GATGCCTAACAAGC-CAGGGGAA |  |
|  |  |  | W | GATGCCTAACAAGC-CAGGGGAA |  |
| Exp1R3G17 | S |  | W | GATGCCTAACAAGC-CAGGGGAG |  |
|  |  |  | W | GATGCCTAACAAGC-CAGGGGAG |  |
|  | T |  | W | GATGCCTAACAAGC-CAGGGGAA |  |
|  |  |  | W | GATGCCTAACAAGC-CAGGGGAA |  |
| Exp1R3G18 | S | Hetero | I | GATGCCTAACAAGCTCAGGGGAG |  |
|  |  |  | I | GATGCCTAACAAGCGCAGGGGAG |  |
|  | T |  | W | GATGCCTAACAAGC-CAGGGGAA |  |
|  |  |  | W | GATGCCTAACAAGC-CAGGGGAA |  |
| Exp1R3G19 | S | Hetero | I | GATGCCTAACAAGCTCAGGGGAG |  |
|  |  |  | I | GATGCCTAACAAGCGCAGGGGAG |  |
|  | T |  | W | GATGCCTAACAAGC-CAGGGGAA |  |
|  |  |  | W | GATGCCTAACAAGC-CAGGGGAA |  |
| Exp1R3G20 | S |  | W | GATGCCTAACAAGC-CAGGGGAG |  |
|  |  |  | W | GATGCCTAACAAGC-CAGGGGAG |  |
|  | T |  | W | GATGCCTAACAAGC-CAGGGGAA |  |
|  |  |  | W | GATGCCTAACAAGC-CAGGGGAA |  |

| **Regenerate no.** | **Genome type** | **genotype** | **Mutation type** | ***NtPDS* sequences** |  |
| --- | --- | --- | --- | --- | --- |
| Wild type | S |  |  | GATGCCTAACAAGC-CAGGGGAG |  |
|  | T |  |  | GATGCCTAACAAGC-CAGGGGAA |  |
|  | | | | | |
| Exp2R1A1 | S | Homo | I | GATGCCTAACAAGCTCAGGGGAG |  |
|  |  |  | I | GATGCCTAACAAGCTCAGGGGAG |  |
|  | T | Homo | I | GATGCCTAACAAGCTCAGGGGAA |  |
|  |  |  | I | GATGCCTAACAAGCTCAGGGGAA |  |
| Exp2R1A2 | S | Hetero | I | GATGCCTAACAAGCACAGGGGAG |  |
|  |  |  | I | GATGCCTAACAAGCGCAGGGGAG |  |
|  | T | Hetero | I | GATGCCTAACAAGCTCAGGGGAA |  |
|  |  |  | I | GATGCCTAACAAGCACAGGGGAA |  |
| Exp2R1A3 | S | Homo | D | GATGCCT--------CAGGGGAG |  |
|  |  |  | D | GATGCCT--------CAGGGGAG |  |
|  | T | Hetero | D | GATGCCT--------CAGGGGAA |  |
|  |  |  | I | GATGCCTAACAAGC(+172 bps) |  |
| Exp2R1A4 | S | Homo | I | GATGCCTAACAAGCTCAGGGGAG |  |
|  |  |  | I | GATGCCTAACAAGCTCAGGGGAG |  |
|  | T | Homo | I | GATGCCTAACAAGCGCAGGGGAA |  |
|  |  |  | I | GATGCCTAACAAGCGCAGGGGAA |  |
| Exp2R1A5 | S | Homo | I | GATGCCTAACAAGCACAGGGGAG |  |
|  |  |  | D | GATGCCTAACAAGCACAGGGGAG |  |
|  | T | Homo | D | GATGCCTAACAAGC--AGGGGAA |  |
|  |  |  | D | GATGCCTAACAAGC--AGGGGAA |  |
| Exp2R1A6 | S | Homo | D | GATGCCTAAC------AGGGGAG |  |
|  |  |  | D | GATGCCTAAC------AGGGGAG |  |
|  |  | Homo | I | GATGCCTAACAAGCACAGGGGAA |  |
|  |  |  | I | GATGCCTAACAAGCACAGGGGAA |  |
| Exp2R1A7 | S | Hetero | I | GATGCCTAACAAGCTCAGGGGAG |  |
|  |  |  | I | GATGCCTAACAAGCCCAGGGGAG |  |
|  | T | Homo | I | GATGCCTAACAAGCTCAGGGGAA |  |
|  |  |  | I | GATGCCTAACAAGCTCAGGGGAA |  |
| Exp2R1A8 | S |  | D | GATGCCTAACAAGC--AGGGGAG |  |
|  |  |  | D | GATGCCTAACAAGC--AGGGGAG |  |
|  | T | Hetero | I | GATGCCTAACAAGCACAGGGGAA |  |
|  |  |  | I | GATGCCTAACAAGCGCAGGGGAA |  |
| Exp2R1A9 | S | Homo | I | GATGCCTAACAAGCTCAGGGGAG |  |
|  |  |  | I | GATGCCTAACAAGCTCAGGGGAG |  |
|  | T | Homo | I | GATGCCTAACAAGCTCAGGGGAA |  |
|  |  |  | I | GATGCCTAACAAGCTCAGGGGAA |  |
| Exp2R1A10 | S | Homo | I | GATGCCTAACAAGCTCAGGGGAG |  |
|  |  |  | I | GATGCCTAACAAGCTCAGGGGAG |  |
|  | T | Hetero | I | GATGCCTAACAAGCTCGGGGGAA |  |
|  |  |  | D | GATGCCTAACAAGC--AGGGGAA |  |
| Exp2R1A11 | S | Homo | I | GATGCCTAACAAGCACAGGGGAG |  |
|  |  |  | I | GATGCCTAACAAGCACAGGGGAG |  |
|  | T | Homo | I | GATGCCTAACAAGCTCAGGGGAA |  |
|  |  |  | I | GATGCCTAACAAGCTCAGGGGAA |  |
| Exp2R1A12 | S | Homo | I | GATGCCTAACAAGCACAGGGGAG |  |
|  |  |  | I | GATGCCTAACAAGCACAGGGGAG |  |
|  | T | Homo | I | GATGCCTAACAAGCTCAGGGGAA |  |
|  |  |  | I | GATGCCTAACAAGCTCAGGGGAA |  |
| Exp2R1A13 | S | Homo | I | GATGCCTAACAAGCGCAGGGGAG |  |
|  |  |  | I | GATGCCTAACAAGCGCAGGGGAG |  |
|  | T | Hetero | I | GATGCCTAACAAGCTCAGGGGAA |  |
|  |  |  | I | GATGCCTAACAAGCGCAGGGGAA |  |
| Exp2R1A14 | S | Homo | I | GATGCCTAACAAGCTCAGGGGAG |  |
|  |  |  | I | GATGCCTAACAAGCTCAGGGGAG |  |
|  | T | Homo | I | GATGCCTAACAAGCTCAGGGGAA |  |
|  |  |  | I | GATGCCTAACAAGCTCAGGGGAA |  |
| Exp2R1A15 | S | Homo | I | GATGCCTAACAAGCTCAGGGGAG |  |
|  |  |  | I | GATGCCTAACAAGCTCAGGGGAG |  |
|  | T | Homo | I | GATGCCTAACAAGCTCAGGGGAA |  |
|  |  |  | I | GATGCCTAACAAGCTCAGGGGAA |  |
| Exp2R1A16 | S | Hetero | I | GATGCCTAACAAGC(+477 bps) |  |
|  |  |  | I | GATGCCTAACAAGC(+475 bps) |  |
|  | T | Hetero | I | GATGCCTAACAAGCTCAGGGGAA |  |
|  |  |  | I | GATGCCTAACAAGCACAGGGGAA |  |
| Exp2R1A17 | S | Hetero | I | GATGCCTAACAAGCTTTGGTAGG |  |
|  |  |  | I | GATGCCTAACAAGC(+477 bps) |  |
|  | T | Homo | I | GATGCCTAACAAGCTCAGGGGAA |  |
|  |  |  | I | GATGCCTAACAAGCTCAGGGGAA |  |
| Exp2R1A18 | S | Homo | I | GATGCCTAACAAGCTCAGGGGAG |  |
|  |  |  | I | GATGCCTAACAAGCTCAGGGGAG |  |
|  | T | Homo | I | GATGCCTAACAAGCGCAGGGGAA |  |
|  |  |  | I | GATGCCTAACAAGCGCAGGGGAA |  |
| Exp2R1A19 | S | Homo | I | GATGCCTAACAAGCACAGGGGAG |  |
|  |  |  | I | GATGCCTAACAAGCACAGGGGAG |  |
|  | T | Homo | I | GATGCCTAACAAGCTCAGGGGAA |  |
|  |  |  | I | GATGCCTAACAAGCTCAGGGGAA |  |
| Exp2R1A20 | S | Homo | D | GATGCCTAACAAG--CAGGGGAG |  |
|  |  |  | D | GATGCCTAACAAG--CAGGGGAG |  |
|  | T | Hetero | I | GATGCCTAACAAGCTCAGGGGAA |  |
|  |  |  | I | GATGCCTAACAAGCGCAGGGGAA |  |
|  | | | | | |
| Exp2R1G1 | S | Homo | W | GATGCCTAACAAGC-CAGGGGAG |  |
|  |  |  | W | GATGCCTAACAAGC-CAGGGGAG |  |
|  | T | Homo | W | GATGCCTAACAAGC-CAGGGGAA |  |
|  |  |  | W | GATGCCTAACAAGC-CAGGGGAA |  |
| Exp2R1G2 | S | Homo | W | GATGCCTAACAAGC-CAGGGGAG |  |
|  |  |  | W | GATGCCTAACAAGC-CAGGGGAG |  |
|  | T | Homo | W | GATGCCTAACAAGC-CAGGGGAA |  |
|  |  |  | W | GATGCCTAACAAGC-CAGGGGAA |  |
| Exp2R1G3 | S | Homo | W | GATGCCTAACAAGC-CAGGGGAG |  |
|  |  |  | W | GATGCCTAACAAGC-CAGGGGAG |  |
|  | T | Homo | W | GATGCCTAACAAGC-CAGGGGAA |  |
|  |  |  | W | GATGCCTAACAAGC-CAGGGGAA |  |
| Exp2R1G4 | S | Homo | W | GATGCCTAACAAGC-CAGGGGAG |  |
|  |  |  | W | GATGCCTAACAAGC-CAGGGGAG |  |
|  | T | Homo | W | GATGCCTAACAAGC-CAGGGGAA |  |
|  |  |  | W | GATGCCTAACAAGC-CAGGGGAA |  |
| Exp2R1G5 | S | Homo | W | GATGCCTAACAAGC-CAGGGGAG |  |
|  |  |  | W | GATGCCTAACAAGC-CAGGGGAG |  |
|  | T | Homo | W | GATGCCTAACAAGC-CAGGGGAA |  |
|  |  |  | W | GATGCCTAACAAGC-CAGGGGAA |  |
| Exp2R1G6 | S | Hetero | I | GATGCCTAACAAGCTCAGGGGAG |  |
|  |  |  | W | GATGCCTAACAAGC-CAGGGGAG |  |
|  | T | Hetero | W | GATGCCTAACAAGC-CAGGGGAA |  |
|  |  |  | I | GATGCCTAACAAGCTCAGGGGAA |  |
| Exp2R1G7 | S | Homo | W | GATGCCTAACAAGC-CAGGGGAG |  |
|  |  |  | W | GATGCCTAACAAGC-CAGGGGAG |  |
|  | T | Homo | W | GATGCCTAACAAGC-CAGGGGAA |  |
|  |  |  | W | GATGCCTAACAAGC-CAGGGGAA |  |
| Exp2R1G8 | S | Homo | W | GATGCCTAACAAGC-CAGGGGAG |  |
|  |  |  | W | GATGCCTAACAAGC-CAGGGGAG |  |
|  | T | Homo | W | GATGCCTAACAAGC-CAGGGGAA |  |
|  |  |  | W | GATGCCTAACAAGC-CAGGGGAA |  |
| Exp2R1G9 | S | Hetero | W | GATGCCTAACAAGC-CAGGGGAG |  |
|  |  |  | I | GATGCCTAACAAGCACAGGGGAG |  |
|  | T | Homo | W | GATGCCTAACAAGC-CAGGGGAA |  |
|  |  |  | W | GATGCCTAACAAGC-CAGGGGAA |  |
| Exp2R1G10 | S | Homo | W | GATGCCTAACAAGC-CAGGGGAG |  |
|  |  |  | W | GATGCCTAACAAGC-CAGGGGAG |  |
|  | T | Homo | W | GATGCCTAACAAGC-CAGGGGAA |  |
|  |  |  | W | GATGCCTAACAAGC-CAGGGGAA |  |
| Exp2R1G11 | S | Hetero | R | GATGCCTAACAAGC-TCAGGGAG |  |
|  |  |  | D | GATGCCTAACAAGC-(-17 bps) |  |
|  | T | Hetero | I | GATGCCTAACAAGCACAGGGGAA |  |
|  |  |  | W | GATGCCTAACAAGC-CAGGGGAA |  |
| Exp2R1G12 | S | Homo | W | GATGCCTAACAAGC-CAGGGGAG |  |
|  |  |  | W | GATGCCTAACAAGC-CAGGGGAG |  |
|  | T | Homo | W | GATGCCTAACAAGC-CAGGGGAA |  |
|  |  |  | W | GATGCCTAACAAGC-CAGGGGAA |  |
| Exp2R1G13 | S | Homo | W | GATGCCTAACAAGC-CAGGGGAG |  |
|  |  |  | W | GATGCCTAACAAGC-CAGGGGAG |  |
|  | T | Homo | W | GATGCCTAACAAGC-CAGGGGAA |  |
|  |  |  | W | GATGCCTAACAAGC-CAGGGGAA |  |
| Exp2R1G14 | S | Hetero | I | GATGCCTAACAAGCGCAGGGGAG |  |
|  |  |  | W | GATGCCTAACAAGC-CAGGGGAG |  |
|  | T | Homo | W | GATGCCTAACAAGC-CAGGGGAA |  |
|  |  |  | W | GATGCCTAACAAGC-CAGGGGAA |  |
| Exp2R1G15 | S | Hetero | I | GATGCCTAACAAGCACAGGGGAG |  |
|  |  |  | W | GATGCCTAACAAGC-CAGGGGAG |  |
|  | T | Homo | W | GATGCCTAACAAGC-CAGGGGAA |  |
|  |  |  | W | GATGCCTAACAAGC-CAGGGGAA |  |
| Exp2R1G16 | S | Homo | W | GATGCCTAACAAGC-CAGGGGAG |  |
|  |  |  | W | GATGCCTAACAAGC-CAGGGGAG |  |
|  | T | Homo | W | GATGCCTAACAAGC-CAGGGGAA |  |
|  |  |  | W | GATGCCTAACAAGC-CAGGGGAA |  |
| Exp2R1G17 | S | Homo | W | GATGCCTAACAAGC-CAGGGGAG |  |
|  |  |  | W | GATGCCTAACAAGC-CAGGGGAG |  |
|  | T | Homo | W | GATGCCTAACAAGC-CAGGGGAA |  |
|  |  |  | W | GATGCCTAACAAGC-CAGGGGAA |  |
| Exp2R1G18 | S | Homo | I | GATGCCTAACAAGCTCAGGGGAG |  |
|  |  |  | I | GATGCCTAACAAGCTCAGGGGAG |  |
|  | T | Hetero | D | GATGCCTAA-------AGGGGAA |  |
|  |  |  | W | GATGCCTAACAAGC-CAGGGGAA |  |
| Exp2R1G19 | S | Hetero | I | GATGCCTAACAAGCACAGGGGAG |  |
|  |  |  | W | GATGCCTAACAAGC-CAGGGGAG |  |
|  | T | Homo | W | GATGCCTAACAAGC-CAGGGGAA |  |
|  |  |  | W | GATGCCTAACAAGC-CAGGGGAA |  |
| Exp2R1G20 | S | Homo | W | GATGCCTAACAAGC-CAGGGGAG |  |
|  |  |  | W | GATGCCTAACAAGC-CAGGGGAG |  |
|  | T | Homo | W | GATGCCTAACAAGC-CAGGGGAA |  |
|  |  |  | W | GATGCCTAACAAGC-CAGGGGAA |  |

| **Sample no.** | **Genome type** | **genotype** | **Mutation type** | ***NtPDS* sequences** |  |
| --- | --- | --- | --- | --- | --- |
| Wild type |  |  | W | GATGCCTAACAAGC-CAGGGGAG |  |
|  |  |  | W | GATGCCTAACAAGC-CAGGGGAA |  |
|  | | | | | |
| Exp2R2A1 | S | Homo | I | GATGCCTAACAAGCGCAGGGGAA |  |
|  |  |  | I | GATGCCTAACAAGCGCAGGGGAA |  |
|  | T | Homo | I | GATGCCTAACAAGCGCAGGGGAA |  |
|  |  |  | I | GATGCCTAACAAGCGCAGGGGAA |  |
| Exp2R2A2 | S | Homo | I | GATGCCTAACAAGCTCAGGGGAG |  |
|  |  |  | I | GATGCCTAACAAGCTCAGGGGAG |  |
|  | T | Homo | I | GATGCCTAACAAGCTCAGGGGAA |  |
|  |  |  | I | GATGCCTAACAAGCTCAGGGGAA |  |
| Exp2R2A3 | S | Homo | I | GATGCCTAACAAGCTCAGGGGAG |  |
|  |  |  | I | GATGCCTAACAAGCTCAGGGGAG |  |
|  | T | Hetero | I | GATGCCTAACAAGCTCAGGGGAA |  |
|  |  |  | I | GATGCCTAACAAGCGCAGGGGAA |  |
| Exp2R2A4 | S | Homo | I | GATGCCTAACAAGCTCAGGGGAG |  |
|  |  |  | I | GATGCCTAACAAGCTCAGGGGAG |  |
|  | T | Hetero | I | GATGCCTAACAAGCTCAGGGGAA |  |
|  |  |  | I | GATGCCTAACAAGCACAGGGGAA |  |
| Exp2R2A5 | S | Hetero | I | GATGCCTAACAAGCGCAGGGGAG |  |
|  |  |  | I | GATGCCTAACAAGCACAGGGGAG |  |
|  | T | Homo | I | GATGCCTAACAAGCTCAGGGGAA |  |
|  |  |  | I | GATGCCTAACAAGCTCAGGGGAA |  |
| Exp2R2A6 | S | Hetero | I | GATGCCTAACAAGCTCAGGGGAG |  |
|  |  |  | I | GATGCCTAACAAGCACAGGGGAG |  |
|  | T | Homo | I | GATGCCTAACAAGCTCAGGGGAA |  |
|  |  |  | I | GATGCCTAACAAGCTCAGGGGAA |  |
| Exp2R2A7 | S | Homo | I | GATGCCTAACAAGCGCAGGGGAG |  |
|  |  |  | I | GATGCCTAACAAGCGCAGGGGAG |  |
|  | T | Hetero | I | GATGCCTAACAAGCTCAGGGGAA |  |
|  |  |  | I | GATGCCTAACAAGCACAGGGGAA |  |
| Exp2R2A8 | S | Homo | I | GATGCCTAACAAGCACAGGGGAG |  |
|  |  |  | I | GATGCCTAACAAGCACAGGGGAG |  |
|  | T | Homo | I | GATGCCTAACAAGCTCAGGGGAA |  |
|  |  |  | I | GATGCCTAACAAGCTCAGGGGAA |  |
| Exp2R2A9 | S | Hetero | I | GATGCCTAACAAGCACAGGGGAG |  |
|  |  |  | I | GATGCCTAACAAGCTCAGGGGAG |  |
|  | T | Hetero | I | GATGCCTAACAAGCTCAGGGGAA |  |
|  |  |  | I | GATGCCTAACAAGCACAGGGGAA |  |
| Exp2R2A10 | S | Homo | I | GATGCCTAACAAGCACAGGGGAG |  |
|  |  |  | I | GATGCCTAACAAGCACAGGGGAG |  |
|  | T | Homo | I | GATGCCTAACAAGCTCAGGGGAA |  |
|  |  |  | I | GATGCCTAACAAGCTCAGGGGAA |  |
| Exp2R2A11 | S | Homo | D | GATGCCTAACAAGCA--GGGGAG |  |
|  |  |  | D | GATGCCTAACAAGCA--GGGGAG |  |
|  | T | Hetero | I | GATGCCTAACAAGCTCAGGGGAA |  |
|  |  |  | I | GATGCCTAACAAGCACAGGGGAA |  |
| Exp2R2A12 | S | Homo | I | GATGCCTAACAAGCACAGGGGAG |  |
|  |  |  | I | GATGCCTAACAAGCACAGGGGAG |  |
|  | T | Homo | I | GATGCCTAACAAGCACAGGGGAA |  |
|  |  |  | I | GATGCCTAACAAGCACAGGGGAA |  |
| Exp2R2A13 | S | Homo | I | GATGCCTAACAAGCACAGGGGAG |  |
|  |  |  | I | GATGCCTAACAAGCACAGGGGAG |  |
|  | T | Homo | I | GATGCCTAACAAGCCCAGGGGAA |  |
|  |  |  | I | GATGCCTAACAAGCCCAGGGGAA |  |
| Exp2R2A14 | S | Homo | I | GATGCCTAACAAGCTCAGGGGAG |  |
|  |  |  | I | GATGCCTAACAAGCTCAGGGGAG |  |
|  | T | Homo | I | GATGCCTAACAAGCACAGGGGAA |  |
|  |  |  | I | GATGCCTAACAAGCACAGGGGAA |  |
| Exp2R2A15 | S | Hetero | I | GATGCCTAACAAGCTCAGGGGAG |  |
|  |  |  | I | GATGCCTAACAAGCACAGGGGAG |  |
|  | T | Hetero | I | GATGCCTAACAAGCTCAGGGGAA |  |
|  |  |  | D | GATGCCTAACAAG--CAGGGGAA |  |
| Exp2R2A16 | S | Hetero | I | GATGCCTAACAAGCTCAGGGGAG |  |
|  |  |  | I | GATGCCTAACAAGCACAGGGGAG |  |
|  | T | Hetero | I | GATGCCTAACAAGCTCAGGGGAA |  |
|  |  |  | I | GATGCCTAACAAGCACAGGGGAA |  |
| Exp2R2A17 | S | Hetero | I | GATGCCTAACAAGCTCAGGGGAG |  |
|  |  |  | I | GATGCCTAACAAGCACAGGGGAG |  |
|  | T | Hetero | I | GATGCCTAACAAGCACAGGGGAA |  |
|  |  |  | I | GATGCCTAACAAGCGCAGGGGAA |  |
| Exp2R2A18 | S | Hetero | I | GATGCCTAACAAGCTCAGGGGAG |  |
|  |  |  | I | GATGCCTAACAAGCACAGGGGAG |  |
|  | T | Hetero | I | GATGCCTAACAAGCACAGGGGAA |  |
|  |  |  | I | GATGCCTAACAAGCGCAGGGGAA |  |
| Exp2R2A19 | S | Homo | I | GATGCCTAACAAGCTCAGGGGAG |  |
|  |  |  | I | GATGCCTAACAAGCTCAGGGGAG |  |
|  | T | Hetero | I | GATGCCTAACAAGCTCAGGGGAA |  |
|  |  |  | D | GATGCCTAACAAGC--AGGGGAA |  |
| Exp2R2A20 | S | Hetero | I | GATGCCTAACAAGCACAGGGGAG |  |
|  |  |  | D | GATGCCTAACAAGC-----GGAG |  |
|  | T | Homo | I | GATGCCTAACAAGCTCAGGGGAA |  |
|  |  |  | I | GATGCCTAACAAGCTCAGGGGAA |  |
|  | | | | | |
| Exp2R2G1 | S | Hetero | I | GATGCCTAACAAGCACAGGGGAG |  |
|  |  |  | I | GATGCCTAACAAGCTCAGGGGAG |  |
|  | T | Hetero | W | GATGCCTAACAAGC-CAGGGGAA |  |
|  |  |  | I | GATGCCTAACAAGCCCAGGGGAA |  |
| Exp2R2G2 | S | Hetero | W | GATGCCTAACAAGC-CAGGGGAG |  |
|  |  |  | I | GATGCCTAACAAGCACAGGGGAG |  |
|  | T | Homo | I | GATGCCTAACAAGCGCAGGGGAA |  |
|  |  |  | I | GATGCCTAACAAGCGCAGGGGAA |  |
| Exp2R2G3 | S | Hetero | I | GATGCCTAACAAGCTCAGGGGAG |  |
|  |  |  | I | GATGCCTAACAAGCGCAGGGGAG |  |
|  | T |  | W | GATGCCTAACAAGC-CAGGGGAA |  |
|  |  |  | W | GATGCCTAACAAGC-CAGGGGAA |  |
| Exp2R2G4 | S | Hetero | I | GATGCCTAACAAGCTCAGGGGAG |  |
|  |  |  | I | GATGCCTAACAAGCACAGGGGAG |  |
|  | T |  | W | GATGCCTAACAAGC-CAGGGGAA |  |
|  |  |  | W | GATGCCTAACAAGC-CAGGGGAA |  |
| Exp2R2G5 | S | Hetero | W | GATGCCTAACAAGC-CAGGGGAG |  |
|  |  |  | D | GATGCCTAACA----CAGGGGAG |  |
|  | T | Hetero | W | GATGCCTAACAAGC-CAGGGGAA |  |
|  |  |  | I | GATGCCTAACAAGCGCAGGGGAA |  |
| Exp2R2G6 | S |  | W | GATGCCTAACAAGC-CAGGGGAG |  |
|  |  |  | W | GATGCCTAACAAGC-CAGGGGAG |  |
|  | T |  | W | GATGCCTAACAAGC-CAGGGGAA |  |
|  |  |  | W | GATGCCTAACAAGC-CAGGGGAA |  |
| Exp2R2G7 | S |  | W | GATGCCTAACAAGC-CAGGGGAG |  |
|  |  |  | W | GATGCCTAACAAGC-CAGGGGAG |  |
|  | T |  | W | GATGCCTAACAAGC-CAGGGGAA |  |
|  |  |  | W | GATGCCTAACAAGC-CAGGGGAA |  |
| Exp2R2G8 | S |  | W | GATGCCTAACAAGC-CAGGGGAG |  |
|  |  |  | W | GATGCCTAACAAGC-CAGGGGAG |  |
|  | T |  | W | GATGCCTAACAAGC-CAGGGGAA |  |
|  |  |  | W | GATGCCTAACAAGC-CAGGGGAA |  |
| Exp2R2G9 | S |  | W | GATGCCTAACAAGC-CAGGGGAG |  |
|  |  |  | W | GATGCCTAACAAGC-CAGGGGAG |  |
|  | T |  | W | GATGCCTAACAAGC-CAGGGGAA |  |
|  |  |  | W | GATGCCTAACAAGC-CAGGGGAA |  |
| Exp2R2G10 | S |  | W | GATGCCTAACAAGC-CAGGGGAG |  |
|  |  |  | W | GATGCCTAACAAGC-CAGGGGAG |  |
|  | T |  | W | GATGCCTAACAAGC-CAGGGGAA |  |
|  |  |  | W | GATGCCTAACAAGC-CAGGGGAA |  |
| Exp2R2G11 | S |  | W | GATGCCTAACAAGC-CAGGGGAG |  |
|  |  |  | W | GATGCCTAACAAGC-CAGGGGAG |  |
|  | T |  | W | GATGCCTAACAAGC-CAGGGGAA |  |
|  |  |  | W | GATGCCTAACAAGC-CAGGGGAA |  |
| Exp2R2G12 | S | Hetero | I | GATGCCTAACAAGCTCAGGGGAG |  |
|  |  |  | I | GATGCCTAACAAGCGCAGGGGAG |  |
|  | T | Hetero | W | GATGCCTAACAAGC-CAGGGGAA |  |
|  |  |  | I | GATGCCTAACAAGCTCAGGGGAA |  |
| Exp2R2G13 | S | Hetero | W | GATGCCTAACAAGC-CAGGGGAG |  |
|  |  |  | I | GATGCCTAACAAGCTCAGGGGAG |  |
|  | T |  | W | GATGCCTAACAAGC-CAGGGGAA |  |
|  |  |  | W | GATGCCTAACAAGC-CAGGGGAA |  |
| Exp2R2G14 | S | Hetero | I | GATGCCTAACAAGCTCAGGGGAG |  |
|  |  |  | I | GATGCCTAACAAGCGCAGGGGAG |  |
|  | T |  | W | GATGCCTAACAAGC-CAGGGGAA |  |
|  |  |  | W | GATGCCTAACAAGC-CAGGGGAA |  |
| Exp2R2G15 | S | Homo | I | GATGCCTAACAAGCTCAGGGGAG |  |
|  |  |  | I | GATGCCTAACAAGCTCAGGGGAG |  |
|  | T | Hetero | W | GATGCCTAACAAGC-CAGGGGAA |  |
|  |  |  | I | GATGCCTAACAAGCTCAGGGGAA |  |
| Exp2R2G16 | S | Hetero | D | GATGCCTAACAAGC-C-GGGGAG |  |
|  |  |  | I | GATGCCTAACAAGCGCAGGGGAG |  |
|  | T | Hetero | W | GATGCCTAACAAGC-CAGGGGAA |  |
|  |  |  | I | GATGCCTAACAAGCACAGGGGAA |  |
| Exp2R2G17 | S | Hetero | W | GATGCCTAACAAGC-CAGGGGAG |  |
|  |  |  | I | GATGCCTAACAAGCTCAGGGGAG |  |
|  | T |  | W | GATGCCTAACAAGC-CAGGGGAA |  |
|  |  |  | W | GATGCCTAACAAGC-CAGGGGAA |  |
| Exp2R2G18 | S |  | W | GATGCCTAACAAGC-CAGGGGAG |  |
|  |  |  | W | GATGCCTAACAAGC-CAGGGGAG |  |
|  | T |  | W | GATGCCTAACAAGC-CAGGGGAA |  |
|  |  |  | W | GATGCCTAACAAGC-CAGGGGAA |  |
| Exp2R2G19 | S |  | W | GATGCCTAACAAGC-CAGGGGAG |  |
|  |  |  | W | GATGCCTAACAAGC-CAGGGGAG |  |
|  | T |  | W | GATGCCTAACAAGC-CAGGGGAA |  |
|  |  |  | W | GATGCCTAACAAGC-CAGGGGAA |  |
| Exp2R2G20 | S |  | W | GATGCCTAACAAGC-CAGGGGAG |  |
|  |  |  | W | GATGCCTAACAAGC-CAGGGGAG |  |
|  | T |  | W | GATGCCTAACAAGC-CAGGGGAA |  |
|  |  |  | W | GATGCCTAACAAGC-CAGGGGAA |  |

| **Sample no.** | **Genome type** | **genotype** | **Mutation type** | ***NtPDS* sequences** |  |
| --- | --- | --- | --- | --- | --- |
| Wild type | S |  | W | GATGCCTAACAAGC-CAGGGGAG |  |
|  | T |  | W | GATGCCTAACAAGC-CAGGGGAA |  |
|  | | | | | |
| Exp2R3A1 | S | Hetero | I | GATGCCTAACAAGCTCAGGGGAG |  |
|  |  |  | I | GATGCCTAACAAGCCCAGGGGAG |  |
|  | T | Hetero | I | GATGCCTAACAAGCCCAGGGGAA |  |
|  |  |  | I | GATGCCTAACAAGCTCAGGGGAA |  |
| Exp2R3A2 | S | Homo | D | G--------------(-31 bps) |  |
|  |  |  | D | G--------------(-31 bps) |  |
|  | T | Hetero | I | GATGCCTAACAAGCTCAGGGGAA |  |
|  |  |  | D | GATGCCTAACAAGC--AGGGGAA |  |
| Exp2R3A3 | S | Homo | I | GATGCCTAACAAGCACAGGGGAG |  |
|  |  |  | I | GATGCCTAACAAGCACAGGGGAG |  |
|  | T | Hetero | I | GATGCCTAACAAGCTCAGGGGAA |  |
|  |  |  | I | GATGCCTAACAAGCACAGGGGAA |  |
| Exp2R3A4 | S | Hetero | I | GATGCCTAACAAGCACAGGGGAG |  |
|  |  |  | I | GATGCCTAACAAGCGCAGGGGAG |  |
|  | T | Homo | I | GATGCCTAACAAGCACAGGGGAA |  |
|  |  |  | I | GATGCCTAACAAGCACAGGGGAA |  |
| Exp2R3A5 | S | Hetero | I | GATGCCTAACAAGCTCAGGGGAG |  |
|  |  |  | D | GATGCCTAACAAGC--AGGGGAG |  |
|  | T | Hetero | I | GATGCCTAACAAGCTCAGGGGAA |  |
|  |  |  | I | GATGCCTAACAAGCACAGGGGAA |  |
| Exp2R3A6 | S | Homo | I | GATGCCTAACAAGCACAGGGGAG |  |
|  |  |  | I | GATGCCTAACAAGCACAGGGGAG |  |
|  | T | Homo | D | GATATTTGCGATGC--AGGGGAA |  |
|  |  |  | D | GATGCCTAACAAGC--AGGGGAA |  |
| Exp2R3A7 | S | Homo | I | GATGCCTAACAAGCTCAGGGGAG |  |
|  |  |  | I | GATGCCTAACAAGCTCAGGGGAG |  |
|  | T | Homo | I | GATGCCTAACAAGCACAGGGGAA |  |
|  |  |  | I | GATGCCTAACAAGCACAGGGGAA |  |
| Exp2R3A8 | S | Hetero | I | GATGCCTAACAAGCTCAGGGGAG |  |
|  |  |  | D | GATGCCTAACAAGC--AGGGGAG |  |
|  | T | Homo | I | GATGCCTAACAAGCTCAGGGGAA |  |
|  |  |  | I | GATGCCTAACAAGCTCAGGGGAA |  |
| Exp2R3A9 | S | Homo | I | GATGCCTAACAAGCTCAGGGGAG |  |
|  |  |  | I | GATGCCTAACAAGCTCAGGGGAG |  |
|  | T | Homo | I | GATGCCTAACAAGCACAGGGGAA |  |
|  |  |  | I | GATGCCTAACAAGCACAGGGGAA |  |
| Exp2R3A10 | S | Homo | I | GATGCCTAACAAGCACAGGGGAG |  |
|  |  |  | I | GATGCCTAACAAGCACAGGGGAG |  |
|  | T | Homo | I | GATGCCTAACAAGCACAGGGGAA |  |
|  |  |  | I | GATGCCTAACAAGCACAGGGGAA |  |
| Exp2R3A11 | S | Hetero | I | GATGCCTAACAAGC(+641 bps) |  |
|  |  |  | I | GATGCCTAACAAGCTCAGGGGAG |  |
|  | T | Homo | D | GATGCCTAACAAGC--AGGGGAA |  |
|  |  |  | D | GATGCCTAACAAGC--AGGGGAA |  |
| Exp2R3A12 | S | Homo | I | GATGCCTAACAAGCTCAGGGGAG |  |
|  |  |  | I | GATGCCTAACAAGCTCAGGGGAG |  |
|  | T | Homo | I | GATGCCTAACAAGCTCAGGGGAA |  |
|  |  |  | I | GATGCCTAACAAGCTCAGGGGAA |  |
| Exp2R3A13 | S | Hetero | I | GATGCCTAACAAGCTCAGGGGAG |  |
|  |  |  | I | GATGCCTAACAAGCACAGGGGAG |  |
|  | T | Hetero | D | GATGCCTAACAAGC--AGGGGAA |  |
|  |  |  | I | GATGCCTAACAAGCTCAGGGGAA |  |
| Exp2R3A14 | S | Homo | I | GATGCCTAACAAGCTCAGGGGAG |  |
|  |  |  | I | GATGCCTAACAAGCTCAGGGGAG |  |
|  | T | Homo | I | GATGCCTAACAAGCTCAGGGGAA |  |
|  |  |  | I | GATGCCTAACAAGCTCAGGGGAA |  |
| Exp2R3A15 | S | Hetero | I | GATGCCTAACAAGCTCAGGGGAG |  |
|  |  |  | I | GATGCCTAACAAGCACAGGGGAG |  |
|  | T | Homo | D | GATGCCTAACAAGC--AGGGGAA |  |
|  |  |  | D | GATGCCTAACAAGC--AGGGGAA |  |
| Exp2R3A16 | S | Homo | I | GATGCCTAACAAGCTCAGGGGAG |  |
|  |  |  | I | GATGCCTAACAAGCTCAGGGGAG |  |
|  | T | Hetero | I | GATGCCTAACAAGCTCAGGGGAG |  |
|  |  |  | I | GATGCCTAACAAGCGCAGGGGAG |  |
| Exp2R3A17 | S | Hetero | I | GATGCCTAACAAGCTCAGGGGAG |  |
|  |  |  | I | GATGCCTAACAAGCACAGGGGAG |  |
|  | T | Homo | I | GATGCCTAACAAGCTCAGGGGAA |  |
|  |  |  | I | GATGCCTAACAAGCTCAGGGGAA |  |
| Exp2R3A18 | S | Hetero | I | GATGCCTAACAAGCTCAGGGGAG |  |
|  |  |  | I | GATGCCTAACAAGCACAGGGGAG |  |
|  | T | Hetero | I | GATGCCTAACAAGCTCAGGGGAA |  |
|  |  |  | I | GATGCCTAACAAGCACAGGGGAA |  |
| Exp2R3A19 | S | Homo | I | GATGCCTAACAAGCTCAGGGGAG |  |
|  |  |  | I | GATGCCTAACAAGCTCAGGGGAG |  |
|  | T | Hetero | I | GATGCCTAACAAGCCCAGGGGAA |  |
|  |  |  | I | GATGCCTAACAAGCTCAGGGGAA |  |
| Exp2R3A20 | S | Hetero | I | GATGCCTAACAAGCTCAGGGGAG |  |
|  |  |  | I | GATGCCTAACAAGCACAGGGGAG |  |
|  | T | Hetero | I | GATGCCTAACAAGCACAGGGGAA |  |
|  |  |  | I | GATGCCTAACAAGCTCAGGGGAA |  |
|  | | | | | |
| Exp2R3G1 | S |  | W | GATGCCTAACAAGC-CAGGGGAG |  |
|  |  |  | W | GATGCCTAACAAGC-CAGGGGAG |  |
|  | T |  | W | GATGCCTAACAAGC-CAGGGGAA |  |
|  |  |  | W | GATGCCTAACAAGC-CAGGGGAA |  |
| Exp2R3G2 | S |  | W | GATGCCTAACAAGC-CAGGGGAG |  |
|  |  |  | W | GATGCCTAACAAGC-CAGGGGAG |  |
|  | T |  | W | GATGCCTAACAAGC-CAGGGGAA |  |
|  |  |  | W | GATGCCTAACAAGC-CAGGGGAA |  |
| Exp2R3G3 | S | Hetero | I | GATGCCTAACAAGCCCAGGGGAG |  |
|  |  |  | I | GATGCCTAACAAGCGCAGGGGAG |  |
|  | T |  | W | GATGCCTAACAAGC-CAGGGGAA |  |
|  |  |  | W | GATGCCTAACAAGC-CAGGGGAA |  |
| Exp2R3G4 | S |  | W | GATGCCTAACAAGC-CAGGGGAG |  |
|  |  |  | W | GATGCCTAACAAGC-CAGGGGAG |  |
|  | T |  | W | GATGCCTAACAAGC-CAGGGGAA |  |
|  |  |  | W | GATGCCTAACAAGC-CAGGGGAA |  |
| Exp2R3G5 | S | Hetero | I | GATGCCTAACAAGCTCAGGGGAG |  |
|  |  |  | I | GATGCCTAACAAGCGCAGGGGAG |  |
|  | T |  | W | GATGCCTAACAAGC-CAGGGGAA |  |
|  |  |  | W | GATGCCTAACAAGC-CAGGGGAA |  |
| Exp2R3G6 | S |  | W | GATGCCTAACAAGC-CAGGGGAG |  |
|  |  |  | W | GATGCCTAACAAGC-CAGGGGAG |  |
|  | T |  | W | GATGCCTAACAAGC-CAGGGGAA |  |
|  |  |  | W | GATGCCTAACAAGC-CAGGGGAA |  |
| Exp2R3G7 | S | Hetero | W | GATGCCTAACAAGC-CAGGGGAG |  |
|  |  |  | I | GATGCCTAACAAGCTCAGGGGAG |  |
|  | T |  | W | GATGCCTAACAAGC-CAGGGGAA |  |
|  |  |  | W | GATGCCTAACAAGC-CAGGGGAA |  |
| Exp2R3G8 | S |  | W | GATGCCTAACAAGC-CAGGGGAG |  |
|  |  |  | W | GATGCCTAACAAGC-CAGGGGAG |  |
|  | T |  | W | GATGCCTAACAAGC-CAGGGGAA |  |
|  |  |  | W | GATGCCTAACAAGC-CAGGGGAA |  |
| Exp2R3G9 | S |  | W | GATGCCTAACAAGC-CAGGGGAG |  |
|  |  |  | W | GATGCCTAACAAGC-CAGGGGAG |  |
|  | T |  | W | GATGCCTAACAAGC-CAGGGGAA |  |
|  |  |  | W | GATGCCTAACAAGC-CAGGGGAA |  |
| Exp2R3G10 | S |  | W | GATGCCTAACAAGC-CAGGGGAG |  |
|  |  |  | W | GATGCCTAACAAGC-CAGGGGAG |  |
|  | T |  | W | GATGCCTAACAAGC-CAGGGGAA |  |
|  |  |  | W | GATGCCTAACAAGC-CAGGGGAA |  |
| Exp2R3G11 | S | Hetero | W | GATGCCTAACAAGC--AGGGGAG |  |
|  |  |  | I | GATGCCTAACAAGCGCAGGGGAG |  |
|  | T |  | W | GATGCCTAACAAGC-CAGGGGAA |  |
|  |  |  | W | GATGCCTAACAAGC-CAGGGGAA |  |
| Exp2R3G12 | S | Homo | I | GATGCCTAACAAGCGCAGGGGAG |  |
|  |  |  | I | GATGCCTAACAAGCGCAGGGGAG |  |
|  | T | Hetero | W | GATGCCTAACAAGC-CAGGGGAA |  |
|  |  |  | I | GATGCCTAACAAGCTCAGGGGAA |  |
| Exp2R3G13 | S |  | W | GATGCCTAACAAGC-CAGGGGAG |  |
|  |  |  | W | GATGCCTAACAAGC-CAGGGGAG |  |
|  | T |  | W | GATGCCTAACAAGC-CAGGGGAA |  |
|  |  |  | W | GATGCCTAACAAGC-CAGGGGAA |  |
| Exp2R3G14 | S | Hetero | I | GATGCCTAACAAGCGCAGGGGAG |  |
|  |  |  | I | GATGCCTAACAAGCTCAGGGGAG |  |
|  | T | Hetero | W | GATGCCTAACAAGC-CAGGGGAA |  |
|  |  |  | I | GATGCCTAACAAGCACAGGGGAA |  |
| Exp2R3G15 | S |  | W | GATGCCTAACAAGC-CAGGGGAG |  |
|  |  |  | W | GATGCCTAACAAGC-CAGGGGAG |  |
|  | T |  | W | GATGCCTAACAAGC-CAGGGGAA |  |
|  |  |  | W | GATGCCTAACAAGC-CAGGGGAA |  |
| Exp2R3G16 | S |  | W | GATGCCTAACAAGC-CAGGGGAG |  |
|  |  |  | W | GATGCCTAACAAGC-CAGGGGAG |  |
|  | T |  | W | GATGCCTAACAAGC-CAGGGGAA |  |
|  |  |  | W | GATGCCTAACAAGC-CAGGGGAA |  |
| Exp2R3G17 | S | Hetero | W | GATGCCTAACAAGC-CAGGGGAG |  |
|  |  |  | D | GATGCCTAACAAGC-(-11 bps) |  |
|  | T | Hetero | W | GATGCCTAACAAGC-CAGGGGAA |  |
|  |  |  | I | GATGCCTAACAAGCGCAGGGGAA |  |
| Exp2R3G18 | S | Hetero | W | GATGCCTAACAAGC-CAGGGGAG |  |
|  |  |  | I | GATGCCTAACAAGCACAGGGGAG |  |
|  | T | Homo | I | GATGCCTAACAAGCCCAGGGGAA |  |
|  |  |  | I | GATGCCTAACAAGCCCAGGGGAA |  |
| Exp2R3G19 | S | Homo | I | GATGCCTAACAAGCACAGGGGAG |  |
|  |  |  | I | GATGCCTAACAAGCACAGGGGAG |  |
|  | T | Hetero | W | GATGCCTAACAAGC-CAGGGGAA |  |
|  |  |  | I | GATGCCTAACAAGCTCAGGGGAA |  |
| Exp2R3G20 | S |  | W | GATGCCTAACAAGC-CAGGGGAG |  |
|  |  | Hetero | I | GATGCCTAACAAGCGCAGGGGAG |  |
|  | T |  | W | GATGCCTAACAAGC-CAGGGGAA |  |
|  |  |  | W | GATGCCTAACAAGC-CAGGGGAA |  |

| **Sample no.** | **Genome type** | **genotype** | **Mutation type** | ***NtPDS* sequences** |  |
| --- | --- | --- | --- | --- | --- |
| Wild type | S |  | W | GATGCCTAACAAGC-CAGGGGAG |  |
|  | T |  | W | GATGCCTAACAAGC-CAGGGGAA |  |
|  | | | | | |
| Exp3R1A1 | S | Homo | I | GATGCCTAACAAGCTCAGGGGAG |  |
|  |  |  | I | GATGCCTAACAAGCTCAGGGGAG |  |
|  | T | Hetero | D | GATGCCTAACAAGC--AGGGGAA |  |
|  |  |  | I | GATGCCTAACAAGCTCAGGGGAA |  |
| Exp3R1A2 | S | Hetero | I | GATGCCTAACAAGCGCAGGGGAG |  |
|  |  |  | I | GATGCCTAACAAGCTCAGGGGAG |  |
|  | T | Hetero | I | GATGCCTAACAAGCTCAGGGGAA |  |
|  |  |  | I | GATGCCTAACAAGCACAGGGGAA |  |
| Exp3R1A3 | S | Hetero | D | GATGCCTAACAAGC-(-7 bps) |  |
|  |  |  | I | GATGCCTAACAAGCCCAGGGGAG |  |
|  | T | Hetero | I | GATGCCTAACAAGCTCAGGGGAA |  |
|  |  |  | D | GATGCCTAACAAGC--AGGGGAA |  |
| Exp3R1A4 | S | Hetero | I | GATGCCTAACAAGCTCAGGGGAG |  |
|  |  |  | I | GATGCCTAACAAGCACAGGGGAG |  |
|  | T | Hetero | I | GATGCCTAACAAGCCCAGGGGAA |  |
|  |  |  | I | GATGCCTAACAAGCACAGGGGAA |  |
| Exp3R1A5 | S | Hetero | I | GATGCCTAACAAGC(+160 bps) |  |
|  |  |  | I | GATGCCTAACAAGCGCAGGGGAG |  |
|  | T | Hetero | I | GATGCCTAACAAGCGCAGGGGAA |  |
|  |  |  | I | GATGCCTAACAAGC(+43 bps) |  |
| Exp3R1A6 | S | Hetero | I | GATGCCTAACAAGCGCAGGGGAG |  |
|  |  |  | D | GATGCCTAACAAGC--AGGGGAG |  |
|  | T | Homo | I | GATGCCTAACAAGCTCAGGGGAA |  |
|  |  |  | I | GATGCCTAACAAGCTCAGGGGAA |  |
| Exp3R1A7 | S | Homo | I | GATGCCTAACAAGCTCAGGGGAG |  |
|  |  |  | I | GATGCCTAACAAGCTCAGGGGAG |  |
|  | T | Homo | I | GATGCCTAACAAGCTCAGGGGAA |  |
|  |  |  | I | GATGCCTAACAAGCTCAGGGGAA |  |
| Exp3R1A8 | S | Hetero | I | GATGCCTAACAAGCACAGGGGAG |  |
|  |  |  | I | GATGCCTAACAAGCACAGGGGAG |  |
|  | T | Hetero | I | GATGCCTAACAAGCACAGGGGAA |  |
|  |  |  | I | GATGCCTAACAAGCGCAGGGGAA |  |
| Exp3R1A9 | S | Hetero | I | GATGCCTAACAAGCACAGGGGAG |  |
|  |  |  | I | GATGCCTAACAAGCTCAGGGGAG |  |
|  | T | Hetero | I | GATGCCTAACAAGCACAGGGGAA |  |
|  |  |  | I | GATGCCTAACAAGCTCAGGGGAA |  |
| Exp3R1A10 | S | Hetero | I | GATGCCTAACAAGCACAGGGGAG |  |
|  |  |  | I | GATGCCTAACAAGCTCAGGGGAG |  |
|  | T | Hetero | I | GATGCCTAACAAGCACAGGGGAA |  |
|  |  |  | I | GATGCCTAACAAGCTCAGGGGAA |  |
| Exp3R1A11 | S | Homo | I | GATGCCTAACAAGCTCAGGGGAG |  |
|  |  |  | I | GATGCCTAACAAGCTCAGGGGAG |  |
|  | T | Hetero | I | GATGCCTAACAAGCTCAGGGGAA |  |
|  |  |  | I | GATGCCTAACAAGCGCAGGGGAA |  |
| Exp3R1A12 | S | Hetero | I | GATGCCTAACAAGCACAGGGGAG |  |
|  |  |  | I | GATGCCTAACAAGCACAGGGGAG |  |
|  | T | Hetero | I | GATGCCTAACAAGCTCAGGGGAA |  |
|  |  |  | I | GATGCCTAACAAGCGCAGGGGAA |  |
| Exp3R1A13 | S | Hetero | I | GATGCCTAACAAGCGCAGGGGAG |  |
|  |  |  | I | GATGCCTAACAAGCTCAGGGGAG |  |
|  | T | Hetero | I | GATGCCTAACAAGCACAGGGGAA |  |
|  |  |  | I | GATGCCTAACAAGCTCAGGGGAA |  |
| Exp3R1A14 | S | Hetero | I | GATGCCTAACAAGC(+82 bps) |  |
|  |  |  | I | GATGCCTAACAAGCTCAGGGGAG |  |
|  | T | Hetero | I | GATGCCTAACAAGCGCAGGGGAA |  |
|  |  |  | I | GATGCCTAACAAGCTCAGGGGAA |  |
| Exp3R1A15 | S | Homo | I | GATGCCTAACAAGCACAGGGGAG |  |
|  |  |  | I | GATGCCTAACAAGCACAGGGGAG |  |
|  | T | Hetero | I | GATGCCTAACAAGCACAGGGGAA |  |
|  |  |  | I | GATGCCTAACAAGCGCAGGGGAA |  |
| Exp3R1A16 | S | Hetero | D | GATGCCTAACAAGC--AGGGGAG |  |
|  |  |  | I | GATGCCTAACAAGCTCAGGGGAG |  |
|  | T | Hetero | I | GATGCCTAACAAGCACAGGGGAA |  |
|  |  |  | I | GATGCCTAACAAGCTCAGGGGAA |  |
| Exp3R1A17 | S | Hetero | I | GATGCCTAACAAGCACAGGGGAG |  |
|  |  |  | I | GATGCCTAACAAGCACAGGGGAG |  |
|  | T | Homo | I | GATGCCTAACAAGCTCAGGGGAA |  |
|  |  |  | I | GATGCCTAACAAGCTCAGGGGAA |  |
| Exp3R1A18 | S | Hetero | I | GATGCCTAACAAGCACAGGGGAG |  |
|  |  |  | I | GATGCCTAACAAGCTCAGGGGAG |  |
|  | T | Hetero | I | GATGCCTAACAAGCGCAGGGGAA |  |
|  |  |  | I | GATGCCTAACAAGCGCAGGGGAA |  |
| Exp3R1A19 | S | Hetero | D | GATGCCTAACAAGC--AGGGGAG |  |
|  |  |  | D | GATGCCTAACAAGC---GGGGAG |  |
|  | T | Hetero | I | GATGCCTAACAAGCCCAGGGGAA |  |
|  |  |  | I | GATGCCTAACAAGCTCAGGGGAA |  |
| Exp3R1A20 | S | Homo | I | GATGCCTAACAAGCTCAGGGGAG |  |
|  |  |  | I | GATGCCTAACAAGCTCAGGGGAG |  |
|  | T | Hetero | I | GATGCCTAACAAGCGCAGGGGAA |  |
|  |  |  | I | GATGCCTAACAAGCTCAGGGGAA |  |
|  | | | | | |
| Exp3R1G1 | S | Hetero | W | GATGCCTAACAAGC-CAGGGGAG |  |
|  |  |  | I | GATGCCTAACAAGCACAGGGGAG |  |
|  | T | Hetero | W | GATGCCTAACAAGC-CAGGGGAA |  |
|  |  |  | I | GATGCCTAACAAGCTCAGGGGAA |  |
| Exp3R1G2 | S |  | W | GATGCCTAACAAGC-CAGGGGAG |  |
|  |  |  | W | GATGCCTAACAAGC-CAGGGGAG |  |
|  | T |  | W | GATGCCTAACAAGC-CAGGGGAA |  |
|  |  |  | W | GATGCCTAACAAGC-CAGGGGAA |  |
| Exp3R1G3 | S |  | W | GATGCCTAACAAGC-CAGGGGAG |  |
|  |  |  | W | GATGCCTAACAAGC-CAGGGGAG |  |
|  | T |  | W | GATGCCTAACAAGC-CAGGGGAA |  |
|  |  |  | W | GATGCCTAACAAGC-CAGGGGAA |  |
| Exp3R1G4 | S | Hetero | W | GATGCCTAACAAGC-CAGGGGAG |  |
|  |  |  | D | GATGCCTAACAAGC(-11 bps) |  |
|  | T | Hetero | W | GATGCCTAACAAGC-CAGGGGAA |  |
|  |  |  | I | GATGCCTAACAAGCTCAGGGGAA |  |
| Exp3R1G5 | S | Homo | I | GATGCCTAACAAGCTCAGGGGAG |  |
|  |  |  | I | GATGCCTAACAAGCTCAGGGGAG |  |
|  | T | Hetero | W | GATGCCTAACAAGC-CAGGGGAA |  |
|  |  |  | I | GATGCCTAACAAGCTCAGGGGAA |  |
| Exp3R1G6 | S |  | W | GATGCCTAACAAGC-CAGGGGAG |  |
|  |  |  | W | GATGCCTAACAAGC-CAGGGGAG |  |
|  | T |  | W | GATGCCTAACAAGC-CAGGGGAA |  |
|  |  |  | W | GATGCCTAACAAGC-CAGGGGAA |  |
| Exp3R1G7 | S |  | W | GATGCCTAACAAGC-CAGGGGAG |  |
|  |  |  | W | GATGCCTAACAAGC-CAGGGGAG |  |
|  | T |  | W | GATGCCTAACAAGC-CAGGGGAA |  |
|  |  |  | W | GATGCCTAACAAGC-CAGGGGAA |  |
| Exp3R1G8 | S | Hetero | I | GATGCCTAACAAGCACAGGGGAG |  |
|  |  |  | I | GATGCCTAACAAGCTCAGGGGAG |  |
|  | T |  | W | GATGCCTAACAAGC-CAGGGGAA |  |
|  |  |  | W | GATGCCTAACAAGC-CAGGGGAA |  |
| Exp3R1G9 | S |  | W | GATGCCTAACAAGC-CAGGGGAG |  |
|  |  |  | W | GATGCCTAACAAGC-CAGGGGAG |  |
|  | T |  | W | GATGCCTAACAAGC-CAGGGGAA |  |
|  |  |  | W | GATGCCTAACAAGC-CAGGGGAA |  |
| Exp3R1G10 | S | Hetero | I | GATGCCTAACAAGCCCAGGGGAG |  |
|  |  |  | I | GATGCCTAACAAGCTCAGGGGAG |  |
|  | T |  | W | GATGCCTAACAAGC-CAGGGGAA |  |
|  |  |  | W | GATGCCTAACAAGC-CAGGGGAA |  |
| Exp3R1G11 | S |  | W | GATGCCTAACAAGC-CAGGGGAG |  |
|  |  |  | W | GATGCCTAACAAGC-CAGGGGAG |  |
|  | T |  | W | GATGCCTAACAAGC-CAGGGGAA |  |
|  |  |  | W | GATGCCTAACAAGC-CAGGGGAA |  |
| Exp3R1G12 | S | Hetero | I | GATGCCTAACAAGCGCAGGGGAG |  |
|  |  |  | I | GATGCCTAACAAGCACAGGGGAG |  |
|  | T | Hetero | W | GATGCCTAACAAGC-CAGGGGAA |  |
|  |  |  | I | GATGCCTAACAAGCTCAGGGGAA |  |
| Exp3R1G13 | S | Hetero | W | GATGCCTAACAAGC-CAGGGGAG |  |
|  |  |  | I | GATGCCTAACAAGCTCAGGGGAG |  |
|  | T |  | W | GATGCCTAACAAGC-CAGGGGAA |  |
|  |  |  | W | GATGCCTAACAAGC-CAGGGGAA |  |
| Exp3R1G14 | S | Homo | I | GATGCCTAACAAGCACAGGGGAG |  |
|  |  |  | I | GATGCCTAACAAGCACAGGGGAG |  |
|  | T | Hetero | W | GATGCCTAACAAGC-CAGGGGAA |  |
|  |  |  | I | GATGCCTAACAAGCTCAGGGGAA |  |
| Exp3R1G15 | S | Hetero | W | GATGCCTAACAAGC-CAGGGGAG |  |
|  |  |  | I | GATGCCTAACAAGCTCAGGGGAG |  |
|  | T |  | W | GATGCCTAACAAGC-CAGGGGAA |  |
|  |  |  | W | GATGCCTAACAAGC-CAGGGGAA |  |
| Exp3R1G16 | S | Hetero | I | GATGCCTAACAAGCCCAGGGGAG |  |
|  |  |  | I | GATGCCTAACAAGCTCAGGGGAG |  |
|  | T | Hetero | W | GATGCCTAACAAGC-CAGGGGAA |  |
|  |  |  | I | GATGCCTAACAAGCTCAGGGGAA |  |
| Exp3R1G17 | S |  | W | GATGCCTAACAAGC-CAGGGGAG |  |
|  |  |  | W | GATGCCTAACAAGC-CAGGGGAG |  |
|  | T |  | W | GATGCCTAACAAGC-CAGGGGAA |  |
|  |  |  | W | GATGCCTAACAAGC-CAGGGGAA |  |
| Exp3R1G18 | S |  | W | GATGCCTAACAAGC-CAGGGGAG |  |
|  |  |  | W | GATGCCTAACAAGC-CAGGGGAG |  |
|  | T |  | W | GATGCCTAACAAGC-CAGGGGAA |  |
|  |  |  | W | GATGCCTAACAAGC-CAGGGGAA |  |
| Exp3R1G19 | S | Homo | I | GATGCCTAACAAGCGCAGGGGAG |  |
|  |  |  | I | GATGCCTAACAAGCGCAGGGGAG |  |
|  | T |  | W | GATGCCTAACAAGC-CAGGGGAA |  |
|  |  |  | W | GATGCCTAACAAGC-CAGGGGAA |  |
| Exp3R1G20 | S |  | W | GATGCCTAACAAGC-CAGGGGAG |  |
|  |  |  | W | GATGCCTAACAAGC-CAGGGGAG |  |
|  | T |  | W | GATGCCTAACAAGC-CAGGGGAA |  |
|  |  |  | W | GATGCCTAACAAGC-CAGGGGAA |  |

| **Sample no.** | **Genome type** | **genotype** | **Mutation type** | ***NtPDS* sequences** |  |
| --- | --- | --- | --- | --- | --- |
| Wild type | S |  | W | GATGCCTAACAAGC-CAGGGGAG |  |
|  | T |  | W | GATGCCTAACAAGC-CAGGGGAA |  |
|  | | | | |  |
| Exp3R2A1 | S | Homo | I | GATGCCTAACAAGCTCAGGGGAG |  |
|  |  |  | I | GATGCCTAACAAGCTCAGGGGAG |  |
|  | T | Hetero | I | GATGCCTAACAAGCTCAGGGGAA |  |
|  |  |  | I | GATGCCTAACAAGCACAGGGGAA |  |
| Exp3R2A2 | S | Homo | I | GATGCCTAACAAGCACAGGGGAG |  |
|  |  |  | I | GATGCCTAACAAGCACAGGGGAG |  |
|  | T | Homo | I | GATGCCTAACAAGCACAGGGGAA |  |
|  |  |  | I | GATGCCTAACAAGCACAGGGGAA |  |
| Exp3R2A3 | S | Hetero | I | GATGCCTAACAAGCACAGGGGAG |  |
|  |  |  | I | GATGCCTAACAAGCTCAGGGGAG |  |
|  | T | Hetero | I | GATGCCTAACAAGCACAGGGGAA |  |
|  |  |  | I | GATGCCTAACAAGCTCAGGGGAA |  |
| Exp3R2A4 | S | Hetero | D | GATGCCTAACAAGC---GGGGAG |  |
|  |  |  | I | GATGCCTAACAAGCGCAGGGGAG |  |
|  | T | Hetero | I | GATGCCTAACAAGCACAGGGGAA |  |
|  |  |  | I | GATGCCTAACAAGCGCAGGGGAA |  |
| Exp3R2A5 | S | Homo | I | GATGCCTAACAAGCTCAGGGGAG |  |
|  |  |  | I | GATGCCTAACAAGCTCAGGGGAG |  |
|  | T | Hetero | D | GATGCCTAACAA---CAGGGGAA |  |
|  |  |  | I | GATGCCTAACAAGCTCAGGGGAA |  |
| Exp3R2A6 | S | Hetero | I | GATGCCTAACAAGCACAGGGGAG |  |
|  |  |  | I | GATGCCTAACAAGCTCAGGGGAG |  |
|  | T | Hetero | I | GATGCCTAACAAGCTCAGGGGAA |  |
|  |  |  | I | GATGCCTAACAAGCGCAGGGGAA |  |
| Exp3R2A7 | S | Hetero | I | GATGCCTAACAAGCGCAGGGGAG |  |
|  |  |  | I | GATGCCTAACAAGCACAGGGGAG |  |
|  | T | Hetero | I | GATGCCTAACAAGCACAGGGGAA |  |
|  |  |  | I | GATGCCTAACAAGCTCAGGGGAA |  |
| Exp3R2A8 | S | Homo | I | GATGCCTAACAAGCACAGGGGAG |  |
|  |  |  | I | GATGCCTAACAAGCACAGGGGAG |  |
|  | T | Homo | I | GATGCCTAACAAGCTCAGGGGAA |  |
|  |  |  | I | GATGCCTAACAAGCTCAGGGGAA |  |
| Exp3R2A9 | S | Hetero | I | GATGCCTAACAAGCCCAGGGGAG |  |
|  |  |  | I | GATGCCTAACAAGCTCAGGGGAG |  |
|  | T | Hetero | I | GATGCCTAACAAGCCCAGGGGAA |  |
|  |  |  | I | GATGCCTAACAAGCTCAGGGGAA |  |
| Exp3R2A10 | S | Homo | I | GATGCCTAACAAGCACAGGGGAG |  |
|  |  |  | I | GATGCCTAACAAGCACAGGGGAG |  |
|  | T | Hetero | I | GATGCCTAACAAGCACAGGGGAA |  |
|  |  |  | I | GATGCCTAACAAGCTCAGGGGAA |  |
| Exp3R2A11 | S | Hetero | I | GATGCCTAACAAGCGCAGGGGAG |  |
|  |  |  | I | GATGCCTAACAAGCTCAGGGGAG |  |
|  | T | Hetero | I | GATGCCTAACAAGCACAGGGGAA |  |
|  |  |  | I | GATGCCTAACAAGCGCAGGGGAA |  |
| Exp3R2A12 | S | Homo | I | GATGCCTAACAAGCTCAGGGGAG |  |
|  |  |  | I | GATGCCTAACAAGCTCAGGGGAG |  |
|  | T | Hetero | I | GATGCCTAACAAGCGCAGGGGAA |  |
|  |  |  | I | GATGCCTAACAAGCTCAGGGGAA |  |
| Exp3R2A13 | S | Homo | I | GATGCCTAACAAGCTCAGGGGAG |  |
|  |  |  | I | GATGCCTAACAAGCTCAGGGGAG |  |
|  | T | Homo | I | GATGCCTAACAAGCGCAGGGGAA |  |
|  |  |  | I | GATGCCTAACAAGCGCAGGGGAA |  |
| Exp3R2A14 | S | Hetero | I | GATGCCTAACAAGCACAGGGGAG |  |
|  |  |  | I | GATGCCTAACAAGCGTGGGCCGA |  |
|  | T | Homo | I | GATGCCTAACAAGCTCAGGGGAA |  |
|  |  |  | I | GATGCCTAACAAGCTCAGGGGAA |  |
| Exp3R2A15 | S | Hetero | I | GATGCCTAACAAGCGCAGGGGAG |  |
|  |  |  | I | GATGCCTAACAAGCTCAGGGGAG |  |
|  | T | Hetero | I | GATGCCTAACAAGCTCAGGGGAA |  |
|  |  |  | I | GATGCCTAACAAGC(+213 bps) |  |
| Exp3R2A16 | S | Homo | I | GATGCCTAACAAGCACAGGGGAG |  |
|  |  |  | I | GATGCCTAACAAGCACAGGGGAG |  |
|  | T | Hetero | I | GATGCCTAACAAGCACAGGGGAA |  |
|  |  |  | I | GATGCCTAACAAGCTCAGGGGAA |  |
| Exp3R2A17 | S | Homo | I | GATGCCTAACAAGCGCAGGGGAG |  |
|  |  |  | I | GATGCCTAACAAGCGCAGGGGAG |  |
|  | T | Hetero | I | GATGCCTAACAAGCACAGGGGAA |  |
|  |  |  | I | GATGCCTAACAAGCTCAGGGGAA |  |
| Exp3R2A18 | S | Homo | I | GATGCCTAACAAGCTCAGGGGAG |  |
|  |  |  | I | GATGCCTAACAAGCTCAGGGGAG |  |
|  | T | Homo | I | GATGCCTAACAAGCTCAGGGGAA |  |
|  |  |  | I | GATGCCTAACAAGCTCAGGGGAA |  |
| Exp3R2A19 | S | Homo | I | GATGCCTAACAAGCGCAGGGGAG |  |
|  |  |  | I | GATGCCTAACAAGCGCAGGGGAG |  |
|  | T | Hetero | I | GATGCCTAACAAGCACAGGGGAA |  |
|  |  |  | I | GATGCCTAACAAGCTCAGGGGAA |  |
| Exp3R2A20 | S | Homo | D | GATGCCTAACAAGC--AGGGGAG |  |
|  |  |  | D | GATGCCTAACAAGC--AGGGGAG |  |
|  | T | Homo | I | GATGCCTAACAAGCACAGGGGAA |  |
|  |  |  | I | GATGCCTAACAAGCACAGGGGAA |  |
|  | | | | | |
| Exp3R2G1 | S | Hetero | I | GATGCCTAACAAGCTCAGGGGAG |  |
|  |  |  | I | GATGCCTAACAAGCGCAGGGGAG |  |
|  | T | Hetero | W | GATGCCTAACAAGC-CAGGGGAA |  |
|  |  |  | I | GATGCCTAACAAGCACAGGGGAA |  |
| Exp3R2G2 | S | Hetero | I | GATGCCTAACAAGCTCAGGGGAG |  |
|  |  |  | I | GATGCCTAACAAGCGCAGGGGAG |  |
|  | T | Hetero | W | GATGCCTAACAAGC-CAGGGGAA |  |
|  |  |  | I | GATGCCTAACAAGCTCAGGGGAA |  |
| Exp3R2G3 | S | Homo | I | GATGCCTAACAAGCACAGGGGAG |  |
|  |  |  | I | GATGCCTAACAAGCACAGGGGAG |  |
|  | T | Hetero | W | GATGCCTAACAAGC-CAGGGGAA |  |
|  |  |  | I | GATGCCTAACAAGCACAGGGGAA |  |
| Exp3R2G4 | S | Homo | I | GATGCCTAACAAGCTCAGGGGAG |  |
|  |  |  | I | GATGCCTAACAAGCTCAGGGGAG |  |
|  | T | Hetero | I | GATGCCTAACAAGCTCAGGGGAA |  |
|  |  |  | W | GATGCCTAACAAGC-CAGGGGAA |  |
| Exp3R2G5 | S | Hetero | I | GATGCCTAACAAGCACAGGGGAG |  |
|  |  |  | D | GATGCCTAACA------GGGGAG |  |
|  | T | Hetero | W | GATGCCTAACAAGC-CAGGGGAA |  |
|  |  |  | I | GATGCCTAACAAGCACAGGGGAA |  |
| Exp3R2G6 | S | Homo | I | GATGCCTAACAAGCTCAGGGGAG |  |
|  |  |  | I | GATGCCTAACAAGCTCAGGGGAG |  |
|  | T | Hetero | W | GATGCCTAACAAGC-CAGGGGAA |  |
|  |  |  | I | GATGCCTAACAAGCTCAGGGGAA |  |
| Exp3R2G7 | S |  | W | GATGCCTAACAAGC-CAGGGGAG |  |
|  |  |  | W | GATGCCTAACAAGC-CAGGGGAG |  |
|  | T |  | W | GATGCCTAACAAGC-CAGGGGAA |  |
|  |  |  | W | GATGCCTAACAAGC-CAGGGGAA |  |
| Exp3R2G8 | S | Homo | I | GATGCCTAACAAGCTCAGGGGAG |  |
|  |  |  | I | GATGCCTAACAAGCTCAGGGGAG |  |
|  | T |  | W | GATGCCTAACAAGC-CAGGGGAA |  |
|  |  |  | W | GATGCCTAACAAGC-CAGGGGAA |  |
| Exp3R2G9 | S |  | W | GATGCCTAACAAGC-CAGGGGAG |  |
|  |  |  | W | GATGCCTAACAAGC-CAGGGGAG |  |
|  | T |  | W | GATGCCTAACAAGC-CAGGGGAA |  |
|  |  |  | W | GATGCCTAACAAGC-CAGGGGAA |  |
| Exp3R2G10 | S |  | W | GATGCCTAACAAGC-CAGGGGAG |  |
|  |  |  | W | GATGCCTAACAAGC-CAGGGGAG |  |
|  | T |  | W | GATGCCTAACAAGC-CAGGGGAA |  |
|  |  |  | W | GATGCCTAACAAGC-CAGGGGAA |  |
| Exp3R2G11 | S | Hetero | I | GATGCCTAACAAGCGCAGGGGAG |  |
|  |  |  | I | GATGCCTAACAAGCACAGGGGAG |  |
|  | T | Hetero | W | GATGCCTAACAAGC-CAGGGGAA |  |
|  |  |  | I | GATGCCTAACAAGCTCAGGGGAA |  |
| Exp3R2G12 | S | Hetero | W | GATGCCTAACAAGC-CAGGGGAG |  |
|  |  |  | I | GATGCCTAACAAGCTCAGGGGAG |  |
|  | T | Hetero | W | GATGCCTAACAAGC-CAGGGGAA |  |
|  |  |  | I | GATGCCTAACAAGCCCAGGGGAA |  |
| Exp3R2G13 | S |  | W | GATGCCTAACAAGC-CAGGGGAG |  |
|  |  |  | W | GATGCCTAACAAGC-CAGGGGAG |  |
|  | T |  | W | GATGCCTAACAAGC-CAGGGGAA |  |
|  |  |  | W | GATGCCTAACAAGC-CAGGGGAA |  |
| Exp3R2G14 | S |  | W | GATGCCTAACAAGC-CAGGGGAG |  |
|  |  |  | W | GATGCCTAACAAGC-CAGGGGAG |  |
|  | T |  | W | GATGCCTAACAAGC-CAGGGGAA |  |
|  |  |  | W | GATGCCTAACAAGC-CAGGGGAA |  |
| Exp3R2G15 | S |  | W | GATGCCTAACAAGC-CAGGGGAG |  |
|  |  |  | W | GATGCCTAACAAGC-CAGGGGAG |  |
|  | T |  | W | GATGCCTAACAAGC-CAGGGGAA |  |
|  |  |  | W | GATGCCTAACAAGC-CAGGGGAA |  |
| Exp3R2G16 | S |  | W | GATGCCTAACAAGC-CAGGGGAG |  |
|  |  |  | W | GATGCCTAACAAGC-CAGGGGAG |  |
|  | T |  | W | GATGCCTAACAAGC-CAGGGGAA |  |
|  |  |  | W | GATGCCTAACAAGC-CAGGGGAA |  |
| Exp3R2G17 | S |  | W | GATGCCTAACAAGC-CAGGGGAG |  |
|  |  |  | W | GATGCCTAACAAGC-CAGGGGAG |  |
|  | T |  | W | GATGCCTAACAAGC-CAGGGGAA |  |
|  |  |  | W | GATGCCTAACAAGC-CAGGGGAA |  |
| Exp3R2G18 | S |  | W | GATGCCTAACAAGC-CAGGGGAG |  |
|  |  |  | W | GATGCCTAACAAGC-CAGGGGAG |  |
|  | T |  | W | GATGCCTAACAAGC-CAGGGGAA |  |
|  |  |  | W | GATGCCTAACAAGC-CAGGGGAA |  |
| Exp3R2G19 | S |  | W | GATGCCTAACAAGC-CAGGGGAG |  |
|  |  |  | W | GATGCCTAACAAGC-CAGGGGAG |  |
|  | T |  | W | GATGCCTAACAAGC-CAGGGGAA |  |
|  |  |  | W | GATGCCTAACAAGC-CAGGGGAA |  |
| Exp3R2G20 | S | Homo | I | GATGCCTAACAAGCTCAGGGGAG |  |
|  |  |  | I | GATGCCTAACAAGCTCAGGGGAG |  |
|  | T | Hetero | W | GATGCCTAACAAGC-CAGGGGAA |  |
|  |  |  | D | GATGCCTAACA----CAGGGGAA |  |

| **Sample no.** | **Genome type** | **genotype** | **Mutation type** | ***NtPDS* sequences** |  |
| --- | --- | --- | --- | --- | --- |
| Wild type | S |  | W | GATGCCTAACAAGC-CAGGGGAG |  |
|  | T |  | W | GATGCCTAACAAGC-CAGGGGAA |  |
|  | | | | | |
| Exp3R3A1 | S | Hetero | I | GATGCCTAACAAGCACAGGGGAG |  |
|  |  |  | I | GATGCCTAACAAGCGCAGGGGAG |  |
|  | T | Hetero | I | GATGCCTAACAAGCCCAGGGGAA |  |
|  |  |  | I | GATGCCTAACAAGCACAGGGGAA |  |
| Exp3R3A2 | S | Hetero | I | GATGCCTAACAAGCTCAGGGGAG |  |
|  |  |  | I | GATGCCTAACAAGCGCAGGGGAG |  |
|  | T | Hetero | I | GATGCCTAACAAGCTCAGGGGAA |  |
|  |  |  | I | GATGCCTAACAAGCGCAGGGGAA |  |
| Exp3R3A3 | S | Homo | I | GATGCCTAACAAGCACAGGGGAG |  |
|  |  |  | I | GATGCCTAACAAGCACAGGGGAG |  |
|  | T | Homo | I | GATGCCTAACAAGCTCAGGGGAA |  |
|  |  |  | I | GATGCCTAACAAGCTCAGGGGAA |  |
| Exp3R3A4 | S | Homo | I | GATGCCTAACAAGCACAGGGGAG |  |
|  |  |  | I | GATGCCTAACAAGCACAGGGGAG |  |
|  | T | Hetero | I | GATGCCTAACAAGCGCAGGGGAA |  |
|  |  |  | I | GATGCCTAACAAGCTCAGGGGAA |  |
| Exp3R3A5 | S | Hetero | I | GATGCCTAACAAGCACAGGGGAG |  |
|  |  |  | I | GATGCCTAACAAGCTCAGGGGAG |  |
|  | T | Hetero | I | GATGCCTAACAAGCTCAGGGGAA |  |
|  |  |  | I | GATGCCTAACAAGCGCAGGGGAA |  |
| Exp3R3A6 | S | Hetero | I | GATGCCTAACAAGCACAGGGGAG |  |
|  |  |  | I | GATGCCTAACAAGCTCAGGGGAG |  |
|  | T | Homo | I | GATGCCTAACAAGCTCAGGGGAA |  |
|  |  |  | I | GATGCCTAACAAGCTCAGGGGAA |  |
| Exp3R3A7 | S | Hetero | I | GATGCCTAACAAGCACAGGGGAG |  |
|  |  |  | I | GATGCCTAACAAGCTCAGGGGAG |  |
|  | T | Hetero | I | GATGCCTAACAAGCTCAGGGGAA |  |
|  |  |  | I | GATGCCTAACAAGCGCAGGGGAA |  |
| Exp3R3A8 | S | Hetero | I | GATGCCTAACAAGCCCAGGGGAG |  |
|  |  |  | I | GATGCCTAACAAGCTCAGGGGAG |  |
|  | T | Hetero | I | GATGCCTAACAAGCACAGGGGAA |  |
|  |  |  | I | GATGCCTAACAAGCGCAGGGGAA |  |
| Exp3R3A9 | S | Hetero | I | GATGCCTAACAAGCACAGGGGAG |  |
|  |  |  | I | GATGCCTAACAAGCGCAGGGGAG |  |
|  | T | Homo | I | GATGCCTAACAAGCTCAGGGGAA |  |
|  |  |  | I | GATGCCTAACAAGCTCAGGGGAA |  |
| Exp3R3A10 | S | Hetero | I | GATGCCTAACAAGCCCAGGGGAG |  |
|  |  |  | I | GATGCCTAACAAGCTCAGGGGAG |  |
|  | T | Hetero | I | GATGCCTAACAAGCACAGGGGAA |  |
|  |  |  | I | GATGCCTAACAAGCGCAGGGGAA |  |
| Exp3R3A11 | S | Hetero | D | GATGCCTAACAAGC--AGGGGAG |  |
|  |  |  | I | GATGCCTAACAAGCACAGGGGAG |  |
|  | T | Hetero | I | GATGCCTAACAAGCACAGGGGAA |  |
|  |  |  | I | GATGCCTAACAAGCAACAGGGGA |  |
| Exp3R3A12 | S | Hetero | I | GATGCCTAACAAGCCCAGGGGAG |  |
|  |  |  | I | GATGCCTAACAAGCTCAGGGGAG |  |
|  | T | Hetero | I | GATGCCTAACAAGCACAGGGGAA |  |
|  |  |  | I | GATGCCTAACAAGCGCAGGGGAA |  |
| Exp3R3A13 | S | Hetero | I | GATGCCTAACAAGCACAGGGGAG |  |
|  |  |  | I | GATGCCTAACAAGCGCAGGGGAG |  |
|  | T | Homo | I | GATGCCTAACAAGCTCAGGGGAA |  |
|  |  |  | I | GATGCCTAACAAGCTCAGGGGAA |  |
| Exp3R3A14 | S | Homo | I | GATGCCTAACAAGCACAGGGGAG |  |
|  |  |  | I | GATGCCTAACAAGCACAGGGGAG |  |
|  | T | Hetero | I | GATGCCTAACAAGCACAGGGGAA |  |
|  |  |  | I | GATGCCTAACAAGCTCAGGGGAA |  |
| Exp3R3A15 | S | Hetero | I | GATGCCTAACAAGCACAGGGGAG |  |
|  |  |  | D | GATGCCTAACAA-----GGGGAG |  |
|  | T | Homo | I | GATGCCTAACAAGCACAGGGGAA |  |
|  |  |  | I | GATGCCTAACAAGCACAGGGGAA |  |
| Exp3R3A16 | S | Homo | I | GATGCCTAACAAGCTCAGGGGAG |  |
|  |  |  | I | GATGCCTAACAAGCTCAGGGGAG |  |
|  | T | Homo | I | GATGCCTAACAAGCTCAGGGGAA |  |
|  |  |  | I | GATGCCTAACAAGCTCAGGGGAA |  |
| Exp3R3A17 | S | Homo | I | GATGCCTAACAAGCACAGGGGAG |  |
|  |  |  | I | GATGCCTAACAAGCACAGGGGAG |  |
|  | T | Homo | I | GATGCCTAACAAGCTCAGGGGAA |  |
|  |  |  | I | GATGCCTAACAAGCTCAGGGGAA |  |
| Exp3R3A18 | S | Homo | I | GATGCCTAACAAGCACAGGGGAG |  |
|  |  |  | I | GATGCCTAACAAGCACAGGGGAG |  |
|  | T | Homo | I | GATGCCTAACAAGCACAGGGGAA |  |
|  |  |  | I | GATGCCTAACAAGCACAGGGGAA |  |
| Exp3R3A19 | S | Homo | I | GATGCCTAACAAGCTCAGGGGAG |  |
|  |  |  | I | GATGCCTAACAAGCTCAGGGGAG |  |
|  | T | Hetero | I | GATGCCTAACAAGCACAGGGGAA |  |
|  |  |  | I | GATGCCTAACAAGCTCAGGGGAA |  |
| Exp3R3A20 | S | Homo | I | GATGCCTAACAAGCACAGGGGAG |  |
|  |  |  | I | GATGCCTAACAAGCACAGGGGAG |  |
|  | T | Hetero | I | GATGCCTAACAAGCACAGGGGAA |  |
|  |  |  | I | GATGCCTAACAAGCCAAGGGGAA |  |
|  | | | | | |
| Exp3R3G1 | S |  | W | GATGCCTAACAAGC-CAGGGGAG |  |
|  |  |  | W | GATGCCTAACAAGC-CAGGGGAG |  |
|  | T |  | W | GATGCCTAACAAGC-CAGGGGAA |  |
|  |  |  | W | GATGCCTAACAAGC-CAGGGGAA |  |
| Exp3R3G2 | S |  | W | GATGCCTAACAAGC-CAGGGGAG |  |
|  |  |  | W | GATGCCTAACAAGC-CAGGGGAG |  |
|  | T |  | W | GATGCCTAACAAGC-CAGGGGAA |  |
|  |  |  | W | GATGCCTAACAAGC-CAGGGGAA |  |
| Exp3R3G3 | S | Hetero | I | GATGCCTAACAAGCTCAGGGGAG |  |
|  |  |  | W | GATGCCTAACAAGC-CAGGGGAG |  |
|  | T | Hetero | I | GATGCCTAACAAGC-CAGGGGAA |  |
|  |  |  | W | GATGCCTAACAAGCTCAGGGGAA |  |
| Exp3R3G4 | S | Hetero | I | GATGCCTAACAAGCTCAGGGGAG |  |
|  |  |  | I | GATGCCTAACAAGCACAGGGGAG |  |
|  | T | Hetero | W | GATGCCTAACAAGC-CAGGGGAA |  |
|  |  |  | I | GATGCCTAACAAGCTCAGGGGAA |  |
| Exp3R3G5 | S | Hetero | W | GATGCCTAACAAGC-CAGGGGAG |  |
|  |  |  | I | GATGCCTAACAAGCACAGGGGAG |  |
|  | T | Hetero | W | GATGCCTAACAAGC-CAGGGGAA |  |
|  |  |  | I | GATGCCTAACAAGCTCAGGGGAA |  |
| Exp3R3G6 | S |  | W | GATGCCTAACAAGC-CAGGGGAG |  |
|  |  |  | W | GATGCCTAACAAGC-CAGGGGAG |  |
|  | T |  | W | GATGCCTAACAAGC-CAGGGGAA |  |
|  |  |  | W | GATGCCTAACAAGC-CAGGGGAA |  |
| Exp3R3G7 | S | Hetero | I | GATGCCTAACAAGCTCAGGGGAG |  |
|  |  |  | D | GATGCCTAACAAGC--AGGGGAG |  |
|  | T |  | W | GATGCCTAACAAGC-CAGGGGAA |  |
|  |  |  | W | GATGCCTAACAAGC-CAGGGGAA |  |
| Exp3R3G8 | S | Hetero | W | GATGCCTAACAAGC-CAGGGGAG |  |
|  |  |  | I | GATGCCTAACAAGCACAGGGGAG |  |
|  | T | Hetero | W | GATGCCTAACAAGC-CAGGGGAA |  |
|  |  |  | I | GATGCCTAACAAGCTCAGGGGAA |  |
| Exp3R3G9 | S | Hetero | W | GATGCCTAACAAGC-CAGGGGAG |  |
|  |  |  | I | GATGCCTAACAAGCTCAGGGGAG |  |
|  | T |  | W | GATGCCTAACAAGC-CAGGGGAA |  |
|  |  |  | W | GATGCCTAACAAGC-CAGGGGAA |  |
| Exp3R3G10 | S | Hetero | W | GATGCCTAACAAGC-GCAGGGGAG |  |
|  |  |  | I | GATGCCTAACAAGCAACAGGGGAG |  |
|  | T | Hetero | W | GATGCCTAACAAGC-CAGGGGAA |  |
|  |  |  | I | GATGCCTAACAAGCACAGGGGAA |  |
| Exp3R3G11 | S | Hetero | I | GATGCCTAACAAGCGCAGGGGAG |  |
|  |  |  | I | GATGCCTAACAAGCTCAGGGGAG |  |
|  | T | Hetero | W | GATGCCTAACAAGC-CAGGGGAA |  |
|  |  |  | I | GATGCCTAACAAGCACAGGGGAA |  |
| Exp3R3G12 | S |  | W | GATGCCTAACAAGC-CAGGGGAG |  |
|  |  |  | W | GATGCCTAACAAGC-CAGGGGAG |  |
|  | T |  | W | GATGCCTAACAAGC-CAGGGGAA |  |
|  |  |  | W | GATGCCTAACAAGC-CAGGGGAA |  |
| Exp3R3G13 | S |  | W | GATGCCTAACAAGC-CAGGGGAG |  |
|  |  |  | W | GATGCCTAACAAGC-CAGGGGAG |  |
|  | T |  | W | GATGCCTAACAAGC-CAGGGGAA |  |
|  |  |  | W | GATGCCTAACAAGC-CAGGGGAA |  |
| Exp3R3G14 | S |  | W | GATGCCTAACAAGC-CAGGGGAG |  |
|  |  |  | W | GATGCCTAACAAGC-CAGGGGAG |  |
|  | T |  | W | GATGCCTAACAAGC-CAGGGGAA |  |
|  |  |  | W | GATGCCTAACAAGC-CAGGGGAA |  |
| Exp3R3G15 | S |  | W | GATGCCTAACAAGC-CAGGGGAG |  |
|  |  |  | W | GATGCCTAACAAGC-CAGGGGAG |  |
|  | T |  | W | GATGCCTAACAAGC-CAGGGGAA |  |
|  |  |  | W | GATGCCTAACAAGC-CAGGGGAA |  |
| Exp3R3G16 | S |  | W | GATGCCTAACAAGC-CAGGGGAG |  |
|  |  |  | W | GATGCCTAACAAGC-CAGGGGAG |  |
|  | T |  | W | GATGCCTAACAAGC-CAGGGGAA |  |
|  |  |  | W | GATGCCTAACAAGC-CAGGGGAA |  |
| Exp3R3G17 | S |  | W | GATGCCTAACAAGC-CAGGGGAG |  |
|  |  |  | W | GATGCCTAACAAGC-CAGGGGAG |  |
|  | T |  | W | GATGCCTAACAAGC-CAGGGGAA |  |
|  |  |  | W | GATGCCTAACAAGC-CAGGGGAA |  |
| Exp3R3G18 | S | Hetero | I | GATGCCTAACAAGCTCAGGGGAG |  |
|  |  |  | I | GATGCCTAACAAGCACAGGGGAG |  |
|  | T |  | W | GATGCCTAACAAGC-CAGGGGAA |  |
|  |  |  | W | GATGCCTAACAAGC-CAGGGGAA |  |
| Exp3R3G19 | S | Hetero | I | GATGCCTAACAAGCTCAGGGGAG |  |
|  |  |  | W | GATGCCTAACAAGC-CAGGGGAG |  |
|  | T |  | W | GATGCCTAACAAGC-CAGGGGAA |  |
|  |  |  | W | GATGCCTAACAAGC-CAGGGGAA |  |
| Exp3R3G20 | S | Hetero | W | GATGCCTAACAAGC-CAGGGGAG |  |
|  |  |  | D | GATGCCTAACAAGC-(-17 bps) |  |
|  | T | Hetero | I | GATGCCTAACAAGCTCAGGGGAA |  |
|  |  |  | I | GATGCCTAACAAGCACAGGGGAA |  |
