# Supplementary material for: Application of protoplast technology to CRISPR/Cas9 mutagenesis: from single‐cell mutation detection to mutant plant regeneration
Source: Plant Biotechnol J. 2018 Jan 10;16(7):1295–310. doi: 10.1111/pbi.12870 (PMC5999315; doi:10.1111/pbi.12870)
Supplement: Supplementary file 7 — Data S5 The NtPDS sequences of Figure S17. [file PBI-16-1295-s004.docx]

**Supplemental Data 5. The *NtPDS* sequences of Supplemental Figure 17.** Red box in Sample no. column indicates four copies of *NtPDS* have same mutations. S: *sylvestris* form. T: *tomentosiformis* form. Orange box-Homo: homozygous in this form. Light blue box-hetero: heterozygous in this form. W: wild type. I: insertion. D: deletion. Letter in red: the mutated nucleotide.

| **Sample no.** | **Genome type** | **genotype** | **Mutation type** | ***NtPDS* sequences** |  |
| --- | --- | --- | --- | --- | --- |
| Wild type | S |  | W | GATGCCTAACAAGC-CAGGGGAG |  |
|  | T |  | W | GATGCCTAACAAGC-CAGGGGAA |  |
|  | | | | | |
| 0 µg R1-1 | S |  | W | GATGCCTAACAAGC-CAGGGGAG |  |
|  |  |  | W | GATGCCTAACAAGC-CAGGGGAG |  |
|  | T |  | W | GATGCCTAACAAGC-CAGGGGAA |  |
|  |  |  | W | GATGCCTAACAAGC-CAGGGGAA |  |
| 0 µg R1-2 | S |  | W | GATGCCTAACAAGC-CAGGGGAG |  |
|  |  |  | W | GATGCCTAACAAGC-CAGGGGAG |  |
|  | T |  | W | GATGCCTAACAAGC-CAGGGGAA |  |
|  |  |  | W | GATGCCTAACAAGC-CAGGGGAA |  |
| 0 µg R1-3 | S |  | W | GATGCCTAACAAGC-CAGGGGAG |  |
|  |  |  | W | GATGCCTAACAAGC-CAGGGGAG |  |
|  | T |  | W | GATGCCTAACAAGC-CAGGGGAA |  |
|  |  |  | W | GATGCCTAACAAGC-CAGGGGAA |  |
| 0 µg R1-4 | S |  | W | GATGCCTAACAAGC-CAGGGGAG |  |
|  |  |  | W | GATGCCTAACAAGC-CAGGGGAG |  |
|  | T |  | W | GATGCCTAACAAGC-CAGGGGAA |  |
|  |  |  | W | GATGCCTAACAAGC-CAGGGGAA |  |
| 0 µg R1-5 | S |  | W | GATGCCTAACAAGC-CAGGGGAG |  |
|  |  |  | W | GATGCCTAACAAGC-CAGGGGAG |  |
|  | T |  | W | GATGCCTAACAAGC-CAGGGGAA |  |
|  |  |  | W | GATGCCTAACAAGC-CAGGGGAA |  |
| 0 µg R1-6 | S |  | W | GATGCCTAACAAGC-CAGGGGAG |  |
|  |  |  | W | GATGCCTAACAAGC-CAGGGGAG |  |
|  | T |  | W | GATGCCTAACAAGC-CAGGGGAA |  |
|  |  |  | W | GATGCCTAACAAGC-CAGGGGAA |  |
| 0 µg R1-7 | S |  | W | GATGCCTAACAAGC-CAGGGGAG |  |
|  |  |  | W | GATGCCTAACAAGC-CAGGGGAG |  |
|  | T |  | W | GATGCCTAACAAGC-CAGGGGAA |  |
|  |  |  | W | GATGCCTAACAAGC-CAGGGGAA |  |
| 0 µg R1-8 | S |  | W | GATGCCTAACAAGC-CAGGGGAG |  |
|  |  |  | W | GATGCCTAACAAGC-CAGGGGAG |  |
|  | T |  | W | GATGCCTAACAAGC-CAGGGGAA |  |
|  |  |  | W | GATGCCTAACAAGC-CAGGGGAA |  |
| 0 µg R1-9 | S |  | W | GATGCCTAACAAGC-CAGGGGAG |  |
|  |  |  | W | GATGCCTAACAAGC-CAGGGGAG |  |
|  | T |  | W | GATGCCTAACAAGC-CAGGGGAA |  |
|  |  |  | W | GATGCCTAACAAGC-CAGGGGAA |  |
| 0 µg R1-10 | S |  | W | GATGCCTAACAAGC-CAGGGGAG |  |
|  |  |  | W | GATGCCTAACAAGC-CAGGGGAG |  |
|  | T |  | W | GATGCCTAACAAGC-CAGGGGAA |  |
|  |  |  | W | GATGCCTAACAAGC-CAGGGGAA |  |
| 5 µg R1-1 | S |  | W | GATGCCTAACAAGC-CAGGGGAG |  |
|  |  |  | W | GATGCCTAACAAGC-CAGGGGAG |  |
|  | T |  | W | GATGCCTAACAAGC-CAGGGGAA |  |
|  |  |  | W | GATGCCTAACAAGC-CAGGGGAA |  |
| 5 µg R1-2 | S |  | W | GATGCCTAACAAGC-CAGGGGAG |  |
|  |  |  | W | GATGCCTAACAAGC-CAGGGGAG |  |
|  | T |  | W | GATGCCTAACAAGC-CAGGGGAA |  |
|  |  |  | W | GATGCCTAACAAGC-CAGGGGAA |  |
| 5 µg R1-3 | S |  | W | GATGCCTAACAAGC-CAGGGGAG |  |
|  |  |  | W | GATGCCTAACAAGC-CAGGGGAG |  |
|  | T |  | W | GATGCCTAACAAGC-CAGGGGAA |  |
|  |  |  | W | GATGCCTAACAAGC-CAGGGGAA |  |
| 5 µg R1-4 | S |  | W | GATGCCTAACAAGC-CAGGGGAG |  |
|  |  |  | W | GATGCCTAACAAGC-CAGGGGAG |  |
|  | T |  | W | GATGCCTAACAAGC-CAGGGGAA |  |
|  |  |  | W | GATGCCTAACAAGC-CAGGGGAA |  |
| 5 µg R1-5 | S |  | W | GATGCCTAACAAGC-CAGGGGAG |  |
|  |  |  | W | GATGCCTAACAAGC-CAGGGGAG |  |
|  | T |  | W | GATGCCTAACAAGC-CAGGGGAA |  |
|  |  |  | W | GATGCCTAACAAGC-CAGGGGAA |  |
| 5 µg R1-6 | S |  | W | GATGCCTAACAAGC-CAGGGGAG |  |
|  |  |  | W | GATGCCTAACAAGC-CAGGGGAG |  |
|  | T |  | W | GATGCCTAACAAGC-CAGGGGAA |  |
|  |  |  | W | GATGCCTAACAAGC-CAGGGGAA |  |
| 5 µg R1-7 | S |  | W | GATGCCTAACAAGC-CAGGGGAG |  |
|  |  |  | W | GATGCCTAACAAGC-CAGGGGAG |  |
|  | T |  | W | GATGCCTAACAAGC-CAGGGGAA |  |
|  |  |  | W | GATGCCTAACAAGC-CAGGGGAA |  |
| 5 µg R1-8 | S | Hetero | W | GATGCCTAACAAGC-CAGGGGAG |  |
|  |  |  | I | GATGCCTAACAAGCGCAGGGGAG |  |
|  | T | Hetero | W | GATGCCTAACAAGC-CAGGGGAA |  |
|  |  |  | I | GATGCCTAACAAGCTCAGGGGAA |  |
| 5 µg R1-9 | S |  | W | GATGCCTAACAAGC-CAGGGGAG |  |
|  |  |  | W | GATGCCTAACAAGC-CAGGGGAG |  |
|  | T |  | W | GATGCCTAACAAGC-CAGGGGAA |  |
|  |  |  | W | GATGCCTAACAAGC-CAGGGGAA |  |
| 5 µg R1-10 | S |  | W | GATGCCTAACAAGC-CAGGGGAG |  |
|  |  |  | W | GATGCCTAACAAGC-CAGGGGAG |  |
|  | T |  | W | GATGCCTAACAAGC-CAGGGGAA |  |
|  |  |  | W | GATGCCTAACAAGC-CAGGGGAA |  |
| 10 µg R1-1 | S |  | W | GATGCCTAACAAGC-CAGGGGAG |  |
|  |  |  | W | GATGCCTAACAAGC-CAGGGGAG |  |
|  | T |  | W | GATGCCTAACAAGC-CAGGGGAA |  |
|  |  |  | W | GATGCCTAACAAGC-CAGGGGAA |  |
| 10 µg R1-2 | S |  | W | GATGCCTAACAAGC-CAGGGGAG |  |
|  |  |  | W | GATGCCTAACAAGC-CAGGGGAG |  |
|  | T |  | W | GATGCCTAACAAGC-CAGGGGAA |  |
|  |  |  | W | GATGCCTAACAAGC-CAGGGGAA |  |
| 10 µg R1-3 | S |  | W | GATGCCTAACAAGC-CAGGGGAG |  |
|  |  |  | W | GATGCCTAACAAGC-CAGGGGAG |  |
|  | T |  | W | GATGCCTAACAAGC-CAGGGGAA |  |
|  |  |  | W | GATGCCTAACAAGC-CAGGGGAA |  |
| 10 µg R1-4 | S |  | W | GATGCCTAACAAGC-CAGGGGAG |  |
|  |  |  | W | GATGCCTAACAAGC-CAGGGGAG |  |
|  | T |  | W | GATGCCTAACAAGC-CAGGGGAA |  |
|  |  |  | W | GATGCCTAACAAGC-CAGGGGAA |  |
| 10 µg R1-5 | S | Hetero | W | GATGCCTAACAAGC-CAGGGGAG |  |
|  |  |  | I | GATGCCTAACAAGCTCAGGGGAG |  |
|  | T |  | W | GATGCCTAACAAGC-CAGGGGAA |  |
|  |  |  | W | GATGCCTAACAAGC-CAGGGGAA |  |
| 10 µg R1-6 | S | Hetero | I | GATGCCTAACAAGCTCAGGGGAG |  |
|  |  |  | I | GATGCCTAACAAGCACAGGGGAG |  |
|  | T | Hetero | W | GATGCCTAACAAGC-CAGGGGAA |  |
|  |  |  | I | GATGCCTAACAAGCTCAGGGGAA |  |
| 10 µg R1-7 | S |  | W | GATGCCTAACAAGC-CAGGGGAG |  |
|  |  |  | W | GATGCCTAACAAGC-CAGGGGAG |  |
|  | T |  | W | GATGCCTAACAAGC-CAGGGGAA |  |
|  |  |  | W | GATGCCTAACAAGC-CAGGGGAA |  |
| 10 µg R1-8 | S |  | W | GATGCCTAACAAGC-CAGGGGAG |  |
|  |  |  | W | GATGCCTAACAAGC-CAGGGGAG |  |
|  | T |  | W | GATGCCTAACAAGC-CAGGGGAA |  |
|  |  |  | W | GATGCCTAACAAGC-CAGGGGAA |  |
| 10 µg R1-9 | S |  | W | GATGCCTAACAAGC-CAGGGGAG |  |
|  |  |  | W | GATGCCTAACAAGC-CAGGGGAG |  |
|  | T |  | W | GATGCCTAACAAGC-CAGGGGAA |  |
|  |  |  | W | GATGCCTAACAAGC-CAGGGGAA |  |
| 10 µg R1-10 | S |  | W | GATGCCTAACAAGC-CAGGGGAG |  |
|  |  |  | W | GATGCCTAACAAGC-CAGGGGAG |  |
|  | T |  | W | GATGCCTAACAAGC-CAGGGGAA |  |
|  |  |  | W | GATGCCTAACAAGC-CAGGGGAA |  |
| 20 µg R1-1 | S | Hetero | I | GATGCCTAACAAGCTCAGGGGAG |  |
|  |  |  | D | GATGCCTAACAAGC---GGGGAG |  |
|  | T | Hetero | W | GATGCCTAACAAGC-CAGGGGAA |  |
|  |  |  | I | GATGCCTAACAAGCTCAGGGGAA |  |
| 20 µg R1-2 | S | Hetero | I | GATGCCTAACAAGCTCAGGGGAG |  |
|  |  |  | W | GATGCCTAACAAGC--AGGGGAG |  |
|  | T | Hetero | W | GATGCCTAACAAGC-CAGGGGAA |  |
|  |  |  | I | GATGCCTAACAAGCTCAGGGGAA |  |
| 20 µg R1-3 | S | Hetero | I | GATGCCTAACAAGCCCAGGGGAG |  |
|  |  |  | I | GATGCCTAACAAGCTCAGGGGAG |  |
|  | T |  | W | GATGCCTAACAAGC-CAGGGGAA |  |
|  |  |  | W | GATGCCTAACAAGC-CAGGGGAA |  |
| 20 µg R1-4 | S |  | W | GATGCCTAACAAGC-CAGGGGAG |  |
|  |  |  | W | GATGCCTAACAAGC-CAGGGGAG |  |
|  | T |  | W | GATGCCTAACAAGC-CAGGGGAA |  |
|  |  |  | W | GATGCCTAACAAGC-CAGGGGAA |  |
| 20 µg R1-5 | S | Hetero | I | GATGCCTAACAAGCACAGGGGAG |  |
|  |  |  | W | GATGCCTAACAAGC-CAGGGGAG |  |
|  | T |  | W | GATGCCTAACAAGC-CAGGGGAA |  |
|  |  |  | W | GATGCCTAACAAGC-CAGGGGAA |  |
| 20 µg R1-6 | S |  | W | GATGCCTAACAAGC-CAGGGGAG |  |
|  |  |  | W | GATGCCTAACAAGC-CAGGGGAG |  |
|  | T | Hetero | I | GATGCCTAACAAGCACAGGGGAA |  |
|  |  |  | W | GATGCCTAACAAGC-CAGGGGAA |  |
| 20 µg R1-7 | S | Hetero | I | GATGCCTAACAAGCACAGGGGAG |  |
|  |  |  | I | GATGCCTAACAAGCTCAGGGGAG |  |
|  | T |  | W | GATGCCTAACAAGC-CAGGGGAA |  |
|  |  |  | W | GATGCCTAACAAGC-CAGGGGAA |  |
| 20 µg R1-8 | S | Hetero | D | GATGCCTAA------CAGGGGAG |  |
|  |  |  | I | GATGCCTAACAAGCGCAGGGGAG |  |
|  | T | Hetero | W | GATGCCTAACAAGC-CAGGGGAA |  |
|  |  |  | I | GATGCCTAACAAGCGCAGGGGAA |  |
| 20 µg R1-9 | S | Hetero | I | GATGCCTAACAAGCACAGGGGAG |  |
|  |  |  | I | GATGCCTAACAAGCTCAGGGGAG |  |
|  | T | Hetero | W | GATGCCTAACAAGC-CAGGGGAA |  |
|  |  |  | D | GATGCCTAACAAGCTCAGGGGAA |  |
| 20 µg R1-10 | S |  | W | GATGCCTAACAAGC-CAGGGGAG |  |
|  |  |  | W | GATGCCTAACAAGC-CAGGGGAG |  |
|  | T |  | W | GATGCCTAACAAGC-CAGGGGAA |  |
|  |  |  | W | GATGCCTAACAAGC-CAGGGGAA |  |

| **Sample no.** | **Genome type** | **genotype** | **Mutation type** | ***NtPDS* sequences** |  |
| --- | --- | --- | --- | --- | --- |
| Wild type | S |  | W | GATGCCTAACAAGC-CAGGGGAG |  |
|  | T |  | W | GATGCCTAACAAGC-CAGGGGAA |  |
|  | | | | | |
| 0 µg R2-1 | S |  | W | GATGCCTAACAAGC-CAGGGGAG |  |
|  |  |  | W | GATGCCTAACAAGC-CAGGGGAG |  |
|  | T |  | W | GATGCCTAACAAGC-CAGGGGAA |  |
|  |  |  | W | GATGCCTAACAAGC-CAGGGGAA |  |
| 0 µg R2-2 | S |  | W | GATGCCTAACAAGC-CAGGGGAG |  |
|  |  |  | W | GATGCCTAACAAGC-CAGGGGAG |  |
|  | T |  | W | GATGCCTAACAAGC-CAGGGGAA |  |
|  |  |  | W | GATGCCTAACAAGC-CAGGGGAA |  |
| 0 µg R2-3 | S |  | W | GATGCCTAACAAGC-CAGGGGAG |  |
|  |  |  | W | GATGCCTAACAAGC-CAGGGGAG |  |
|  | T |  | W | GATGCCTAACAAGC-CAGGGGAA |  |
|  |  |  | W | GATGCCTAACAAGC-CAGGGGAA |  |
| 0 µg R2-4 | S |  | W | GATGCCTAACAAGC-CAGGGGAG |  |
|  |  |  | W | GATGCCTAACAAGC-CAGGGGAG |  |
|  | T |  | W | GATGCCTAACAAGC-CAGGGGAA |  |
|  |  |  | W | GATGCCTAACAAGC-CAGGGGAA |  |
| 0 µg R2-5 | S |  | W | GATGCCTAACAAGC-CAGGGGAG |  |
|  |  |  | W | GATGCCTAACAAGC-CAGGGGAG |  |
|  | T |  | W | GATGCCTAACAAGC-CAGGGGAA |  |
|  |  |  | W | GATGCCTAACAAGC-CAGGGGAA |  |
| 0 µg R2-6 | S |  | W | GATGCCTAACAAGC-CAGGGGAG |  |
|  |  |  | W | GATGCCTAACAAGC-CAGGGGAG |  |
|  | T |  | W | GATGCCTAACAAGC-CAGGGGAA |  |
|  |  |  | W | GATGCCTAACAAGC-CAGGGGAA |  |
| 0 µg R2-7 | S |  | W | GATGCCTAACAAGC-CAGGGGAG |  |
|  |  |  | W | GATGCCTAACAAGC-CAGGGGAG |  |
|  | T |  | W | GATGCCTAACAAGC-CAGGGGAA |  |
|  |  |  | W | GATGCCTAACAAGC-CAGGGGAA |  |
| 0 µg R2-8 | S |  | W | GATGCCTAACAAGC-CAGGGGAG |  |
|  |  |  | W | GATGCCTAACAAGC-CAGGGGAG |  |
|  | T |  | W | GATGCCTAACAAGC-CAGGGGAA |  |
|  |  |  | W | GATGCCTAACAAGC-CAGGGGAA |  |
| 0 µg R2-9 | S |  | W | GATGCCTAACAAGC-CAGGGGAG |  |
|  |  |  | W | GATGCCTAACAAGC-CAGGGGAG |  |
|  | T |  | W | GATGCCTAACAAGC-CAGGGGAA |  |
|  |  |  | W | GATGCCTAACAAGC-CAGGGGAA |  |
| 0 µg R2-10 | S |  | W | GATGCCTAACAAGC-CAGGGGAG |  |
|  |  |  | W | GATGCCTAACAAGC-CAGGGGAG |  |
|  | T |  | W | GATGCCTAACAAGC-CAGGGGAA |  |
|  |  |  | W | GATGCCTAACAAGC-CAGGGGAA |  |
| 5 µg R2-1 | S | Homo | I | GATGCCTAACAAGCGCAGGGGAG |  |
|  |  |  | I | GATGCCTAACAAGCGCAGGGGAG |  |
|  | T |  | W | GATGCCTAACAAGC-CAGGGGAA |  |
|  |  |  | W | GATGCCTAACAAGC-CAGGGGAA |  |
| 5 µg R2-2 | S |  | W | GATGCCTAACAAGC-CAGGGGAG |  |
|  |  |  | W | GATGCCTAACAAGC-CAGGGGAG |  |
|  | T |  | W | GATGCCTAACAAGC-CAGGGGAA |  |
|  |  |  | W | GATGCCTAACAAGC-CAGGGGAA |  |
| 5 µg R2-3 | S |  | W | GATGCCTAACAAGC-CAGGGGAG |  |
|  |  |  | W | GATGCCTAACAAGC-CAGGGGAG |  |
|  | T |  | W | GATGCCTAACAAGC-CAGGGGAA |  |
|  |  |  | W | GATGCCTAACAAGC-CAGGGGAA |  |
| 5 µg R2-4 | S | Hetero | I | GATGCCTAACAAGCTCAGGGGAG |  |
|  |  |  | I | GATGCCTAACAAGC(+153 bps) |  |
|  | T | Hetero | W | GATGCCTAACAAGC-CAGGGGAA |  |
|  |  |  | I | GATGCCTAACAAGCACAGGGGAA |  |
| 5 µg R2-5 | S |  | W | GATGCCTAACAAGC-CAGGGGAG |  |
|  |  |  | W | GATGCCTAACAAGC-CAGGGGAG |  |
|  | T |  | W | GATGCCTAACAAGC-CAGGGGAA |  |
|  |  |  | W | GATGCCTAACAAGC-CAGGGGAA |  |
| 5 µg R2-6 | S |  | W | GATGCCTAACAAGC-CAGGGGAG |  |
|  |  |  | W | GATGCCTAACAAGC-CAGGGGAG |  |
|  | T |  | W | GATGCCTAACAAGC-CAGGGGAA |  |
|  |  |  | W | GATGCCTAACAAGC-CAGGGGAA |  |
| 5 µg R2-7 | S |  | W | GATGCCTAACAAGC-CAGGGGAG |  |
|  |  |  | W | GATGCCTAACAAGC-CAGGGGAG |  |
|  | T |  | W | GATGCCTAACAAGC-CAGGGGAA |  |
|  |  |  | W | GATGCCTAACAAGC-CAGGGGAA |  |
| 5 µg R2-8 | S |  | W | GATGCCTAACAAGC-CAGGGGAG |  |
|  |  |  | W | GATGCCTAACAAGC-CAGGGGAG |  |
|  | T |  | W | GATGCCTAACAAGC-CAGGGGAA |  |
|  |  |  | W | GATGCCTAACAAGC-CAGGGGAA |  |
| 5 µg R2-9 | S |  | W | GATGCCTAACAAGC-CAGGGGAG |  |
|  |  |  | W | GATGCCTAACAAGC-CAGGGGAG |  |
|  | T |  | W | GATGCCTAACAAGC-CAGGGGAA |  |
|  |  |  | W | GATGCCTAACAAGC-CAGGGGAA |  |
| 5 µg R2-10 | S | Homo | I | GATGCCTAACAAGCTCAGGGGAG |  |
|  |  |  | I | GATGCCTAACAAGCTCAGGGGAG |  |
|  | T | Hetero | W | GATGCCTAACAAGC-CAGGGGAA |  |
|  |  |  | I | GATGCCTAACAAGCGCAGGGGAA |  |
| 10 µg R2-1 | S | Hetero | I | GATGCCTAACAAGCTCAGGGGAG |  |
|  |  |  | I | GATGCCTAACAAGCACAGGGGAG |  |
|  | T |  | W | GATGCCTAACAAGC-CAGGGGAA |  |
|  |  |  | W | GATGCCTAACAAGC-CAGGGGAA |  |
| 10 µg R2-2 | S |  | W | GATGCCTAACAAGC-CAGGGGAG |  |
|  |  |  | W | GATGCCTAACAAGC-CAGGGGAG |  |
|  | T |  | W | GATGCCTAACAAGC-CAGGGGAA |  |
|  |  |  | W | GATGCCTAACAAGC-CAGGGGAA |  |
| 10 µg R2-3 | S |  | W | GATGCCTAACAAGC-CAGGGGAG |  |
|  |  |  | W | GATGCCTAACAAGC-CAGGGGAG |  |
|  | T |  | W | GATGCCTAACAAGC-CAGGGGAA |  |
|  |  |  | W | GATGCCTAACAAGC-CAGGGGAA |  |
| 10 µg R2-4 | S |  | W | GATGCCTAACAAGC-CAGGGGAG |  |
|  |  |  | W | GATGCCTAACAAGC-CAGGGGAG |  |
|  | T |  | W | GATGCCTAACAAGC-CAGGGGAA |  |
|  |  |  | W | GATGCCTAACAAGC-CAGGGGAA |  |
| 10 µg R2-5 | S |  | W | GATGCCTAACAAGC-CAGGGGAG |  |
|  |  |  | W | GATGCCTAACAAGC-CAGGGGAG |  |
|  | T |  | W | GATGCCTAACAAGC-CAGGGGAA |  |
|  |  |  | W | GATGCCTAACAAGC-CAGGGGAA |  |
| 10 µg R2-6 | S |  | W | GATGCCTAACAAGC-CAGGGGAG |  |
|  |  |  | W | GATGCCTAACAAGC-CAGGGGAG |  |
|  | T |  | W | GATGCCTAACAAGC-CAGGGGAA |  |
|  |  |  | W | GATGCCTAACAAGC-CAGGGGAA |  |
| 10 µg R2-7 | S |  | W | GATGCCTAACAAGC-CAGGGGAG |  |
|  |  |  | W | GATGCCTAACAAGC-CAGGGGAG |  |
|  | T |  | W | GATGCCTAACAAGC-CAGGGGAA |  |
|  |  |  | W | GATGCCTAACAAGC-CAGGGGAA |  |
| 10 µg R2-8 | S |  | W | GATGCCTAACAAGC-CAGGGGAG |  |
|  |  |  | W | GATGCCTAACAAGC-CAGGGGAG |  |
|  | T |  | W | GATGCCTAACAAGC-CAGGGGAA |  |
|  |  |  | W | GATGCCTAACAAGC-CAGGGGAA |  |
| 10 µg R2-9 | S |  | W | GATGCCTAACAAGC-CAGGGGAG |  |
|  |  |  | W | GATGCCTAACAAGC-CAGGGGAG |  |
|  | T |  | W | GATGCCTAACAAGC-CAGGGGAA |  |
|  |  |  | W | GATGCCTAACAAGC-CAGGGGAA |  |
| 10 µg R2-10 | S |  | W | GATGCCTAACAAGC-CAGGGGAG |  |
|  |  |  | W | GATGCCTAACAAGC-CAGGGGAG |  |
|  | T |  | W | GATGCCTAACAAGC-CAGGGGAA |  |
|  |  |  | W | GATGCCTAACAAGC-CAGGGGAA |  |
| 20 µg R2-1 | S |  | W | GATGCCTAACAAGC-CAGGGGAG |  |
|  |  |  | W | GATGCCTAACAAGC-CAGGGGAG |  |
|  | T |  | W | GATGCCTAACAAGC-CAGGGGAA |  |
|  |  |  | W | GATGCCTAACAAGC-CAGGGGAA |  |
| 20 µg R2-2 | S |  | W | GATGCCTAACAAGC-CAGGGGAG |  |
|  |  |  | W | GATGCCTAACAAGC-CAGGGGAG |  |
|  | T |  | W | GATGCCTAACAAGC-CAGGGGAA |  |
|  |  |  | W | GATGCCTAACAAGC-CAGGGGAA |  |
| 20 µg R2-3 | S |  | W | GATGCCTAACAAGC-CAGGGGAG |  |
|  |  |  | W | GATGCCTAACAAGC-CAGGGGAG |  |
|  | T |  | W | GATGCCTAACAAGC-CAGGGGAA |  |
|  |  |  | W | GATGCCTAACAAGC-CAGGGGAA |  |
| 20 µg R2-4 | S | Hetero | I | GATGCCTAACAAGCTCAGGGGAG |  |
|  |  |  | W | GATGCCTAACAAGC-CAGGGGAG |  |
|  | T | Hetero | W | GATGCCTAACAAGC-CAGGGGAA |  |
|  |  |  | D | GATGCCTAACAAGCGTC(-7 bps) |  |
| 20 µg R2-5 | S |  | W | GATGCCTAACAAGC-CAGGGGAG |  |
|  |  |  | W | GATGCCTAACAAGC-CAGGGGAG |  |
|  | T |  | W | GATGCCTAACAAGC-CAGGGGAA |  |
|  |  |  | W | GATGCCTAACAAGC-CAGGGGAA |  |
| 20 µg R2-6 | S |  | W | GATGCCTAACAAGC-CAGGGGAG |  |
|  |  |  | W | GATGCCTAACAAGC-CAGGGGAG |  |
|  | T |  | W | GATGCCTAACAAGC-CAGGGGAA |  |
|  |  |  | W | GATGCCTAACAAGC-CAGGGGAA |  |
| 20 µg R2-7 | S |  | W | GATGCCTAACAAGC-CAGGGGAG |  |
|  |  |  | W | GATGCCTAACAAGC-CAGGGGAG |  |
|  | T |  | W | GATGCCTAACAAGC-CAGGGGAA |  |
|  |  |  | W | GATGCCTAACAAGC-CAGGGGAA |  |
| 20 µg R2-8 | S |  | W | GATGCCTAACAAGC-CAGGGGAG |  |
|  |  |  | W | GATGCCTAACAAGC-CAGGGGAG |  |
|  | T |  | W | GATGCCTAACAAGC-CAGGGGAA |  |
|  |  |  | W | GATGCCTAACAAGC-CAGGGGAA |  |
| 20 µg R2-9 | S | Hetero | W | GATGCCTAACAAGC-CAGGGGAG |  |
|  |  |  | I | GATGCCTAACAAGCTCAGGGGAG |  |
|  | T |  | W | GATGCCTAACAAGC-CAGGGGAA |  |
|  |  |  | W | GATGCCTAACAAGC-CAGGGGAA |  |
| 20 µg R2-10 | S | Hetero | I | GATGCCTAACAAGCGCAGGGGAG |  |
|  |  |  | I | GATGCCTAACAAGCACAGGGGAA |  |
|  | T |  | W | GATGCCTAACAAGC-CAGGGGAA |  |
|  |  |  | W | GATGCCTAACAAGC-CAGGGGAA |  |

| **Sample no.** | **Genome type** | **genotype** | **Mutation type** | ***NtPDS* sequences** |  |
| --- | --- | --- | --- | --- | --- |
| Wild type | S |  | W | GATGCCTAACAAGC-CAGGGGAG |  |
|  | T |  | W | GATGCCTAACAAGC-CAGGGGAA |  |
|  | | | | | |
| 0 µg R3-1 | S |  | W | GATGCCTAACAAGC-CAGGGGAG |  |
|  |  |  | W | GATGCCTAACAAGC-CAGGGGAG |  |
|  | T |  | W | GATGCCTAACAAGC-CAGGGGAA |  |
|  |  |  | W | GATGCCTAACAAGC-CAGGGGAA |  |
| 0 µg R3-2 | S |  | W | GATGCCTAACAAGC-CAGGGGAG |  |
|  |  |  | W | GATGCCTAACAAGC-CAGGGGAG |  |
|  | T |  | W | GATGCCTAACAAGC-CAGGGGAA |  |
|  |  |  | W | GATGCCTAACAAGC-CAGGGGAA |  |
| 0 µg R3-3 | S |  | W | GATGCCTAACAAGC-CAGGGGAG |  |
|  |  |  | W | GATGCCTAACAAGC-CAGGGGAG |  |
|  | T |  | W | GATGCCTAACAAGC-CAGGGGAA |  |
|  |  |  | W | GATGCCTAACAAGC-CAGGGGAA |  |
| 0 µg R3-4 | S |  | W | GATGCCTAACAAGC-CAGGGGAG |  |
|  |  |  | W | GATGCCTAACAAGC-CAGGGGAG |  |
|  | T |  | W | GATGCCTAACAAGC-CAGGGGAA |  |
|  |  |  | W | GATGCCTAACAAGC-CAGGGGAA |  |
| 0 µg R3-5 | S |  | W | GATGCCTAACAAGC-CAGGGGAG |  |
|  |  |  | W | GATGCCTAACAAGC-CAGGGGAG |  |
|  | T |  | W | GATGCCTAACAAGC-CAGGGGAA |  |
|  |  |  | W | GATGCCTAACAAGC-CAGGGGAA |  |
| 0 µg R3-6 | S |  | W | GATGCCTAACAAGC-CAGGGGAG |  |
|  |  |  | W | GATGCCTAACAAGC-CAGGGGAG |  |
|  | T |  | W | GATGCCTAACAAGC-CAGGGGAA |  |
|  |  |  | W | GATGCCTAACAAGC-CAGGGGAA |  |
| 0 µg R3-7 | S |  | W | GATGCCTAACAAGC-CAGGGGAG |  |
|  |  |  | W | GATGCCTAACAAGC-CAGGGGAG |  |
|  | T |  | W | GATGCCTAACAAGC-CAGGGGAA |  |
|  |  |  | W | GATGCCTAACAAGC-CAGGGGAA |  |
| 0 µg R3-8 | S |  | W | GATGCCTAACAAGC-CAGGGGAG |  |
|  |  |  | W | GATGCCTAACAAGC-CAGGGGAG |  |
|  | T |  | W | GATGCCTAACAAGC-CAGGGGAA |  |
|  |  |  | W | GATGCCTAACAAGC-CAGGGGAA |  |
| 0 µg R3-9 | S |  | W | GATGCCTAACAAGC-CAGGGGAG |  |
|  |  |  | W | GATGCCTAACAAGC-CAGGGGAG |  |
|  | T |  | W | GATGCCTAACAAGC-CAGGGGAA |  |
|  |  |  | W | GATGCCTAACAAGC-CAGGGGAA |  |
| 0 µg R3-10 | S |  | W | GATGCCTAACAAGC-CAGGGGAG |  |
|  |  |  | W | GATGCCTAACAAGC-CAGGGGAG |  |
|  | T |  | W | GATGCCTAACAAGC-CAGGGGAA |  |
|  |  |  | W | GATGCCTAACAAGC-CAGGGGAA |  |
| 5 µg R3-1 | S |  | W | GATGCCTAACAAGC-CAGGGGAG |  |
|  |  |  | W | GATGCCTAACAAGC-CAGGGGAG |  |
|  | T | Hetero | W | GATGCCTAACAAGC-CAGGGGAA |  |
|  |  |  | I | GATGCCTAACAAGCACAGGGGAA |  |
| 5 µg R3-2 | S |  | W | GATGCCTAACAAGC-CAGGGGAG |  |
|  |  |  | W | GATGCCTAACAAGC-CAGGGGAG |  |
|  | T |  | W | GATGCCTAACAAGC-CAGGGGAA |  |
|  |  |  | W | GATGCCTAACAAGC-CAGGGGAA |  |
| 5 µg R3-3 | S |  | W | GATGCCTAACAAGC-CAGGGGAG |  |
|  |  |  | W | GATGCCTAACAAGC-CAGGGGAG |  |
|  | T |  | W | GATGCCTAACAAGC-CAGGGGAA |  |
|  |  |  | W | GATGCCTAACAAGC-CAGGGGAA |  |
| 5 µg R3-4 | S | Hetero | I | GATGCCTAACAAGCTCAGGGGAG |  |
|  |  |  | I | GATGCCTAACAAGCGCAGGGGAG |  |
|  | T | Hetero | W | GATGCCTAACAAGC-CAGGGGAA |  |
|  |  |  | I | GATGCCTAACAAGCACAGGGGAA |  |
| 5 µg R3-5 | S |  | W | GATGCCTAACAAGC-CAGGGGAG |  |
|  |  |  | W | GATGCCTAACAAGC-CAGGGGAG |  |
|  | T |  | W | GATGCCTAACAAGC-CAGGGGAA |  |
|  |  |  | W | GATGCCTAACAAGC-CAGGGGAA |  |
| 5 µg R3-6 | S |  | W | GATGCCTAACAAGC-CAGGGGAG |  |
|  |  |  | W | GATGCCTAACAAGC-CAGGGGAG |  |
|  | T |  | W | GATGCCTAACAAGC-CAGGGGAA |  |
|  |  |  | W | GATGCCTAACAAGC-CAGGGGAA |  |
| 5 µg R3-7 | S |  | W | GATGCCTAACAAGC-CAGGGGAG |  |
|  |  |  | W | GATGCCTAACAAGC-CAGGGGAG |  |
|  | T | Hetero | W | GATGCCTAACAAGC-CAGGGGAA |  |
|  |  |  | I | GATGCCTAACAAGCTCAGGGGAA |  |
| 5 µg R3-8 | S |  | W | GATGCCTAACAAGC-CAGGGGAG |  |
|  |  |  | W | GATGCCTAACAAGC-CAGGGGAG |  |
|  | T |  | W | GATGCCTAACAAGC-CAGGGGAA |  |
|  |  |  | W | GATGCCTAACAAGC-CAGGGGAA |  |
| 5 µg R3-9 | S |  | W | GATGCCTAACAAGC-CAGGGGAG |  |
|  |  |  | D | GATGCCTAACAAGC--AGGGGAG |  |
|  | T |  | W | GATGCCTAACAAGC-CAGGGGAA |  |
|  |  |  | W | GATGCCTAACAAGC-CAGGGGAA |  |
| 5 µg R3-10 | S |  | W | GATGCCTAACAAGC-CAGGGGAG |  |
|  |  |  | W | GATGCCTAACAAGC-CAGGGGAG |  |
|  | T |  | W | GATGCCTAACAAGC-CAGGGGAA |  |
|  |  |  | W | GATGCCTAACAAGC-CAGGGGAA |  |
| 10 µg R3-1 | S |  | W | GATGCCTAACAAGC-CAGGGGAG |  |
|  |  |  | W | GATGCCTAACAAGC-CAGGGGAG |  |
|  | T |  | W | GATGCCTAACAAGC-CAGGGGAA |  |
|  |  |  | W | GATGCCTAACAAGC-CAGGGGAA |  |
| 10 µg R3-2 | S |  | W | GATGCCTAACAAGC-CAGGGGAG |  |
|  |  |  | W | GATGCCTAACAAGC-CAGGGGAG |  |
|  | T |  | W | GATGCCTAACAAGC-CAGGGGAA |  |
|  |  |  | W | GATGCCTAACAAGC-CAGGGGAA |  |
| 10 µg R3-3 | S |  | W | GATGCCTAACAAGC-CAGGGGAG |  |
|  |  |  | W | GATGCCTAACAAGC-CAGGGGAG |  |
|  | T |  | W | GATGCCTAACAAGC-CAGGGGAA |  |
|  |  |  | W | GATGCCTAACAAGC-CAGGGGAA |  |
| 10 µg R3-4 | S |  | W | GATGCCTAACAAGC-CAGGGGAG |  |
|  |  |  | W | GATGCCTAACAAGC-CAGGGGAG |  |
|  | T |  | W | GATGCCTAACAAGC-CAGGGGAA |  |
|  |  |  | W | GATGCCTAACAAGC-CAGGGGAA |  |
| 10 µg R3-5 | S |  | W | GATGCCTAACAAGC-CAGGGGAG |  |
|  |  |  | W | GATGCCTAACAAGC-CAGGGGAG |  |
|  | T |  | W | GATGCCTAACAAGC-CAGGGGAA |  |
|  |  |  | W | GATGCCTAACAAGC-CAGGGGAA |  |
| 10 µg R3-6 | S | Hetero | W | GATGCCTAACAAGC-CAGGGGAG |  |
|  |  |  | I | GATGCCTAACAAGCGCAGGGGAG |  |
|  | T |  | W | GATGCCTAACAAGC-CAGGGGAA |  |
|  |  |  | W | GATGCCTAACAAGC-CAGGGGAA |  |
| 10 µg R3-7 | S | Homo | I | GATGCCTAACAAGCTCAGGGGAG |  |
|  |  |  | I | GATGCCTAACAAGCTCAGGGGAG |  |
|  | T | Hetero | W | GATGCCTAACAAGC-CAGGGGAA |  |
|  |  |  | I | GATGCCTAACAAGCTCAGGGGAA |  |
| 10 µg R3-8 | S | Hetero | W | GATGCCTAACAAGC-CAGGGGAG |  |
|  |  |  | I | GATGCCTAACAAGCACAGGGGAG |  |
|  | T |  | W | GATGCCTAACAAGC-CAGGGGAA |  |
|  |  |  | W | GATGCCTAACAAGC-CAGGGGAA |  |
| 10 µg R3-9 | S |  | W | GATGCCTAACAAGC-CAGGGGAG |  |
|  |  |  | W | GATGCCTAACAAGC-CAGGGGAG |  |
|  | T |  | W | GATGCCTAACAAGC-CAGGGGAA |  |
|  |  |  | W | GATGCCTAACAAGC-CAGGGGAA |  |
| 10 µg R3-10 | S |  | W | GATGCCTAACAAGC-CAGGGGAG |  |
|  |  |  | W | GATGCCTAACAAGC-CAGGGGAG |  |
|  | T |  | W | GATGCCTAACAAGC-CAGGGGAA |  |
|  |  |  | W | GATGCCTAACAAGC-CAGGGGAA |  |
| 20 µg R3-1 | S | Hetero | I | GATGCCTAACAAGCCCAGGGGAG |  |
|  |  |  | I | GATGCCTAACAAGCTCAGGGGAG |  |
|  | T |  | W | GATGCCTAACAAGC-CAGGGGAA |  |
|  |  |  | W | GATGCCTAACAAGC-CAGGGGAA |  |
| 20 µg R3-2 | S |  | W | GATGCCTAACAAGC-CAGGGGAG |  |
|  |  |  | W | GATGCCTAACAAGC-CAGGGGAG |  |
|  | T |  | W | GATGCCTAACAAGC-CAGGGGAA |  |
|  |  |  | W | GATGCCTAACAAGC-CAGGGGAA |  |
| 20 µg R3-3 | S | Hetero | I | GATGCCTAACAAGCCCAGGGGAG |  |
|  |  |  | I | GATGCCTAACAAGCTCAGGGGAG |  |
|  | T |  | W | GATGCCTAACAAGC-CAGGGGAA |  |
|  |  |  | W | GATGCCTAACAAGC-CAGGGGAA |  |
| 20 µg R3-4 | S | Hetero | I | GATGCCTAACAAGCGCAGGGGAG |  |
|  |  |  | I | GATGCCTAACAAGCTCAGGGGAG |  |
|  | T | Hetero | W | GATGCCTAACAAGC-CAGGGGAA |  |
|  |  |  | I | GATGCCTAACAAGCTCAGGGGAA |  |
| 20 µg R3-5 | S |  | W | GATGCCTAACAAGC-CAGGGGAG |  |
|  |  |  | W | GATGCCTAACAAGC-CAGGGGAG |  |
|  | T |  | W | GATGCCTAACAAGC-CAGGGGAA |  |
|  |  |  | W | GATGCCTAACAAGC-CAGGGGAA |  |
| 20 µg R3-6 | S |  | W | GATGCCTAACAAGC-CAGGGGAG |  |
|  |  |  | W | GATGCCTAACAAGC-CAGGGGAG |  |
|  | T |  | W | GATGCCTAACAAGC-CAGGGGAA |  |
|  |  |  | W | GATGCCTAACAAGC-CAGGGGAA |  |
| 20 µg R3-7 | S |  | W | GATGCCTAACAAGC-CAGGGGAG |  |
|  |  |  | W | GATGCCTAACAAGC-CAGGGGAG |  |
|  | T |  | W | GATGCCTAACAAGC-CAGGGGAA |  |
|  |  |  | W | GATGCCTAACAAGC-CAGGGGAA |  |
| 20 µg R3-8 | S |  | W | GATGCCTAACAAGC-CAGGGGAG |  |
|  |  |  | W | GATGCCTAACAAGC-CAGGGGAG |  |
|  | T |  | W | GATGCCTAACAAGC-CAGGGGAA |  |
|  |  |  | W | GATGCCTAACAAGC-CAGGGGAA |  |
| 20 µg R3-9 | S | Hetero | I | GATGCCTAACAAGCACAGGGGAG |  |
|  |  |  | I | GATGCCTAACAAGCCCAGGGGAG |  |
|  | T | Hetero | W | GATGCCTAACAAGC-CAGGGGAA |  |
|  |  |  | I | GATGCCTAACAAGCACAGGGGAA |  |
| 20 µg R3-10 | S |  | W | GATGCCTAACAAGC-CAGGGGAG |  |
|  |  |  | W | GATGCCTAACAAGC-CAGGGGAG |  |
|  | T |  | W | GATGCCTAACAAGC-CAGGGGAA |  |
|  |  |  | W | GATGCCTAACAAGC-CAGGGGAA |  |
